# *cis*-Diastereoselective synthesis of chroman-fused tetralins as B-ring-modified analogues of brazilin

**DOI:** 10.3762/bjoc.12.280

**Published:** 2016-12-21

**Authors:** Dimpee Gogoi, Runjun Devi, Pallab Pahari, Bipul Sarma, Sajal Kumar Das

**Affiliations:** 1 Department of Chemical Sciences, Tezpur University, Napaam, Tezpur 784028, Assam, India; 2 Chemical Science and Technology Division, CSIR-North East Institute of Science & Technology, Jorhat 785006, Assam, India

**Keywords:** brazilin, chroman, epoxy-arene cyclization, natural-product-like molecules, tetralin

## Abstract

We have synthesized a series of *cis*-6a,7,8,12b-tetrahydro-6*H*-naphtho[2,1-*c*]chromen-6a-ols as B-ring-modified analogues of (±)-brazilin. A completely regio- and *cis*-diastereoselective intramolecular Friedel–Crafts epoxy–arene cyclization of 1-tetralone-derived glycidyl ethers catalyzed by Brønsted acids was used as the key step. Our worries concerning the formation of *cis*–*trans* product mixtures and their probable conversion to naphthopyran derivatives via dehydration of the tertiary hydroxy group were laid to rest. Additionally, the angular hydroxy group of one of the synthesized products has been reductively removed by a diastereoselective method which should be useful in future for preparing libraries of chroman-fused tetralins with *trans*-stereochemistry at the ring junction.

## Findings

**Figure 1:**
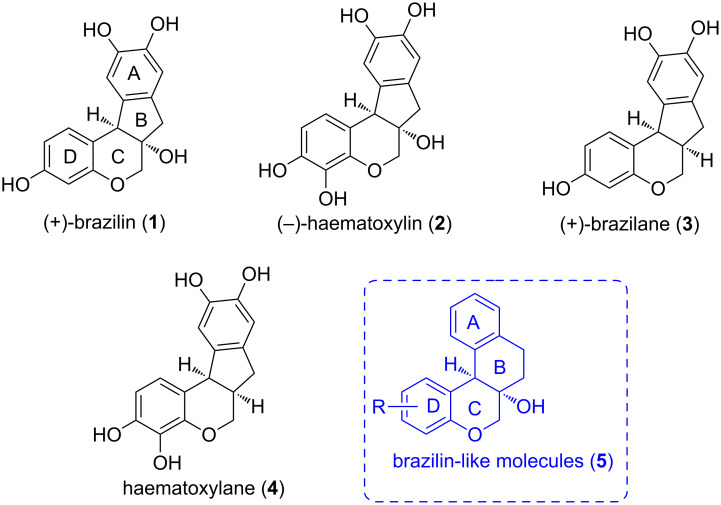
Chroman-based tetracyclic natural products **1**–**4** of the brazilin family and our designed, B-ring-modified analogues of brazilin **5**.

The chroman unit occurs widely as a privileged framework in a large number of natural products (NPs), natural product-like molecules (NPLMs) and pharmaceuticals, possessing diverse biological activities [1]. (+)-Brazilin (**1**) and (−)-haematoxylin (**2**) are two structurally-related tetracyclic homoisoflavonoid (containing a chroman unit) natural products (Figure 1) [2–4]. Their synthetic des-(angular)hydroxy derivatives (+)-brazilane (**3**) and haematoxylane (**4**) have also been mentioned in the literature [2–4]. Among them, brazilin (**1**), the most-studied member of this group, has been proved to have a number of potentially important biological activities including the abilities to act as telomerase inhibitor and to produce DNA nicks [5–7]. Also, haematoxylin has recently been demonstrated to be a potent inhibitor of protein tyrosine kinase [8]. Given the broad and interesting biological activities of these two natural products, significant efforts have been devoted for their stereoselective syntheses [9–16]. Nevertheless, many possibilities remain unexplored especially for executing new design and synthetic strategies to generate their analogues [17–18].

On the other hand, the fusion of two or more privileged scaffolds leads to geometrically well-defined rigid polycyclic structures with enhanced receptor-binding selectivity. Thus, the design and synthesis of structurally diverse, privileged structure-based polycyclic molecules with multiple chiral centers has been intensely studied during the past 15 years [19]. Moreover, on many occasions natural product-like molecules exhibit more potent biological activities than the parent natural products [20].

Meanwhile, the intramolecular Friedel–Crafts epoxy–arene (IFCEA) cyclization has been well-established as a powerful tool for the synthesis of carbo- and heterocyclic compounds [21–24]. The easy accessibility of racemic and enantiomerically pure epoxy substrates through a number of well-established epoxidation methods coupled with procedural simplicity, high atom economy, regio- and stereoselectivity of IFCEA cyclization make this methodology highly practical for the use in organic synthesis. Recently, we have reported the synthesis of diverse *trans*-4-arylchroman-3-ols via Brønsted acid catalysed regio- and stereoselective IFCEA cyclization of 2-(aryloxymethyl)-3-aryloxiranes [22–23]. The use of IFCEA cyclization as the key step is part of our long term objective of synthesizing chroman-based NPs and NPLMs and their subsequent application in medicinal chemistry.

Like chroman, the tetralin unit has been recognized as privileged structure. We were interested to synthesize *cis*-6a,7,8,12b-tetrahydro-6*H*-naphtho[2,1-*c*]chromen-6a-ols **5** (Figure 1) as B-ring-modified analogues of brazilin through the fusion of chroman and tetralin motifs to generate new bioactive molecules. To the best of our knowledge, the stereoselective synthesis of such chroman-fused tetralins has never been reported. Based on the continuous literature reports on bioactivities of brazilin, haematoxylin and their analogues and our fruitful experience [22–23,25–26] in intramolecular epoxide ring-opening chemistry including IFCEA cyclization, we became interested in the possibility of extending the IFCEA cyclization protocol to the diastereoselective synthesis of **5**. In this communication, we describe our synthetic study along this line.

**Scheme 1:**
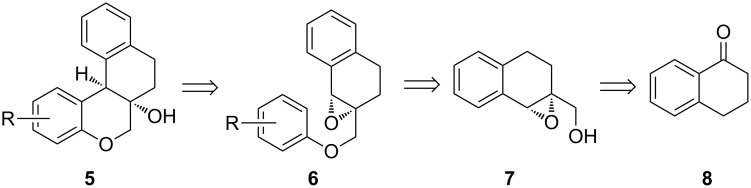
Retrosynthetic analysis of the designed B-ring-modified analogues of brazilin.

We envisioned that compounds **5** could be achieved from tetralin-based epoxy ethers **6** via the IFCEA cyclization strategy (Scheme 1). Compounds **6** could be synthesized from tetralin-based epoxy alcohols **7** and different phenols. Commercially available 1-tetralone (**8**) could be the starting materials to obtain compound **7**.

**Scheme 2:**
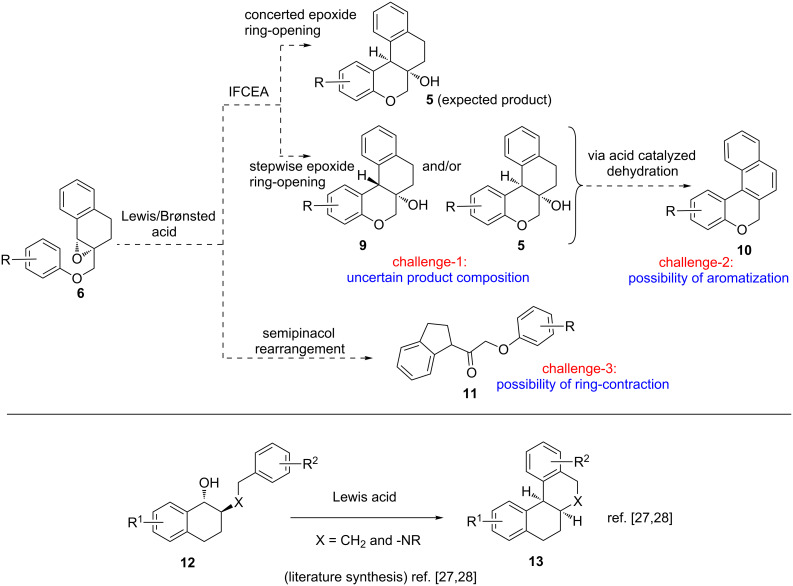
The synthetic challenge associated with the synthesis of **5** by IFCEA of **6** (above) and recent literature reports of *cis*-diastereoselective synthesis of related tetracyclic molecules via intramolecular Friedel–Crafts cyclization.

One major synthetic challenge associated with **5** is the preferential formation of the *cis*-fused [6,6] ring system over the corresponding *trans*-isomer. While the stereochemical obligation makes sure that the [6,5]-ring system in brazilin and related natural products **1**–**4** remains *cis*-fused, its corresponding [6,6]-ring system in **5** can have *cis* or *trans* stereochemistry at the ring junction. In our previous work, we observed a *cis* relationship (both in concerted or/and stepwise-epoxide ring opening) between the 4-aryl group and H atom at the C-3 position of 4-arylchroman-3-ols (thus giving rise to *trans*-4-arylchroman-3-ols) [22–23]. But it was not obvious how the planned IFCEA cyclization onto the pre-existing 6-membered ring, in case of stepwise-epoxide ring opening, would influence the product diastereoselectivity (Scheme 2, upper panel). Thus, we were concerned about the possibility of getting a mixture of **5** and **9** under the planned IFCEA cyclization of **6**. However, a literature survey indicated that *cis*-diastereoselective synthesis of related tetracyclic molecules via intramolecular Friedel–Crafts cyclization of tetralins (Scheme 2, lower panel) has been described by Lautens and co-workers [27–28].

Nonetheless, a second major issue came to our mind before the synthesis of **5** could be started. The Lewis/Brønsted acidic conditions employed during the IFCEA cyclization might activate the tertiary –OH group of **5** and/or **9**, leading to the formation of a stable 3^°^carbocation that can undergo further dehydration reactions until full aromatization to the naphthalene ring is achieved. This study would serve to help us to find the real scenario. Finally, in the presence of Lewis/Brønsted acids, substrates **6** might undergo a competitive semipinacol rearrangement to give ring contracted product **11**.

With all these concerns in our mind, we started this work first with the synthesis of compound (±)-**5a** from glycidyl ether (±)-**6a** as a model reaction for validation and optimization studies. (±)-**6a** was synthesized from commercially available 1-tetralone (**8**) in six steps (Scheme S1, Supporting Information File 1). Substrate (±)-**6a** bearing a pendant 4-methoxyphenoxy group with a moderately electron*-*rich reacting site was expected to undergo IFCEA cyclization in high regio- and diastereoselectivity to give the tetracyclic product (±)-**5a** (see the reaction scheme in Table 1). A series of reactions was carried out with varying acid catalysts, solvents, reaction temperature, reaction time as well as the amount of catalysts. The results are summarized in Table 1. We first investigated the IFCEA cyclization of (±)-**6a** using 1,1,1,3,3,3-hexafluoroisopropanol (HFIP) as reaction medium as well as the reaction promoter. The first choice of HFIP was based on its recently found ability to promote high-yielding IFCEA cyclization of benzylic epoxides [29]. Unfortunately, however, refluxing a solution of (±)-**6a** in HFIP for 4 h (entry 1, Table 1) led to the formation of a mixture of two major isolable compounds, out of which one was the expected product (±)-**5** (50%) and the remaining one was hexafluoroisopropyl ether (generated from the nucleophilic addition of HFIP on the benzylic position). Meanwhile, we found that triggering the reaction with other well-known Brønsted and Lewis acids possessing different activating abilities also led to the formation of the desired product (±)-**5a** with varying product yields (entries 1–15, Table 1).

**Table 1:** Screening of reaction conditions on the IFCEA cyclization of (±)-**6a** leading to (±)-**5a**^a^.

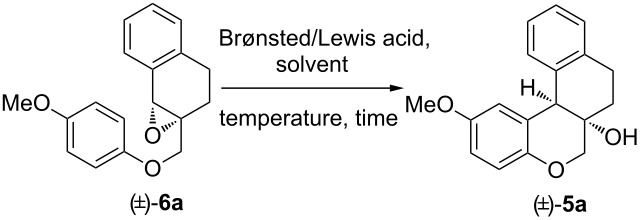					

entry	catalyst (mol %)	solvent	temp (°C)	time	yield^b^ (%)

1	–	HFIP	reflux	4 h	50
2	TsOH·H_2_O (20)	toluene	80	45 min	81
3	TsOH·H_2_O (20)	toluene	115	45 min	75
4	TsOH·H_2_O (20)	toluene	50	2 h	65
5	TsOH·H_2_O (20)	MeCN	80	45 min	79
6	TsOH·H_2_O (20)	DCE	80	45 min	70
7	TsOH·H_2_O (20)	MeNO_2_	80	45 min	75
8	TFA (20)	toluene	rt	45 min	74
9	TfOH (20)	toluene	rt	45 min	70
10	H_2_SO_4_ (20)	toluene	rt	45 min	75
11	Sc(OTf)_2_ (20)	DCM	rt	60 min	78
12	BF_3_·Et_2_O (100)	DCM	0	30 min	81
13	FeBr_3_ (20)	DCM	rt	60 min	78
14	AgSbF_6_ (20)	DCM	reflux	60 min	79
15	TiCl_4_ (20)	DCM	reflux	60 min	78

^a^Reaction conditions: (±)-**6a** (0.4 mmol), acid catalyst, solvent (8 mL). ^b^Isolated yields after silica gel column chromatography.

For example, when we conducted the reaction in toluene in the presence of 20 mol % of TsOH·H_2_O in AR-grade toluene at 80 °C (the reaction conditions that have been previously found by us [23] to be optimal for IFCEA cyclization), (±)-**5** was isolated in much better yield of 81% (Table 1, entry 2). With 20 mol % of TsOH·H_2_O in toluene, a significant increase or decrease in temperature caused noticeable changes in the product yields (Table 1, entries 3 and 4). We observed that the reaction efficiency was also dependent on the reaction medium (Table 1, entries 5–7) and toluene appeared to be the best one. Lower yields of (±)-**5** were obtained when stronger Brønsted acids like TFA, H_2_SO_4_ and TfOH were used as catalysts (Table 1, entries 8–10) in toluene as reaction medium. Among the five different Lewis acids we screened (Table 1, entries 11–15), the best result was achieved by using BF_3_·OEt_2_ (Table 1, entry 12).

Undoubtedly, the IFCEA cyclization proceeded almost equally well with different Lewis acids (Table 1, entries 11–15). However, all of these protocols required strict anhydrous conditions and some special attention to handle small amounts of the catalyst. On the other hand, TsOH·H_2_O is easily accessible, cheap, air stable, and even a minute amount can be weighed comfortably in an open atmosphere. It is important to mention that, among various acid catalyzed/promoted reactions described in the literature, on many occasions Brønsted acids have appeared as catalysts of choice under metal-free reaction conditions. On the basis of this fact and above investigations, we selected TsOH·H_2_O (20 mol %) as catalyst in toluene at 80 °C as the optimized reaction conditions.

**Figure 2:**
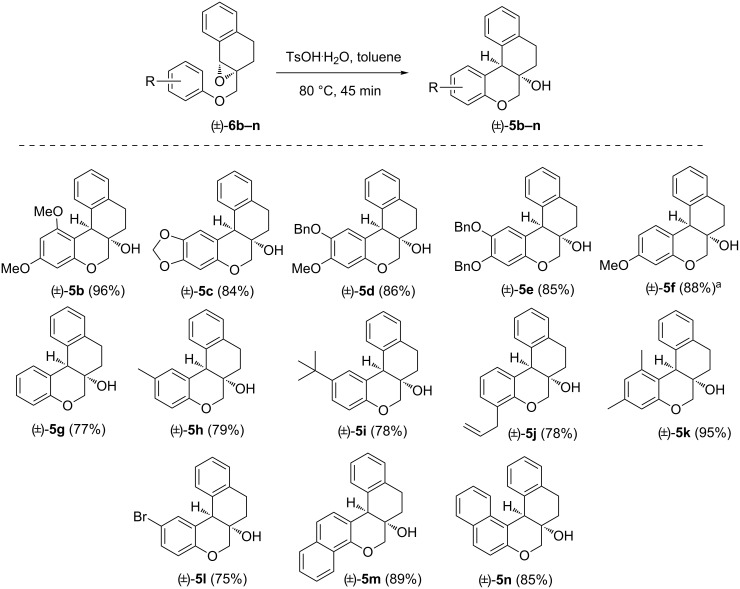
Assessment of the IFCEA cyclization on additional substrates (±)-**6b–n** leading to (±)-**5b–n**. Reaction conditions: (±)-**6b–h** (0.4 mmol), TsOH·H_2_O (20 mol %), toluene (8 mL), 80 °C, 45 min. The numbers in parentheses represent isolated yields after silica gel column chromatography. ^a^Yield of isolated mixture of the inseparable regioisomers.

With the optimized reaction conditions in hand, we next synthesized additional substrates (±)-**6b–n** with varying aryloxy groups (Scheme S1, Supporting Information File 1), and subsequently subjected them to the optimized reaction conditions with no precautions to exclude air or moisture. The results are summarized in Figure 2.

All of the synthesized substrates (±)-**6b–n** could be effectively cyclized to give the corresponding chroman-fused tetralins (±)-**5b–n**, although the product yields were varied. Among the synthesized products, high yields were observed for (±)-**5b** (96%) and (±)-**5k** (95%), attributable to the high reactivity of symmetrical 3,5-dimethoxyphenoxy and 3,5-dimethylphenoxy groups in substrates (±)-**6b** and (±)-**6k**, respectively. The synthesis of (±)-**5j** was much significant as it has an allyl group which could be functionalized by diverse alkene chemistry. We believe that a large number of molecules similar to (±)-**5j** can also be synthesized using diverse 2-allylphenols (which are easily accessible via the well-known Claisen rearrangement). Also, compound (±)-**5l** seems to have important synthetic potential for further diversification due to the presence of a bromo substituent on the chroman-arene ring. Meanwhile, we witnessed somewhat lower (75–79%), but still synthetically useful, yields of products (±)-**5g**, (±)-**5h**, (±)-**5i**, (±)-**5j** and (±)-**5l** from the corresponding substrates, bearing moderately reactive arenoxy groups. But naphthochroman-fused tetralins (±)-**5m** and (±)-**5n** were obtained again in higher yields.

**Figure 3:**
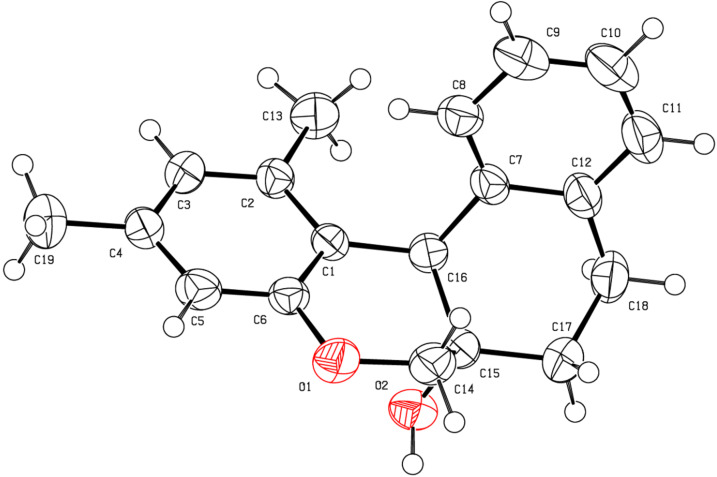
ORTEP diagram of **5k**.

It is to be mentioned that, in each of the above-mentioned IFCEA cyclization reactions, the product was entirely the *cis*-isomer and no traces of the corresponding *trans*-isomers were detected in the ^1^H NMR of the crude reaction mixtures. Stereochemical assignment at the ring junction of the products posed some initial challenge as it was not easy to confirm the *cis* relationship between the angular hydrogen and hydroxy substituents with simple 1D NMR spectroscopy. Alternative concrete evidences for the structural assignment were sought from X-ray crystallography. Fortunately, we got the single crystals of (±)-**5k** by slow evaporation of a solution of **5k** in hexane/EtOAc, and its molecular structure was confirmed by X-ray diffraction analysis (Figure 3). The ^1^H and ^13^C NMR spectra of all the products were clean (Supporting Information File 1) and the *cis*-stereochemistry at the ring junction of the remaining products were tentatively confirmed by comparing their ^1^H and ^13^C NMR spectra with that of (±)-**5k**.

The obsereved *cis*-diastereoselectivity in this work is consistent with the reported synthesis of related-tetracyclic molecules via Friedel–Crafts cyclization (Scheme 2, lower panel) promoted by metal-based Lewis acids [27–28]. Noteably, none of the synthesized molecules described in these two reports contains an angular –OH group. We feel that our synthesis involves more challenging Friedel–Crafts cyclization (Scheme 2, upper panel) and the products are relevant in the field of natural product-like molecules, because the synthesized molecules are close analogs of a natural product (brazilin). Moreover, the use of TsOH·H_2_O as an easily-accesible Brønsted acid catalyst with low loading under metal-free conditions and the operational simplicity render this transformation an attractive approach.

**Scheme 3:**
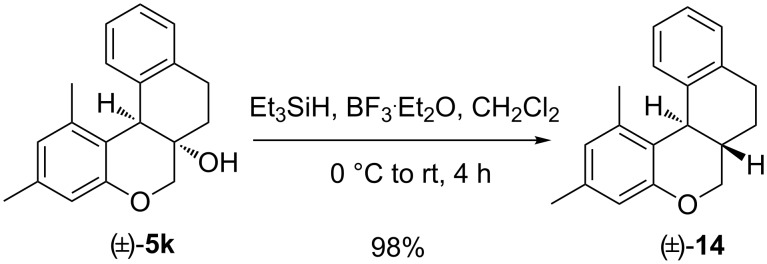
Stereoselective conversion of (±)-**5k** into (±)-**14**.

Nevertheless, in an additional work, the angular –OH group of (±)-**5k** was reductively removed on treatment with Et_3_SiH and boron trifluoride etherate to get chroman-fused tetralin (±)-**14** with the two angular H atoms being positioned in *trans* fashion (Scheme 3); no *cis*-isomer was formed. The stereochemical assignment was confirmed on the vicinal coupling constant between the two angular H atoms (the benzylic methine on the chroman ring in **14** appeared as a doublet with *J* = 10.5 Hz at δ 3.97) and by comparison with reported data of related tetracyclic molecules [30–32]. Such a stereoselective reductive removal of an –OH group should be useful in preparing a library of chroman-fused tetralins with *trans*-stereochemistry at the ring junction.

## Conclusion

In conclusion, we herein report a convenient Brønsted acid-catalyzed, metal-free, stereoselective synthesis of 6a,7,8,12b-tetrahydro-6*H*-naphtho[2,1-*c*]chromen-6a-ols as B-ring-modified analogues of brazilin using starting materials derived from inexpensive 1-tetralone and phenol derivatives. Our worries concerning the formation *cis*–*trans* mixture of 6a,7,8,12b-tetrahydro-6*H*-naphtho[2,1-*c*]chromen-6a-ols and their probable conversion to of naphthopyran derivatives via dehydration of tertiary –OH group were laid to rest. To the best of our knowledge, this is the first example of the generation of such type of chroman-fused tetralins. The easy accessibility of the starting materials, the mild reaction conditions, and the importance of products as B-ring-modified analogues of brazilin should make this synthetic work a useful addition in the diversity-oriented synthesis of natural-product like molecules. Moreover, since enantiomerically pure/enriched epoxides are compatible under IFCEA cyclization (as demonstarted previously by us [22–23] and others [29,33]), this methodology should also be applicable with the enantioenriched substrates. Further work is in progress aimed at synthesizing such type of fused hybrid molecules with different ring sizes and heteroatoms and performing mechanistic studies which will be reported in due course as a full paper.

## Supporting Information

File 1 Experimental procedures, characterization data and copies of ^1^H and ^13^C NMR spectra for final compounds.

File 2 Crystallographic data.
