# Supplementary material for: cis-Diastereoselective synthesis of chroman-fused tetralins as B-ring-modified analogues of brazilin
Source: Beilstein J Org Chem. 2016 Dec 21;12:2816–22. doi: 10.3762/bjoc.12.280 (PMC5238543; doi:10.3762/bjoc.12.280)
Supplement: File 1 — Experimental procedures, characterization data and copies of 1H and 13C NMR spectra for final compounds. [file Beilstein_J_Org_Chem-12-2816-s001.pdf]

Supporting Information

for

cis-Diastereoselective synthesis of chroman-fused tetralins as B-ring-modified analogues of brazilin

Dimpee Gogoi[‡,1], Runjun Devi[‡,1], Pallab Pahari[2], Bipul Sarma[1] and Sajal Kumar Das*[1]

Address: [1]Department of Chemical Sciences, Tezpur University, Napaam, Tezpur, Assam, India-784028 and [2]Chemical Science and Technology Division, CSIR-North East Institute of Science & Technology, Jorhat, Assam, India-785006

Email: Sajal Kumar Das*- sajalkdas@gmail.com

*Corresponding author

[‡]These two authors contributed equally to this work.

Experimental procedures, characterization data and copies of ^{1}H and ^{13}C NMR spectra for final compounds

Table of Contents

1. **General information**………………………………………………………… S2
2. **Preparation of starting materials**…………………………………………… S2
3. **Preparation of chroman-fused tetralins (±)-5a–n**…………………………… S10
4. **X ray crystallography**………………………………………………………… S16
5. **Preparation of (±)-*cis*-1,3-dimethyl-6a,7,8,12b-tetrahydro-6*H*-naphtho[2,1-*c*]chromene (10)**………………………………………………………........... S18
6. **References**……………………………………………………………………... S18
7. **Copies of ^{1}H and ^{13}C NMR spectra of all starting materials and *trans*-4-aryl-chroman-3-ols**………………………………………………………................. S19

1. General information

All dry reactions were carried out under nitrogen in oven-dried glassware using standard gastight syringes, cannulas, and septa. Commercial reagents were used without further purification unless otherwise stated. Progress of reactions was monitored by TLC on precoated Merck silica gel plates (60F-254). Visualization of reactants and products was accomplished with UV light. Column chromatography was performed over silica gel (60–120 mesh) procured from Merck using freshly distilled solvents. Melting points were determined with a Büchi-535 apparatus and are not corrected. A Perkin-Elmer 20 analyser was utilized for elemental analysis of all compounds. ^{1}H NMR and ^{13}C NMR spectra were run on a JEOL 400 MHz spectrometer in $CDCl_3$ as solvent. Tetramethylsilane (0.00 ppm) served as an internal standard in ^{1}H NMR and $CDCl_3$ (77.0 ppm) in ^{13}C NMR. All spectra were recorded at 25 °C. Coupling constants (*J* values) are given in hertz (Hz). Chemical shifts are expressed in parts per million (ppm).

2. Preparation of starting materials:

The starting epoxy ethers **(±)-6a–n** were prepared according to the Scheme S1 as shown below.

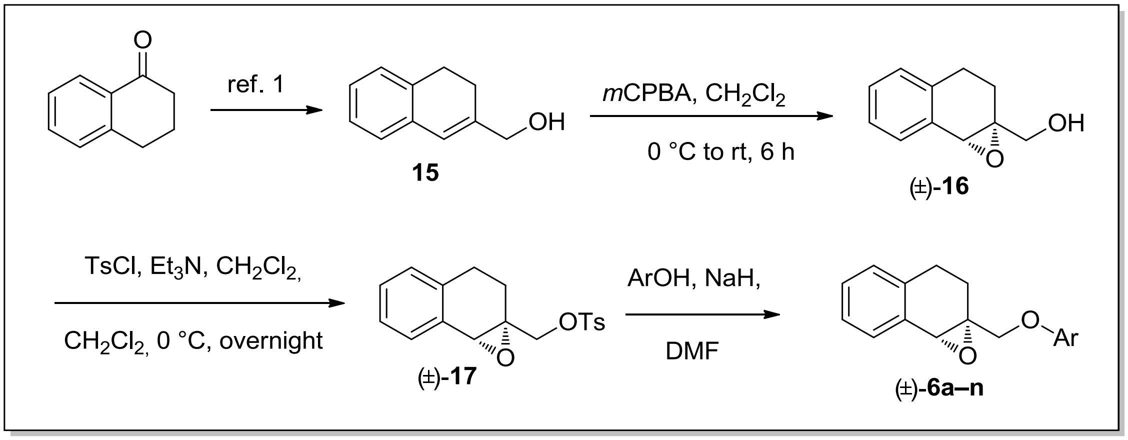

Scheme S1. Preparation of epoxy ethers **(±)-6a–n**.

E-Allylic alcohol **15** was prepared from 1-tetralone following a literature procedure [1].

(±)- (1a,2,3,7b-Tetrahydronaphtho[1,2-*b*]oxiren-1a-yl)methanol (16):

To a stirred solution of *E*-cinnamyl alcohol **16** (1.0 g, 6.24 mmol) in CH_2Cl_2 was added 3-chloroperoxybenzoic acid (70% purity, 1.62 g, 6.60 mmol) at 0 °C. The reaction mixture was stirred overnight at room temperature. The mixture was washed successively with aq solutions of Na_2SO_3 and $NaHCO_3$. The combined aqueous phases were extracted with CH_2Cl_2. The combined organic layers were dried ($MgSO_4$) and concentrated. The crude product was passed through a small pad of silica gel to obtain epoxy alcohol (±)-**16** as colourless semi-solid (0.95 g) which was quickly used for the next step without further purification.

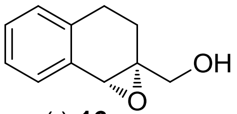

(±)-**16**

(1a,2,3,7b-Tetrahydronaphtho[1,2-*b*]oxiren-1a-yl)methyl 4-methylbenzenesulfonate (±)-17:

To a stirred solution of (±)-**16** (1.23 g, 6.98 mmol) in CH_2Cl_2 (50 mL) at 0 °C was added triethylamine (1.5 mL, 10.47 mmol) followed by tosyl chloride (2 g, 10.47 mmol) and kept in the refrigerator for 12 h. The reaction mixture was diluted with H_2O (100 mL), and extracted with CH_2Cl_2 (2 × 50 mL). The combined organic layers were washed with brine (100 mL) and dried over anhydrous Na_2SO_4. After filtration, the solvent was removed under reduced pressure. The crude product was recrystallized from EtOAc/hexane to obtain epoxy tosylate (±)-**17** (2.0 g, 90%) as off-white solid. ^{1}H NMR (400 MHz, $CDCl_3$): δ 7.80 (d, J = 8.2 Hz, 2H), 7.38-7.05 (m, 6H), 4.32 (d, J = 11.0 Hz, 1H), 4.20 (d, J = 11.0 Hz, 1H), 3.71 (s, 1H), 2.78 (td, J = 6.8, 14.7 Hz, 1H), 2.58-2.52 (m, 1H), 2.43 (s, 3H), 2.31-2.25 (m, 1H), 1.78 (td, J = 5.5, 14.0 Hz, 1H).

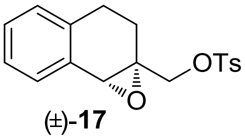

(±)-**17**

General procedure for the alkylation of different phenols with epoxy tosylate (±)-17:

To a stirred suspension of sodium hydride (35 mg, 1.5 mmol,) in DMF (3 mL), a solution of the appropriate phenol (1.0 mmol) in dry DMF (5 mL) was added at 0 °C under N_2 atmosphere. The resulting mixture was stirred for 5 min, and a solution of (±)-**17** (0.36 g, 1.1 mmol) in DMF (5 mL) was added dropwise. The solution was stirred for an additional 10 h at 0 °C. The reaction was terminated by the addition of 10% aqueous ammonium chloride (10 mL) and diethyl ether (50 mL) was added. The organic layer was separated, washed by brine (50 mL) and dried over

anhyd. Na_2SO_4. After filtration, the solution was evaporated to dryness under reduced pressure. The residue was subjected to silica gel column chromatography (with hexane/ethyl acetate as the eluent) to afford the desired tetralin-based epoxy ethers (±)-**6a–n**.

(±)-1a-((4-Methoxyphenoxy)methyl)-1a,2,3,7b-tetrahydronaphtho[1,2-*b*]oxirene (6a): Compound (±)-**6a** was prepared according to the general procedure, starting from epoxy tosylate (±)-**17** and 4-methoxyphenol. Column chromatography: 1–10% ethyl acetate in hexane. This compound was isolated as white solid (239 mg, 85%). M.p.: 95-96 °C. ^{1}H NMR (400 MHz, $CDCl_3$): δ 7.39 (d, *J* = 7.3 Hz, 1H), 7.29-7.25 (m, 1H), 7.22-7.19 (m, 1H), 7.12 (d, *J* = 7.3 Hz, 1H), 6.90 (d, *J* = 9.1 Hz, 2H), 6.84 (d, *J* = 9.1 Hz, 2H), 4.26 (d, *J* = 10.7 Hz, 1H), 4.16 (d, *J* = 10.7 Hz, 1H), 3.92 (s, 1H), 3.77 (s, 3H), 2.88 (td, *J* = 6.4, 14.9 Hz, 1H), 2.65-2.61 (m, 1H), 2.48-2.44 (m, 1H), 1.94 (td, *J* = 5.5, 14.3 Hz, 1H). ^{13}C NMR (100 MHz, $CDCl_3$): δ 152.8, 137.0, 132.0, 129.5, 128.6, 128.3, 126.2, 115.7, 114.6, 71.5, 62.5, 57.1, 55.7, 25.2, 22.9. Anal. Calcd. for $C_{18}H_{18}O_3$: C, 76.57; H, 6.43. Found: C, 76.54; H, 6.50.

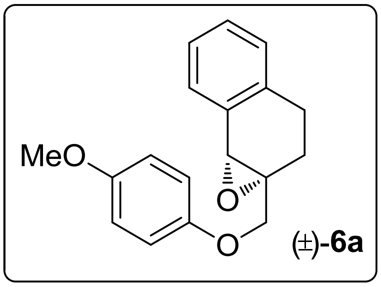

(±)-**6a**

(±)-1a-((3,5-Dimethoxyphenoxy)methyl)-1a,2,3,7b-tetrahydronaphtho[1,2-*b*]oxirene (6b): Compound (±)-**6b** was prepared according to the general procedure, starting from epoxy tosylate (±)-**17** and 3,5-dimethoxyphenol. Column chromatography: 1–10% ethyl acetate in hexane. This compound was isolated as off-white solid (281 mg, 90%). M.p.: 45-46 °C. ^{1}H NMR (400 MHz, $CDCl_3$): δ 7.40 (d, *J* = 7.3 Hz, 1H), 7.29-7.26 (m, 1H), 7.21 (t, *J* = 7.3 Hz, 1H), 7.13 (d, *J* = 7.3 Hz, 1H), 6.14 (d, *J* = 2.2 Hz, 2H), 6.10 (t, *J* = 2.2 Hz, 1H), 4.27 (d, *J* = 10.7 Hz, 1H), 4.16 (d, *J* = 10.4 Hz, 1H), 3.92 (s, 1H), 3.76 (s, 6H), 2.88 (td, *J* = 6.4, 14.7 Hz, 1H), 2.65-2.61 (m, 1H), 2.48-2.44 (m, 1H), 1.96 (td, *J* = 5.5, 14.3 Hz, 1H). ^{13}C NMR (100 MHz, $CDCl_3$): δ 161.5, 160.5, 136.9, 131.9, 129.5, 128.7, 128.3, 126.2, 93.48, 93.45, 70.7, 62.3, 57.2, 55.3, 25.2, 22.9. Anal. Calcd. for $C_{19}H_{20}O_4$: C, 73.06; H, 6.45. Found: C, 72.99; H, 6.55.

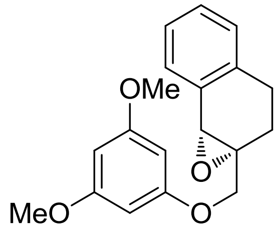

(±)-**6b**

(±)-1a-((Benzo[*d*][1,3]dioxol-5-yloxy)methyl)-1a,2,3,7b-tetrahydronaphtho[1,2-*b*]oxirene (6c):Compound (±)-**6c** was prepared according to the general procedure, starting from (±)-**17** and sesamol. Column chromatography: 1–10% ethyl acetate in hexane. This compound was isolated as white solid (260 mg, 88%). M.p.: 82-83 °C. ^{1}H NMR (400 MHz, $CDCl_3$): δ 7.41 (d, *J* = 7.3 Hz, 1H), 7.30-7.28 (m, 1H), 7.22 (t, *J* = 7.3 Hz, 1H), 7.14 (d, *J* = 7.3 Hz, 1H), 6.72 (d, *J* = 8.2 Hz, 1H), 6.52 (d, *J* = 2.7 Hz, 1H), 6.38 (dd, *J* = 2.7, 8.7 Hz, 1H), 5.93 (s, 2H), 4.24 (d, *J* = 10.5 Hz, 1H), 4.14 (d, *J* = 10.5 Hz, 1H), 3.92 (s, 1H), 2.89 (td, *J* = 6.4, 14.7 Hz, 1H), 2.67-2.61 (m, 1H), 2.49-2.44 (m, 1H), 1.94 (td, *J* = 5.5, 13.2 Hz, 1H). ^{13}C NMR (100 MHz, $CDCl_3$): δ 148.3, 136.9, 131.9, 129.5, 128.7, 128.3, 126.2, 107.9, 105.8, 101.2, 98.3, 87.1, 71.7, 62.4, 57.1, 25.2, 22.9. Anal. Calcd. for $C_{18}H_{16}O_4$: C, 72.96; H, 5.44. Found: C, 73.02; H, 5.53.

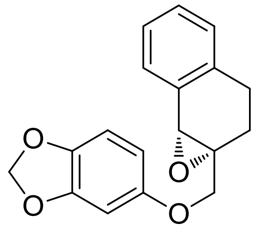
(±)-**6c**

(±)-1a-((4-(Benzyloxy)-3-methoxyphenoxy)methyl)-1a,2,3,7b-tetrahydronaphtho[1,2-*b*]oxirene (6d):

Compound (±)-**6d** was prepared according to the general procedure, starting from (±)-**17** and 3-methoxy-4-benzyloxyphenol. Column chromatography: 1–10% ethyl acetate in hexane. This compound was isolated as white solid (310 mg, 80%). M.p.: 105-106 °C. ^{1}H NMR (400 MHz, $CDCl_3$): δ 7.43 (d, *J* = 7.0 Hz, 2H), 7.41-7.35 (m, 3H), 7.32-7.27 (m, 2H), 7.22 (t, *J* = 7.3 Hz, 1H), 7.13 (d, *J* = 7.6 Hz, 1H), 6.80 (d, *J* = 8.5 Hz, 1H), 6.62 (d, *J* = 2.7 Hz, 1H), 6.38 (dd, *J* = 2.7, 8.5 Hz, 1H), 5.09 (s, 2H), 4.26 (d, *J* = 10.7 Hz, 1H), 4.15 (d, *J* = 10.7 Hz, 1H), 3.93 (s, 1H), 3.87 (s, 3H), 2.89 (td, *J* = 6.1, 14.6 Hz, 1H), 2.66-2.62 (m, 1H), 2.49-2.45 (m, 1H), 1.95 (td, *J* = 5.5, 13.2 Hz, 1H). ^{13}C NMR (100 MHz, $CDCl_3$): δ 153.8, 150.7, 142.7, 137.4, 136.9, 129.4, 128.7, 128.4, 128.3, 127.7, 127.4, 126.2, 126.1, 115.4, 104.0, 101.2, 72.0, 71.2, 62.4, 57.1, 55.9, 25.1, 22.9. Anal. Calcd. for $C_{25}H_{24}O_4$: C, 77.30; H, 6.23. Found: C, 77.35; H, 6.28.

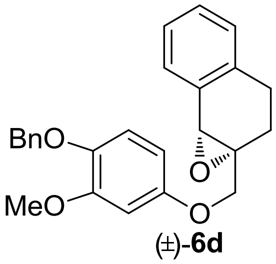

(±)-**6d**

(±)-1a-((3,4-Bis(benzyloxy)phenoxy)methyl)-1a,2,3,7b-tetrahydronaphtho[1,2-*b*]oxirene (6e):

Compound (±)**-6e** was prepared according to the general procedure, starting from (±)**-17** and 3,4-dibenzyloxyphenol. Column chromatography: 1–10% ethyl acetate in hexane. This compound was isolated as white solid (390 mg, 84%). M.p.: 102-103 °C. ^{1}H NMR (400 MHz, $CDCl_3$): δ 7.46-7.20 (m, 13H), 7.13 (d, *J* = 7.1 Hz, 1H), 6.86 (d, *J* = 8.8 Hz, 1H), 6.65 (d, *J* = 3.0 Hz, 1H), 6.42 (dd, *J* = 3.0, 8.8 Hz, 1H), 5.13 (s, 2H), 5.09 (s, 2H), 4.22 (d, *J* = 10.7 Hz, 1H), 4.11 (d, *J* = 10.7 Hz, 1H), 3.89 (s, 1H), 2.87 (td, *J* = 6.4, 14.7 Hz, 1H), 2.65-2.61 (m, 1H), 2.46-2.42 (m, 1H), 1.91 (td, *J* = 5.5, 14.0 Hz, 1H). ^{13}C NMR (100 MHz, $CDCl_3$): δ 153.9, 150.2, 143.4, 137.5, 137.0, 136.9, 132.0, 129.5, 128.7, 128.5, 128.4, 128.3, 127.8, 127.7, 127.5, 127.3, 126.2, 116.9, 105.4, 103.6, 72.5, 71.3, 71.1, 62.4, 57.1, 25.2, 22.9. Anal. Calcd. for $C_{31}H_{28}O_4$C, 80.15; H, 6.08. Found: C, 80.18; H, 6.13.

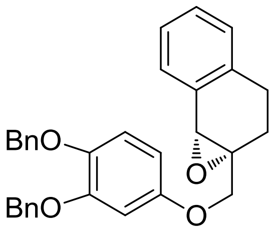

(±)-**6e**

(±)-1a-((3-Methoxyphenoxy)methyl)-1a,2,3,7b-tetrahydronaphtho[1,2-*b*]oxirene (6f):

Compound (±)**-6f** was prepared according to the general procedure, starting from epoxy tosylate (±)**-17** and 3-methoxyphenol. Column chromatography: 1–10% ethyl acetate in hexane. This compound was isolated as colourless gum (242 mg, 86%). ^{1}H NMR (400 MHz, $CDCl_3$): δ 7.40 (dd, *J* = 7.3, 1.2 Hz, 1H), 7.29-7.17 (m, 3H), 7.13 (d, *J* = 7.3 Hz, 1H), 6.55-6.52 (m, 3H), 4.28 (d, *J* = 10.7 Hz, 1H), 4.17 (d, *J* = 10.7 Hz, 1H), 3.92 (s, 1H), 3.79 (s, 3H), 2.89 (td, *J* = 6.4, 14.9 Hz, 1H), 2.66-2.62 (m, 1H), 2.50-2.45 (m, 1H), 1.95 (td, *J* = 5.5, 14.3 Hz, 1H). ^{13}C NMR (100 MHz, $CDCl_3$): δ 160.8, 159.8, 132.0, 129.9, 129.5, 128.7, 128.3, 126.2, 106.9, 106.7, 101.1, 70.7, 62.3, 57.2, 55.3, 25.2, 22.9. Anal. Calcd. for $C_{18}H_{18}O_3$: C, 76.57; H, 6.43. Found: C, 76.67; H, 6.52.

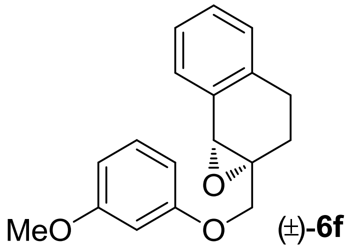

(±)-**6f**

(±)-1a-(Phenoxymethyl)-1a,2,3,7b-tetrahydronaphtho[1,2-*b*]oxirene (6g):

Compound (±)**-6g** was prepared according to the general procedure, starting from epoxy tosylate (±)**-17** and phenol. Column chromatography: 1–10% ethyl acetate in hexane. This compound

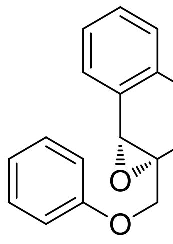

(±)-**6g**

was isolated as white solid (227 mg, 90%). M.p.: 105-106 °C. ^{1}H NMR (400 MHz, CDCl$_3$): δ 7.40 (d, *J* = 6.9 Hz, 1H), 7.31-7.19 (m, 4H), 7.13 (d, *J* = 7.3 Hz, 1H), 6.99-6.94 (m, 3H), 4.30 (d, *J* = 10.5 Hz, 1H), 4.20 (d, *J* = 10.5 Hz, 1H), 3.93 (s, 1H), 2.88 (td, *J* = 6.4, 14.7 Hz, 1H), 2.66-2.61 (m, 1H), 2.50-2.45 (m, 1H), 1.95 (td, *J* = 5.5, 13.7 Hz, 1H). ^{13}C NMR (100 MHz, CDCl$_3$): δ 158.6, 136.9, 131.9, 129.5, 128.6, 128.3, 126.2, 121.1, 114.6, 87.0, 70.6, 62.4, 57.1, 25.1, 22.9. Anal. Calcd. for $C_{17}H_{16}O_2$: C, 80.93; H, 6.39. Found: C, 80.96; H, 6.41.

(±)-1a-((*p*-Tolyloxy)methyl)-1a,2,3,7b-tetrahydronaphtho[1,2-*b*]oxirene (6h):

Compound (±)-**6h** was prepared according to the general procedure, starting from epoxy tosylate (±)-**17** and *p*-cresol. Column chromatography: 1–10% ethyl acetate in hexane. This compound was isolated as white solid (234 mg, 88%). M.p.: 95-96 °C. ^{1}H NMR (400 MHz, CDCl$_3$): δ 7.40 (d, *J* = 7.3 Hz, 1H), 7.29-7.27 (m, 1H), 7.21 (t, 1H, *J* = 7.3 Hz, 1H), 7.13 (d, *J* = 7.3 Hz, 1H), 7.09 (d, *J* = 8.7 Hz, 2H), 6.86 (d, *J* = 8.7 Hz, 2H), 4.27 (d, *J* = 10.7 Hz, 1H), 4.18 (d, *J* = 10.7 Hz, 1H), 3.93 (s, 1H), 2.89 (td, *J* = 6.4, 15.2 Hz, 1H), 2.66-2.61 (m, 1H), 2.49-2.45 (m, 1H), 2.29 (s, 3H), 1.94 (td, *J* = 5.5, 14.3 Hz, 1H). ^{13}C NMR (100 MHz, CDCl$_3$): δ 156.5, 137.0, 132.0, 130.4, 129.9, 129.5, 128.6, 128.3, 126.2, 114.5, 70.8, 62.4, 57.2, 25.2, 22.9, 20.5. Anal. Calcd. for $C_{18}H_{18}O_2$: C, 81.17; H, 6.81. Found: C, 81.26; H, 6.88.

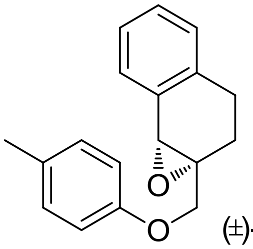

(±)-**6h**

(±)-1a-((4-(*tert*-Butyl)phenoxy)methyl)-1a,2,3,7b-tetrahydronaphtho[1,2-*b*]oxirene (6i):

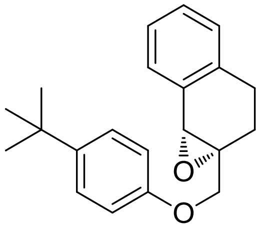

(±)-**6i**

Compound (±)-**6i** was prepared according to the general procedure, starting from epoxy tosylate (±)-**17** and 4-*tert*-butylphenol. Column chromatography: 1–10% ethyl acetate in hexane. This compound was isolated as white solid (262 mg, 85%). M.p.: 98-99 °C. ^{1}H NMR (400 MHz, CDCl$_3$): δ 7.40 (d, *J* = 7.3 Hz, 1H), 7.32-7.25 (m, 3H), 7.21 (t, *J* = 7.3 Hz, 1H), 7.12 (d, *J* = 7.3 Hz, 1H), 6.89 (d, *J* = 8.8 Hz, 2H), 4.28 (d, *J* = 10.7 Hz, 1H), 4.19 (d, *J* = 10.7 Hz, 1H), 3.92 (s, 1H), 2.88 (td, *J* = 6.4, 15.0 Hz, 1H), 2.65-2.61 (m, 1H), 2.49-2.45 (m, 1H), 1.95 (td, *J* =

5.5, 14.0 Hz, 1H), 1.3 (s, 9H). ^{13}C NMR (100 MHz, $CDCl_3$): δ 156.4, 143.8, 137.0, 132.0, 129.5, 128.6, 128.3, 126.3, 126.2, 114.0, 70.8, 62.4, 57.2, 34.1, 31.5, 25.2, 22.9. Anal. Calcd. for $C_{21}H_{24}O_2$: C, 81.78; H, 7.84. Found: C, 81.82; H, 7.77.

(±)-1a-((2-Allylphenoxy)methyl)-1a,2,3,7b-tetrahydronaphtho[1,2-*b*]oxirene (6j):

Compound (±)**-6j** was prepared according to the general procedure, starting from epoxy tosylate (±)**-17** and 2-allylphenol. Column chromatography: 1–10% ethyl acetate in hexane. This compound was isolated as colourless semi-solid (234 mg, 80%). ^{1}H NMR (400 MHz, $CDCl_3$): δ 7.39 (dd, *J* = 7.3, 1.2 Hz, 1H), 7.28-7.21 (m, 5H), 6.92 (t, *J* = 7.3 Hz, 1H), 6.86 (d, *J* = 7.9 Hz, 1H), 6.05-5.94 (m, 1H), 5.09-5.02 (m, 2H), 4.28 (d, *J* = 10.4 Hz, 1H), 4.19 (d, *J* = 10.9 Hz, 1H), 3.92 (s, 1H), 3.43 (d, *J* = 6.7 Hz, 1H), 2.88 (td, *J* = 6.4, 15.0 Hz, 1H), 2.65-2.60 (m, 1H), 2.49-2.45 (m, 1H), 1.94 (td, *J* = 5.5, 14.0 Hz, 1H). ^{13}C NMR (100 MHz, $CDCl_3$): δ 156.1, 136.9, 136.8, 132.0, 129.8, 129.4, 128.8, 128.6, 128.3, 127.3, 126.1, 121.0, 115.5, 111.4, 70.8, 62.4, 57.1, 34.4, 25.2, 22.9. Anal. Calcd. for $C_{20}H_{20}O_2$: C, 82.16; H, 6.89. Found: C, 82.01; H, 6.97.

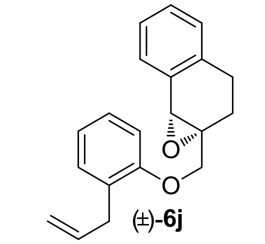
(±)-**6j**

(±)-1a-((3,5-Dimethylphenoxy)methyl)-1a,2,3,7b-tetrahydronaphtho[1,2-*b*]oxirene (6k):

Compound (±)**-6k** was prepared according to the general procedure, starting from epoxy tosylate (±)**-17** and 3,5-dimethylphenol. Column chromatography: 1–10% ethyl acetate in hexane. This compound was isolated as a colourless semi-solid (250 mg, 89%). ^{1}H NMR (400 MHz, $CDCl_3$): δ 7.39 (d, *J* = 7.3 Hz, 1H), 7.29-7.19 (m, 2H), 7.12 (d, *J* = 7.3 Hz, 1H), 6.62 (s, 1H), 6.58 (s, 2H), 4.26 (d, *J* = 10.5 Hz, 1H), 4.17 (d, *J* = 10.9 Hz, 1H), 3.91 (s, 1H), 2.88 (td, *J* = 6.4, 15.6 Hz, 1H), 2.65-2.60 (m, 1H), 2.48-2.42 (m, 1H), 2.28 (s, 6H), 1.94 (td, *J* = 5.5, 14.2 Hz, 1H). ^{13}C NMR (100 MHz, $CDCl_3$): δ 158.7, 139.2, 136.9, 132.0, 129.5, 128.6, 128.3, 126.1, 122.9, 112.4, 70.6, 62.4, 57.2, 25.2, 22.9, 21.4. Anal. Calcd. for $C_{19}H_{20}O_2$: C, 81.40; H, 7.19. Found: C, 81.44; H, 7.22.

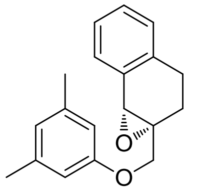
(±)-**6k**

(±)-1a-((4-Bromophenoxy)methyl)-1a,2,3,7b-tetrahydronaphtho[1,2-b]oxirene (6l):

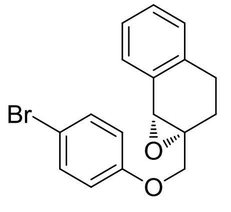
(±)-**6l**

Compound (±)-**6l** was prepared according to the general procedure, starting from epoxy tosylate (±)-**17** and 4-bromophenol. Column chromatography: 1–10% ethyl acetate in hexane. This compound was isolated as a colourless semi-solid (265 mg, 80%). ^{1}H NMR (400 MHz, $CDCl_3$): δ 7.42-7.38 (m, 3H), 7.31-7.28 (m, 1H), 7.23 (t, *J* = 7.3 Hz, 1H), 7.14 (d, *J* = 7.3 Hz, 1H), 6.85 (d, *J* = 8.7 Hz, 2H), 4.30 (d, *J* = 10.5 Hz, 1H), 4.17 (d, *J* = 10.9 Hz, 1H), 3.93 (s, 1H), 2.89 (td, *J* = 6.4, 15.1 Hz, 1H), 2.68-2.62 (m, 1H), 2.50-2.44 (m, 1H), 1.94 (td, *J* = 5.5, 14.2 Hz, 1H). Anal. Calcd. for $C_{17}H_{15}BrO_2$: C, 61.65; H, 4.56. Found: C, 61.56; H, 4.62.

(±)-1a-((Naphthalen-1-yloxy)methyl)-1a,2,3,7b-tetrahydronaphtho[1,2-*b*]oxirene (6m):

Compound (±)-**6m** was prepared according to the general procedure, starting from epoxy tosylate (±)-**17** and 1-naphthol. Column chromatography: 1–10% ethyl acetate in hexane. This compound was isolated as white solid (236 mg, 78%). M.p.: 97-98 °C. ^{1}H NMR (400 MHz, $CDCl_3$): δ 8.31-8.29 (m, 1H), 7.81-7.79 (m, 1H), 7.51-7.42 (m, 4H), 7.37 (t, *J* = 7.9 Hz, 1H), 7.31-7.21 (m, 2H), 7.15 (d, *J* = 7.6 Hz, 1H), 6.84 (d, *J* = 7.3 Hz, 1H), 4.45 (d, *J* = 10.4 Hz, 1H), 4.38 (d, *J* = 10.4 Hz, 1H), 4.01 (s, 1H), 2.94 (td, *J* = 6.4, 14.7 Hz, 1H), 2.70-2.65 (m, 1H), 2.60-2.56 (m, 1H), 2.07 (td, *J* = 5.5, 13.7 Hz, 1H). ^{13}C NMR (100 MHz, $CDCl_3$): δ 154.3, 136.9, 134.5, 132.0, 129.6, 128.7, 128.4, 127.5, 126.5, 126.2, 125.7, 125.5, 125.3, 122.0, 120.8, 104.9, 71.1, 62.4, 57.4, 25.2, 23.1. Anal. Calcd. for $C_{21}H_{18}O_2$: C, 83.42; H, 6.00. Found: C, 83.49; H, 6.02.

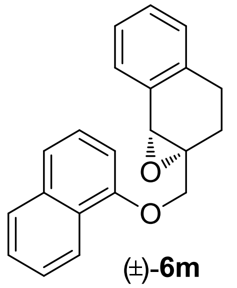
(±)-**6m**

(±)-1a-((Naphthalen-2-yloxy)methyl)-1a,2,3,7b-tetrahydronaphtho[1,2-*b*]oxirene (6n):

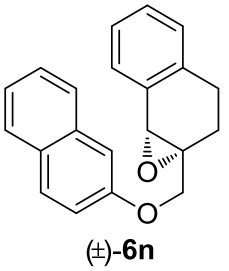
(±)-**6n**

Compound (±)-**6n** was prepared according to the general procedure, starting from epoxy tosylate (±)-**17** and 2-naphthol. Column chromatography: 1–10% ethyl acetate in hexane. This compound was isolated as white solid (218 mg, 72%). M.p.: 148-149 °C. ^{1}H NMR (400 MHz, $CDCl_3$): δ 7.79-7.83 (m, 3H), 7.47-7.42 (m, 2H), 7.37-7.28 (m, 2H), 7.25-7.20 (m, 3H), 7.16 (d, *J* = 7.3 Hz, 1H), 4.44 (d, *J* = 10.4 Hz,

1H), 4.33 (d, J = 10.4 Hz, 1H), 4.01 (s, 1H), 2.93 (td, J = 6.4, 14.4 Hz, 1H), 2.69-2.65 (m, 1H), 2.56-2.52 (m, 1H), 2.01 (td, J = 5.5, 13.7 Hz, 1H). ^{13}C NMR (100 MHz, $CDCl_3$): δ 156.6, 137.0, 134.4, 132.0, 129.55, 129.50, 129.1, 128.7, 128.4, 127.6, 126.8, 126.4, 126.2, 70.7, 62.4, 57.2, 25.2, 23.0. Anal. Calcd. for $C_{21}H_{18}O_2$: C, 83.42; H, 6.00. Found: C, 83.46; H, 5.92.

3. Preparation of chroman-fused tetralins (±)-5a–n

General procedure for the IFCEA cyclization reactions:

To a stirred solution of glycidyl ethers (±)**-6** (0.4 mmol) in AR grade toluene (8 mL) was added TsOH·H_2O (16 mg, 0.084 mmol). The resulting mixture was then heated at 80 °C. When the reaction was completed (approx. 45 min), the mixture was cooled to room temperature, and then poured in an beaker containing EtOAc (30 mL) and saturated aq $NaHCO_3$ solution (25 mL) with vigorous stirring. The combined organic layer was washed with brine (30 mL) and dried over anhydrous Na_2SO_4. After filtration, the solution was evaporated to dryness under reduced pressure. The residue was subjected to silica gel column chromatography (with hexane/ethyl acetate as the eluent) to afford the desired chroman-fused tetralins (±)**-5a–n**.

(±)-*cis*-2-Methoxy-6a,7,8,12b-tetrahydro-6*H*-naphtho[2,1-*c*]chromen-6a-ol (5a):
Compound (±)**-5a** was prepared according to the general procedure, starting from (±)**-6a**.

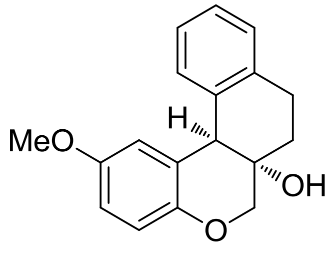

(±)-**5a**

Column chromatography: 5–12% ethyl acetate in hexane. Isolated as colourless gum (92 mg, 81%). ^{1}H NMR (400 MHz, $CDCl_3$): δ 7.22-7.13 (m, 4H), 6.86 (d, J = 8.7 Hz, 1H), 6.79 (dd, J = 2.7, 8.7 Hz, 1H), 6.72 (d, J = 2.7 Hz, 1H), 3.91-3.87 (m, 2H), 3.75-3.72 (m, 4H), 3.07-2.99 (m, 1H), 2.83-2.76 (m, 1H), 2.31 (s, 1H), 2.15-2.09 (m, 1H), 1.82-1.77 (m, 1H). ^{13}C NMR (100 MHz, $CDCl_3$): δ 153.7, 146.8, 136.9, 136.8, 129.1, 128.0, 126.9, 126.2, 122.0, 117.5, 116.2, 114.3, 71.4, 68.1, 55.7, 47.6, 31.5, 26.4. Anal. Calcd. for $C_{18}H_{18}O_3$: C, 76.57; H, 6.43. Found: C, 76.68; H, 6.48.

(±)-*cis*-1,3-Dimethoxy-6a,7,8,12b-tetrahydro-6*H*-naphtho[2,1-*c*]chromen-6a-ol (5b):

Compound (±)-**5b** was prepared according to the general procedure, starting from (±)-**6b**. Column chromatography: 5–15% ethyl acetate in hexane. Isolated as light-brown solid (120 mg, 96%). M.p.: 155-156 °C. ^{1}H NMR (400 MHz, $CDCl_3$): δ 7.15 (d, *J* = 3.9 Hz, 2H), 7.10-7.07 (m, 1H), 6.77 (d, *J* = 7.6 Hz, 1H), 6.21 (d, *J* = 2.4 Hz, 1H), 6.14 (d, *J* = 2.4 Hz, 1H), 3.94 (s, 1H), 3.81 (s, 3H), 3.78 (dd, *J* = 2.7, 11.3 Hz, 1H), 3.75 (s, 3H), 3.51 (d, *J* = 11.3 Hz, 1H), 3.10-3.03 (m, 1H), 2.81-2.75 (m, 1H), 2.39 (s, 1H), 2.31-2.26 (m, 1H), 1.57-1.51 (m, 1H). ^{13}C NMR (100 MHz, $CDCl_3$): δ 160.5, 160.3, 154.3, 138.5, 137.2, 127.8, 127.0, 126.2, 126.1, 101.8, 93.2, 92.0, 70.1, 68.2, 55.4, 55.3, 41.5, 33.3, 27.1. Anal. Calcd. for $C_{19}H_{20}O_4$: C, 73.06; H, 6.45. Found: C, 73.09; H, 6.35.

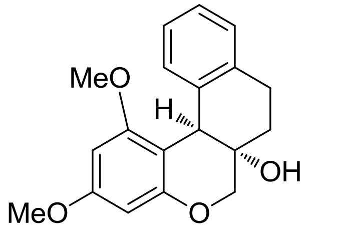

(±)-**5b**

(±)-*cis*-6,6a,7,13b-Tetrahydro-5*H*-[1,3]dioxolo[4,5-*g*]naphtho[2,1-*c*]chromen-6a-ol (5c):

Compound (±)-**5c** was prepared according to the general procedure, starting from (±)-**6c**. Column chromatography: 5–15% ethyl acetate in hexane. Isolated as colourless semi-solid (100 mg, 84%). ^{1}H NMR (400 MHz, $CDCl_3$): δ 7.21-7.18 (m, 3H), 7.13-7.11 (m, 1H), 6.60 (s, 1H), 6.46 (s, 1H), 5.92 (s, 2H), 3.87 (dd, *J* = 1.6, 10.6 Hz, 1H), 3.78 (s, 1H), 3.68 (d, *J* = 11.0 Hz, 1H), 3.04-2.97 (m, 1H), 2.80-2.75 (m, 1H), 2.14-2.09 (m, 1H), 1.79-1.74 (m, 1H). ^{13}C NMR (100 MHz, $CDCl_3$): δ 147.7, 147.4, 141.9, 137.4, 136.9, 128.9, 127.9, 126.8, 126.2, 112.5, 110.0, 101.1, 98.5, 71.3, 68.1, 47.3, 31.6, 26.4. Anal. Calcd. for $C_{18}H_{16}O_4$: C, 72.96; H, 5.44. Found: C, 73.08; H, 5.49.

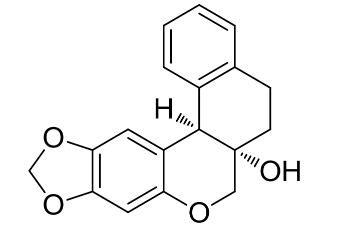

(±)-**5c**

(±)-*cis*-2-(Benzyloxy)-3-methoxy-6a,7,8,12b-tetrahydro-6*H*-naphtho[2,1-*c*]chromen-6a-ol (5d):

Compound (±)-**5d** was prepared according to the general procedure, starting from (±)-**6d**. Column chromatography: 5–15% ethyl acetate in hexane. Isolated as colourless white solid (133 mg, 86%). M.p.: 103-104 °C. ^{1}H NMR (400 MHz, $CDCl_3$): δ 7.39-7.30 (m, 5H), 7.19-7.14 (m, 2H), 7.09-7.06 (m, 1H), 6.83 (d, *J* = 7.6 Hz, 1H), 6.64 (s, 1H), 6.49 (s, 2H), 5.10-5.04 (m, 2H),

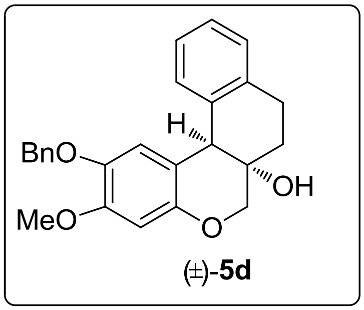

3.87 (s, 3H), 3.85 (dd, *J* = 1.8, 11.0 Hz, 1H), 3.70 (s, 1H), 3.65 (d, *J* = 11.0 Hz, 1H), 3.01-2.95 (m, 1H), 2.78-2.72 (m, 1H), 2.32 (s br, 1H), 2.13-2.08 (m, 1H), 1.73-1.67 (m, 1H). ^{13}C NMR (100 MHz, $CDCl_3$): δ 150.1, 147.3, 141.9, 137.4, 137.2, 136.7, 128.6, 128.4, 127.8, 127.7, 127.5, 126.6, 126.1, 118.1, 111.4, 100.9, 71.7, 71.1, 68.1, 55.9, 46.6, 31.7, 26.4. Anal. Calcd. for $C_{25}H_{24}O_4$: C, 77.30; H, 6.23. Found: C, 77.20; H, 6.16.

(±)-*cis*-2,3-Bis(benzyloxy)-6a,7,8,12b-tetrahydro-6*H*-naphtho[2,1-*c*]chromen-6a-ol (5e): Compound (±)-**5e** was prepared according to the general procedure, starting from (±)-**6e**. Column chromatography: 5–15% ethyl acetate in hexane. Isolated as colourless semi-solid (157 mg, 85%). ^{1}H NMR (400 MHz, $CDCl_3$): δ 7.50-7.30 (m, 10H), 7.21-7.09 (m, 3H), 6.88 (d, *J* = 7.3 Hz, 1H), 6.71 (s, 1H), 6.55 (s, 1H), 5.15 (s, 2H), 5.13-5.06 (m, 2H), 3.83 (dd, *J* = 1.8, 11.0 Hz, 1H), 3.71 (s, 1H), 3.64 (d, *J* = 10.4 Hz, 1H), 3.02-2.95 (m, 1H), 2.79-2.72 (m, 1H), 2.28 (s br, 1H), 2.13-2.07 (m, 1H), 1.74-1.66 (m, 1H). ^{13}C NMR (100 MHz, $CDCl_3$): δ 149.5, 147.5, 142.4, 137.33, 137.31, 136.9, 136.7, 128.7, 128.5, 128.4, 127.83, 127.80, 127.7, 127.6, 127.2, 126.6, 126.1, 119.4, 112.3, 103.0, 72.2, 71.0, 70.8, 68.0, 46.6, 31.6, 26.4. Anal. Calcd. for $C_{31}H_{28}O_4$: C, 80.15; H, 6.08. Found: C, 80.22; H, 6.16.

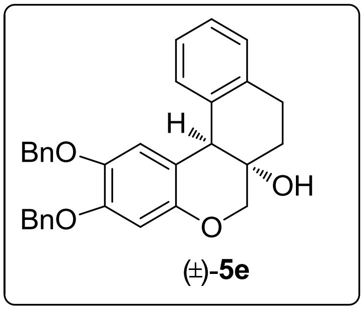

(±)-*cis*-3-Methoxy-6a,7,8,12b-tetrahydro-6*H*-naphtho[2,1-*c*]chromen-6a-ol (5f): Compound (±)-**5f** was prepared according to the general procedure, starting from (±)-**6f**. Column chromatography: 5–15% ethyl acetate in hexane. This compound was obtained as a ca. 3:1 mixture of inseparable regioisomers as judged by ^{1}H NMR analysis. Colourless semi-solid (99 mg, 88%). Major isomer (**5f**): ^{1}H NMR (400 MHz, $CDCl_3$): δ 7.19-7.10 (m, 4H), 7.05 (d, *J* = 8.5 Hz, 1H), 6.55 (dd, *J* = 2.5, 8.6 Hz, 1H), 6.47 (d, *J* = 2.5 Hz, 1H), 3.92 (dd, *J* = 1.5, 11.0 Hz, 1H), 3.82 (s, 1H), 3.78-3.76 (m, 4H), 3.05-2.98 (m, 1H), 2.88 (s, 1H), 2.81-2.76 (m, 1H), 2.13-2.08 (m, 1H), 1.82-1.77 (m, 1H). ^{13}C NMR (100 MHz, $CDCl_3$): δ 159.7, 153.6, 137.2, 136.5, 132.1,

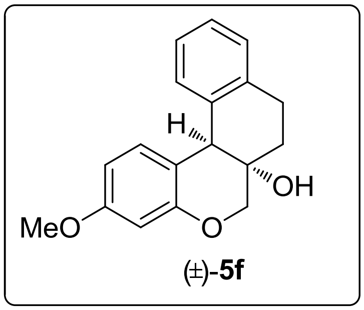

129.1, 127.9, 126.6, 126.03, 126.01, 107.8, 101.4, 71.3, 67.9, 55.2, 46.6, 31.3, 26.2. Anal. Calcd. for $C_{18}H_{18}O_3$: C, 76.57; H, 6.43. Found: C, 76.52; H, 6.51.

(±)-*cis*-6a,7,8,12b-Tetrahydro-6*H*-naphtho[2,1-*c*]chromen-6a-ol (5g):

Compound (±)-**5g** was prepared according to the general procedure, starting from (±)-**6g**. Column chromatography: 5–15% ethyl acetate in hexane. This compound was obtained as a

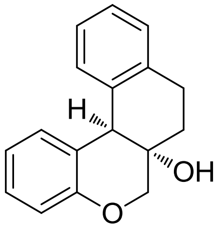

(±)-**5g**

colourless semi-solid (77 mg, 77%). ^{1}H NMR (400 MHz, $CDCl_3$): δ 7.24-7.16 (m, 5H), 7.12-7.09 (m, 1H), 6.97-6.91 (m, 2H), 3.95 (dd, *J* = 1.8, 11.0 Hz, 1H), 3.90 (s, 1H), 3.80 (d, *J* = 11.0 Hz, 1H), 3.09-2.99 (m, 1H), 2.82-2.76 (m, 1H), 2.33 (br s, 1H), 2.15-2.08 (m, 1H), 1.85-1.77 (m, 1H). ^{13}C NMR (100 MHz, $CDCl_3$): δ 148.2, 136.9, 132.0, 129.5, 128.7, 129.5, 128.7, 128.3, 126.2, 105.8, 101.2, 87.1, 71.7, 62.4, 57.1, 25.2, 22.9. Anal. Calcd. for $C_{17}H_{16}O_2$: C, 80.93; H, 6.39. Found: C, 80.99; H, 6.33.

(±)-*cis*-2-Methyl-6a,7,8,12b-tetrahydro-6*H*-naphtho[2,1-*c*]chromen-6a-ol (5h):

Compound (±)-**5h** was prepared according to the general procedure, starting from (±)-**6h**.

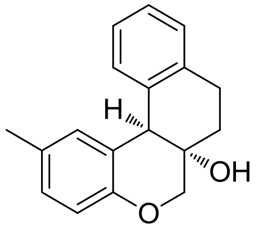

(±)-**5h**

Column chromatography: 5–15% ethyl acetate in hexane. This compound was obtained as a white solid (84 mg, 79%). M.p.: 110-112 °C. ^{1}H NMR (400 MHz, $CDCl_3$): δ 7.22-7.17 (m, 3H), 7.11-7.10 (m, 1H), 7.01 (dd, *J* = 1.8, 8.5 Hz, 1H), 6.96 (s, 1H), 6.82 (d, *J* = 8.5 Hz, 1H), 3.90 (dd, *J* = 1.5, 11.0 Hz, 1H), 3.85 (s, 1H), 3.75 (d, *J* = 11.0 Hz, 1H), 3.05-2.98 (m, 1H), 2.81-2.76 (m, 1H), 2.28 (s, 3H), 2.27 (br s, 1H), 2.14-2.09 (m, 1H), 1.82-1.76 (m, 1H). ^{13}C NMR (100 MHz, $CDCl_3$): δ 150.6, 137.1, 136.7, 131.9, 130.0, 129.2, 129.0, 127.9, 126.7, 126.0, 120.8, 116.6, 71.2, 68.1, 47.2, 31.5, 26.3, 20.6. Anal. Calcd. for $C_{18}H_{18}O_2$: C, 81.17; H, 6.81. Found: C, 81.11; H, 6.76.

(±)-*cis*-2-(*tert*-Butyl)-6a,7,8,12b-tetrahydro-6*H*-naphtho[2,1-*c*]chromen-6a-ol (5i):

Compound (±)-**5i** was prepared according to the general procedure, starting from (±)-**6i**. Column chromatography: 5–15% ethyl acetate in hexane. This compound was obtained as a colourless

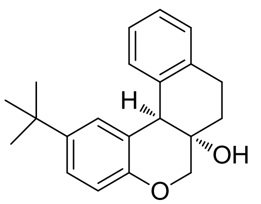

(±)-**5i**

gum (96 mg, 78%). ^{1}H NMR (400 MHz, $CDCl_3$): δ 7.21-7.14 (m, 5H), 7.03 (d, *J* = 6.7 Hz, 1H), 6.84 (d, *J* = 8.5 Hz, 1H), 3.88-3.83 (m, 2H), 3.85 (s, 1H), 3.65 (d, *J* = 10.4 Hz, 1H), 3.04-2.98 (m, 1H), 2.79-2.74 (m, 1H), 2.48 (br s, 3H), 2.15-2.10 (m, 1H), 1.71-1.65 (m, 1H), 1.28 (s, 9H). ^{13}C NMR (100 MHz, $CDCl_3$): δ 150.5, 143.4, 137.6, 137.0, 128.8, 128.7, 127.7, 126.6, 126.0, 125.4, 119.9, 116.2, 71.0, 68.3, 47.2, 34.0, 31.8, 31.4, 26.5. Anal. Calcd. for $C_{21}H_{24}O_2$: C, 81.78; H, 7.84. Found: C, 81.85; H, 7.92.

(±)-*cis*-4-Allyl-6a,7,8,12b-tetrahydro-6*H*-naphtho[2,1-*c*]chromen-6a-ol (5j):
Compound (±)-**5j** was prepared according to the general procedure, starting from (±)-**6j**. Column chromatography: 5–15% ethyl acetate in hexane. This compound was obtained as a colourless semi-solid (91 mg, 78%). ^{1}H NMR (400 MHz, $CDCl_3$): δ 7.19-7.16 (m, 3H), 7.10-7.03 (m, 3H), 6.89 (t, *J* = 7.8 Hz, 1H), 6.07-5.99 (m, 1H), 5.09-5.04 (m, 2H), 3.96 (dd, *J* = 1.8, 11.0 Hz, 1H), 3.88 (s, 1H), 3.79 (d, *J* = 11.0 Hz, 1H), 3.40 (d, *J* = 6.4 Hz, 1H), 3.05-2.97 (m, 1H), 2.81-2.74 (m, 1H), 2.33 (br s, 3H), 2.14-2.07 (m, 1H), 1.82-1.76 (m, 1H). ^{13}C NMR (100 MHz, $CDCl_3$): δ 150.5, 137.3, 136.7, 136.6, 129.7, 129.2, 128.5, 128.1, 127.9, 126.6, 126.0, 120.8, 120.3, 115.4, 71.3, 67.9, 47.4, 34.2, 31.4, 26.3. Anal. Calcd. for $C_{20}H_{20}O_2$: C, 82.16; H, 6.89. Found: C, 82.19; H, 6.98.

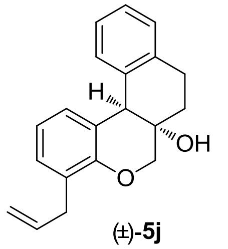

(±)-**5j**

(±)-*cis*-1,3-Dimethyl-6a,7,8,12b-tetrahydro-6*H*-naphtho[2,1-*c*]chromen-6a-ol (5k):
Compound (±)-**5k** was prepared according to the general procedure, starting from (±)-**6k**. Column chromatography: 5–15% ethyl acetate in hexane. This compound was obtained as a white solid (107 mg, 95%). M.p.: 161-162 °C. ^{1}H NMR (400 MHz, $CDCl_3$): δ 7.19-7.14 (m, 2H), 7.10-7.07 (m, 1H), 6.75 (s, 1H), 6.65 (s, 1H), 6.62 (d, *J* = 7.9 Hz, 1H), 3.80 (s, 1H), 3.71 (dd, *J* = 2.5, 11.0 Hz, 1H), 3.40 (d, *J* = 11.0 Hz, 1H), 3.12-3.07 (m, 1H), 2.81-2.76 (m, 1H), 2.50 (br s, 3H), 2.34-2.29 (m, 4H), 1.46-1.40 (m, 1H). ^{13}C NMR (100 MHz, $CDCl_3$): δ 153.0, 139.8, 138.3, 138.1, 138.0, 127.1, 126.9, 126.5, 126.3, 124.1, 115.5, 115.2, 69.9, 69.1, 44.3, 33.4, 27.1, 21.0, 18.8. Anal. Calcd. for $C_{19}H_{20}O_2$: C, 81.40; H, 7.19. Found: C, 81.36; H, 7.26.

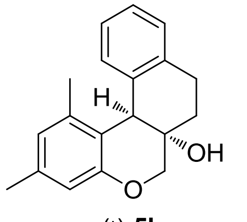

(±)-**5k**

(±)-*cis*-2-Bromo-6a,7,8,12b-tetrahydro-6*H*-naphtho[2,1-*c*]chromen-6a-ol (5l):

Compound (±)-**5l** was prepared according to the general procedure, starting from (±)-**6l**. Column chromatography: 5–15% ethyl acetate in hexane. This compound was obtained as a white solid (99 mg, 75%). M.p.: 152-153 °C. ^{1}H NMR (400 MHz, $CDCl_3$): δ 7.28-7.16 (m, 5H), 7.9-7.06 (m, 1H), 6.67 (d, *J* = 8.2 Hz, 1H), 3.93 (dd, *J* = 1.4, 11.0 Hz, 1H), 3.84 (s, 1H), 3.78 (d, *J* = 11.0 Hz, 1H), 3.03-2.95 (m, 1H), 2.80-2.73 (m, 1H), 2.16 (br s, 3H), 2.09-2.02 (m, 1H), 1.83-1.77 (m, 1H). ^{13}C NMR (100 MHz, $CDCl_3$): δ 152.0, 136.3, 136.0, 133.7, 131.2, 129.4, 128.3, 127.1, 126.3, 123.8, 118.7, 112.9, 71.5, 67.6, 47.1, 31.2, 26.1. Anal. Calcd. for $C_{17}H_{15}BrO_2$: C, 61.65; H, 4.56. Found: C, 61.72; H, 4.59.

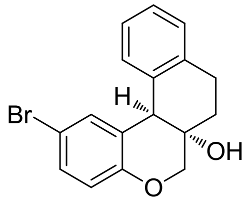

(±)-**5l**

(±)-*cis*-6a,7,8,12b-Tetrahydro-6*H*-benzo[h]naphtho[2,1-*c*]chromen-6a-ol (5m):

Compound (±)-**5m** was prepared according to the general procedure, starting from (±)-**6m**. Column chromatography: 5–15% ethyl acetate in hexane. This compound was obtained as a white solid (107 mg, 89%). M.p.: 152-153 °C. ^{1}H NMR (400 MHz, $CDCl_3$): δ 8.23-8.21 (m, 1H), 7.80-7.78 (m, 1H), 7.50-7.43 (m, 3H), 7.27-7.24 (m, 1H), 7.18-7.11 (m, 3H), 7.06 (d, *J* = 7.6 Hz, 1H), 4.09 (dd, *J* = 1.8, 11.0 Hz, 1H), 3.96 (s, 1H), 3.84 (d, *J* = 11.0 Hz, 1H), 3.07-3.01 (m, 1H), 2.81-2.77 (m, 1H), 2.24-2.20 (m, 2H), 1.76-1.71 (m, 1H). ^{13}C NMR (100 MHz, $CDCl_3$): δ 148.2, 138.0, 137.3, 133.7, 129.2, 128.8, 127.7, 127.4, 126.7, 126.4, 126.2, 125.5, 124.9, 121.9, 120.3, 114.4, 71.3, 68.3, 47.3, 32.2, 26.7. Anal. Calcd. for $C_{21}H_{18}O_2$: C, 83.42; H, 6.00. Found: C, 83.36; H, 6.09.

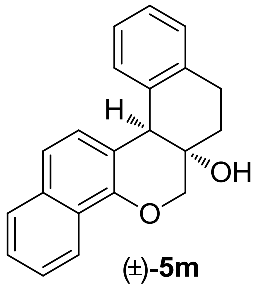

(±)-**5m**

(±)-*cis*-4a,5,6,10b-Tetrahydro-4*H*-benzo[*f*]naphtho[2,1-*c*]chromen-4a-ol (5n):

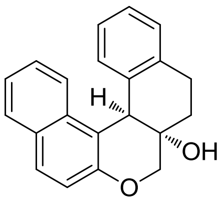

(±)-**5n**

Compound (±)-**5n** was prepared according to the general procedure, starting from (±)-**6n**. Column chromatography: 5–15% ethyl acetate in hexane. This compound was obtained as a white solid (102 mg, 85%). M.p.: 152-153 °C. ^{1}H NMR (400 MHz, $CDCl_3$): δ 7.85-7.83 (m, 1H), 7.80 (d, *J* = 8.8 Hz, 1H), 7.63 (d, *J* = 8.0 Hz, 1H), 7.42-7.36 (m, 2H), 7.23-7.15 (m, 3H), 6.97 (t, *J* = 7.6 Hz, 1H), 6.58 (d, *J* = 7.6 Hz, 1H), 4.34 (s, 1H), 3.88 (dd, *J* =

2.4, 11.0 Hz, 1H), 3.62 (d, J = 11.0 Hz, 1H), 3.24-3.18 (m, 1H), 2.90-2.85 (m, 1H), 2.53 (br s, 1H), 2.43-2.39 (m, 1H), 1.56-1.50 (m, 1H). ^{13}C NMR (100 MHz, $CDCl_3$): δ 151.0, 138.2, 138.1, 134.4, 129.5, 129.4, 128.5, 128.3, 126.9, 126.8, 126.6, 126.2, 123.6, 123.0, 118.7, 111.8, 70.2, 68.7, 47.3, 33.6, 27.2. Anal. Calcd. for $C_{21}H_{18}O_2$: C, 83.42; H, 6.00. Found: C, 83.50; H, 6.04.

4. X-ray crystallography

Experimental

X-ray crystallography: X-ray reflections were collected on a Bruker APEX-II, CCD diffractometer using Mo Kα (λ = 0.71073 Å) radiation. Data reduction was performed using Bruker SAINT Software [2a]. Intensities for absorption were corrected using SADABS. Structures were solved and refined using SHELXL-2014 with anisotropic displacement parameters for non-H atoms. Hydrogen atom on O was experimentally located in the crystal structure. All C–H atoms were fixed geometrically using the HFIX command in SHELX-TL [2b]. A check of the final CIF file using PLATON did not show any missed symmetry [2c,d]. The crystallographic parameters for all structures are summarized in Table S1.

Compound (±)-**5k** is crystallized in the monoclinic space group $P2_1/n$ with 1 symmetry independent molecule in the crystal lattice. The ORTEP diagram of **5k** is shown below.

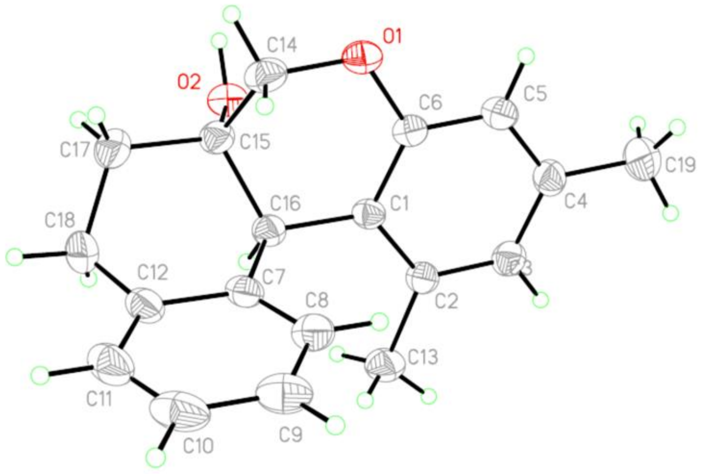

Figure S1. ORTEP diagram of **5k** with 35% probability ellipsoid.

Crystal data are summarized in Table S1. Two such inversely related molecules form O−H···O hydrogen bonded homodimer. The C−H···π and weak C−H···O interactions are the major contributor to complete the molecular packing of the crystal.

Table S1. Crystal data parameters.

Crystal Data	
Formula unit	$C_{19}H_{20}O_2$
Formula wt.	280.35
Crystal system	Monoclinic
T [K]	100
a [Å]	9.1569(9)
b [Å]	7.0032(6)
c [Å]	23.2281(18)
α [°]	90
β [°]	95.727(18)
γ [°]	90
Volume [Å^3]	1482.1(2)
Space group	$P2_1/n$
Z	4
D_{calc} [g cm^{-3}]	1.256
μ/mm^{-1}	0.080
Reflns. Collected	4566
Unique reflns.	1689
Observed reflns.	1330
R_1 [I>2σ(I)], wR_2	0.0428; 0.1161
GOF	1.004
Instrument	Bruker APEX-II
X-ray	MoK\α; λ=0.71073
CCDC Reference No.	1485292

5. Preparation of (±)-*cis*-1,3-dimethyl-6a,7,8,12b-tetrahydro-6*H*-naphtho[2,1-*c*]chromene (14):

$BF_3 \cdot Et_2O$ (0.25 mL, 1.50 mmol) was added drop-wise to a solution of (±)-**5k** (200 mg, 0. 71 mmol) and triethylsilane (0.19 ml, 1.5 mmol) in anhydrous CH_2Cl_2 (222 mL) at 0 °C under nitrogen atmosphere and then stirred for 2 h at rt. The reaction mixture was cooled on an ice bath, then quenched with aq. saturated $NaHCO_3$ solution (10 mL). The resulting mixture extracted with CH_2Cl_2 (2 × 15 mL). The combined organic layers were washed with brine (10 mL), dried over Na_2SO_4, filtered and concentrated under reduced pressure. The crude product was purified by column chromatography (5% EtOAc in hexane) to afford (±)-**14** (185 mg, 98%) as a colorless gum. ^1H NMR (400 MHz, $CDCl_3$): δ 7.27-7.16 (m, 3H), 7.06 (d, *J* = 6.4 Hz, 1H), 6.60 (d, *J* = 8.7 Hz, 2H), 3.97 (d, *J* = 10.5 Hz, 1H), 3.91 (dd, *J* = 10.2, 2.3 Hz, 1H), 2.99 (dd, *J* = 10.2, 2.3 Hz, 1H), 2.88 (d, *J* = 16.5 Hz, 1H), 2.64 (d, *J* = 16.5 Hz, 1H), 2.27-2.21 (m, 4H), 2.15 (s, 3H), 1.91-1.84 (m, 1H). ^{13}C NMR (100 MHz, $CDCl_3$): δ 154.1, 146.8, 143.9, 137.6, 136.6, 127.4, 126.7, 124.8, 123.2, 123.0, 117.4, 114.6, 71.7, 45.7, 35.4, 35.0, 29.9, 20.9, 19.0. Anal. Calcd. for $C_{19}H_{20}O$: C, 86.32; H, 7.63. Found: C, 86.25; H, 7.71.

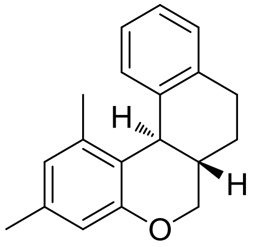

(±)-**14**

6. References

1. T. Miyashi, Y. Nishizawa, Y. Fujii, K. Yamakawa, M. Kamata, S. Akao and T. Mukai, *J. Am. Chem. Soc.* 1986, **108**,1617-1632.
2. (a) SAINT Plus, Bruker AXS Inc.: Madison, WI, 2008; BRUKER AXS (v 6.14); (b) Bruker AXS Inc.: Madison, WI, 2008; (c) PLATON, A Multipurpose Crystallographic Tool; A. L. Spek, Utrecht University: Utrecht, Netherland, 2002; (d) A. L. Spek, *J. Appl. Crystallogr.*, 2003, **36**, 7–13.

7. Copies of NMR Spectra of final compounds

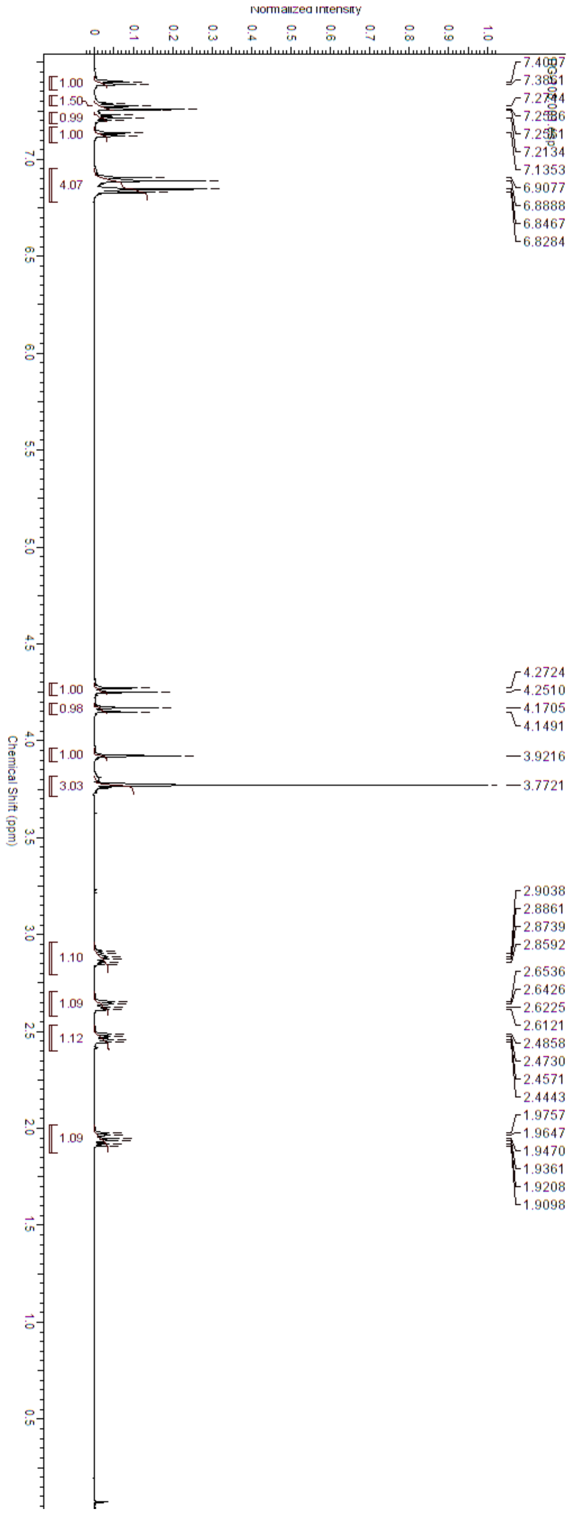

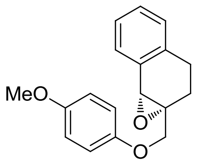

(±)-**6a**

^{1}H NMR (400 MHz, $CDCl_3$) spectrum of compound (±)-**6a**.

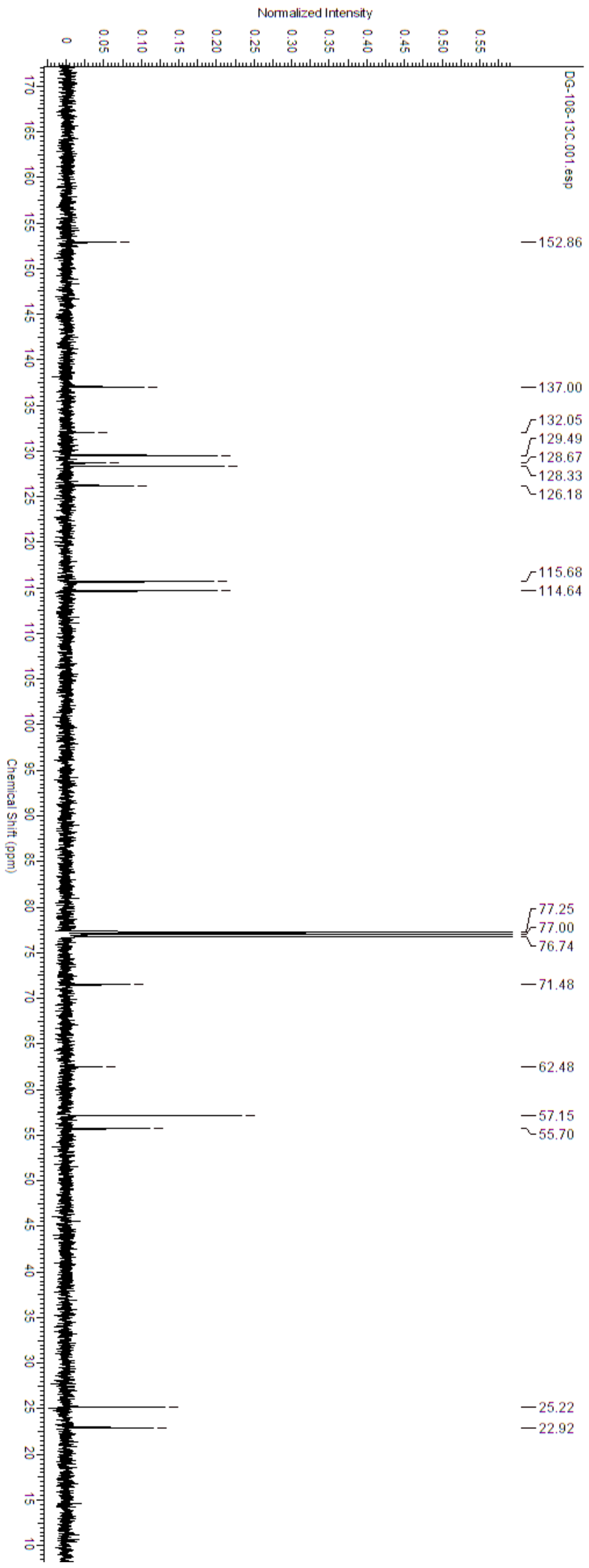

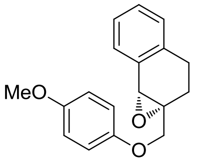

(±)-**6a**

^{13}C NMR (100 MHz, $CDCl_3$) spectrum of compound (±)-**6a**.

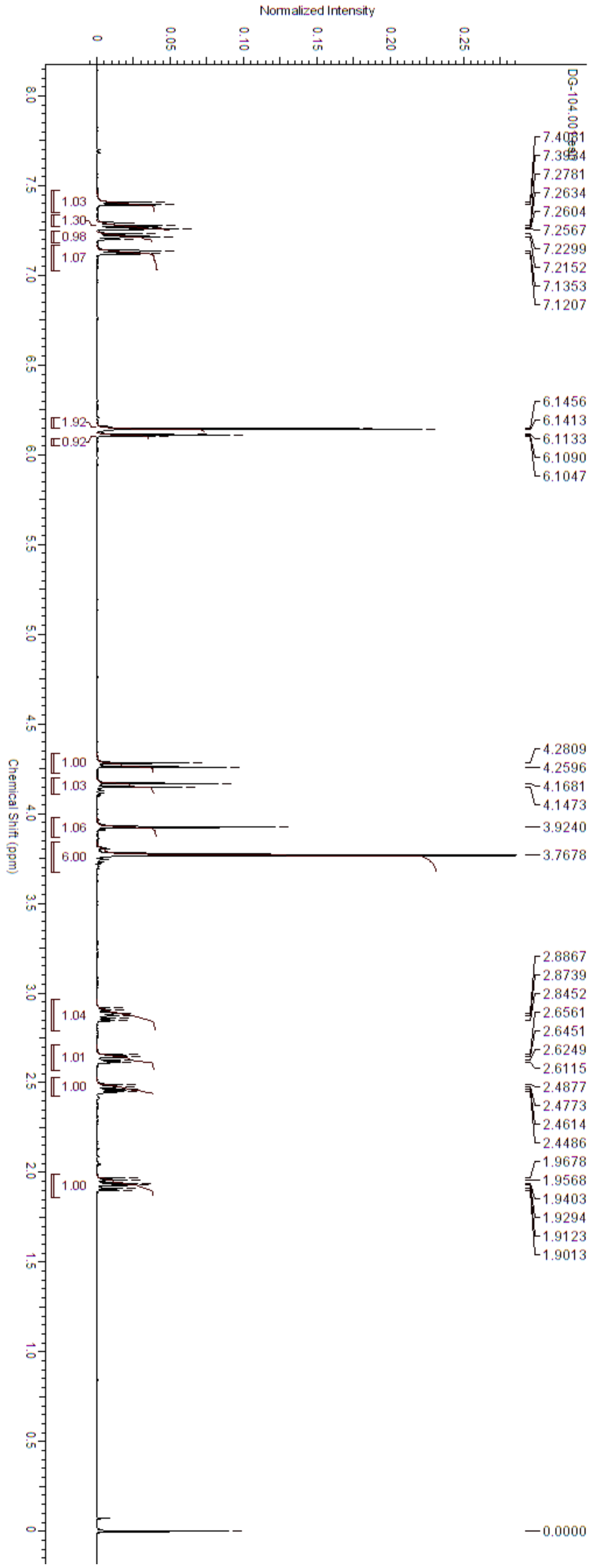

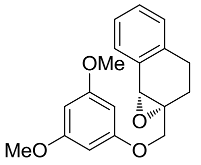

(±)-**6b**

^{1}H NMR (400 MHz, $CDCl_3$) spectrum of compound (±)-**6b**.

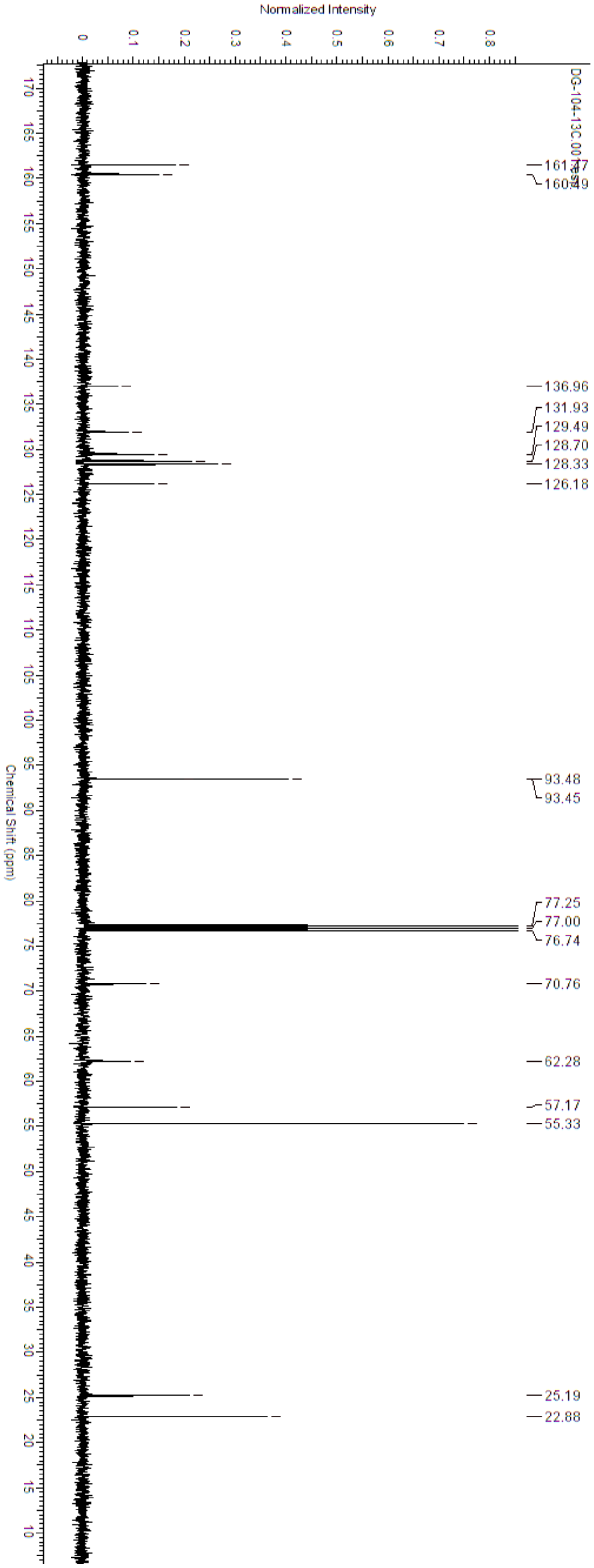

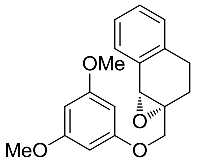

(±)-**6b**

^{13}C NMR (100 MHz, $CDCl_3$) spectrum of compound (±)-**6b**.

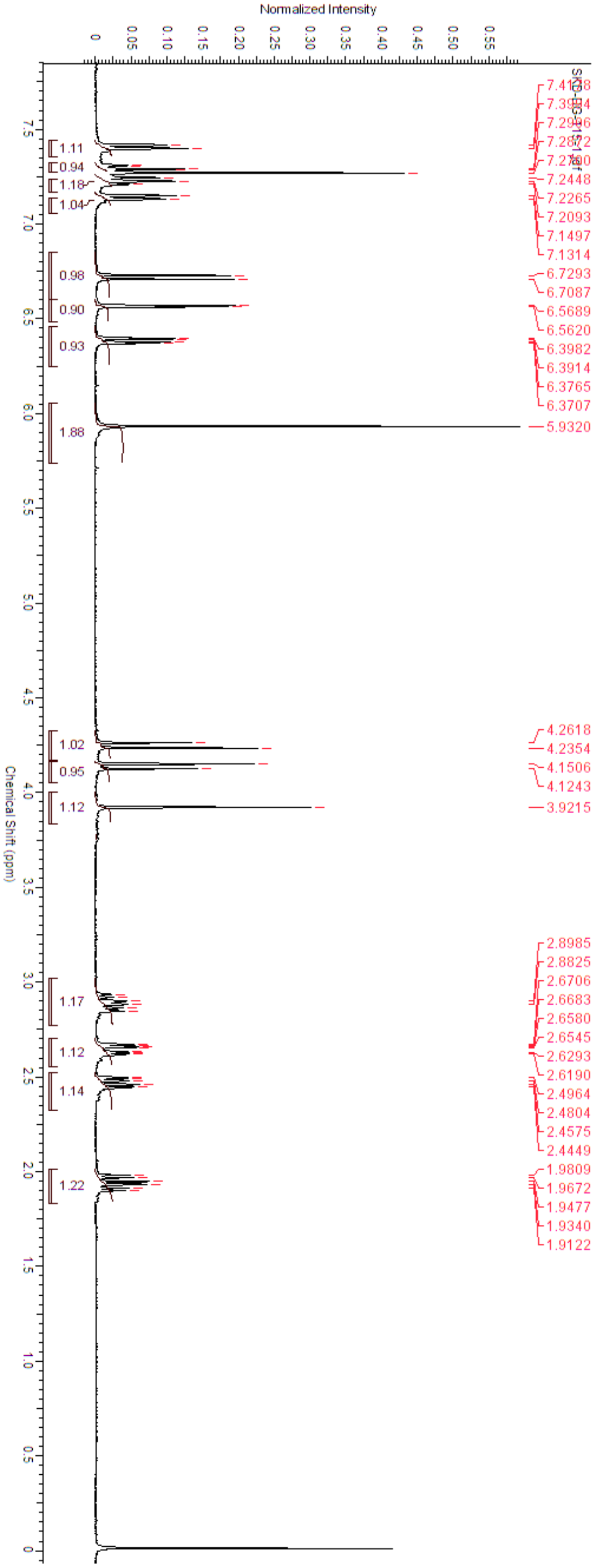

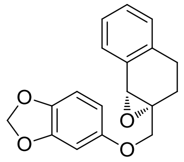

(±)-**6c**

^{1}H NMR (400 MHz, $CDCl_3$) spectrum of compound (±)-**6c**.

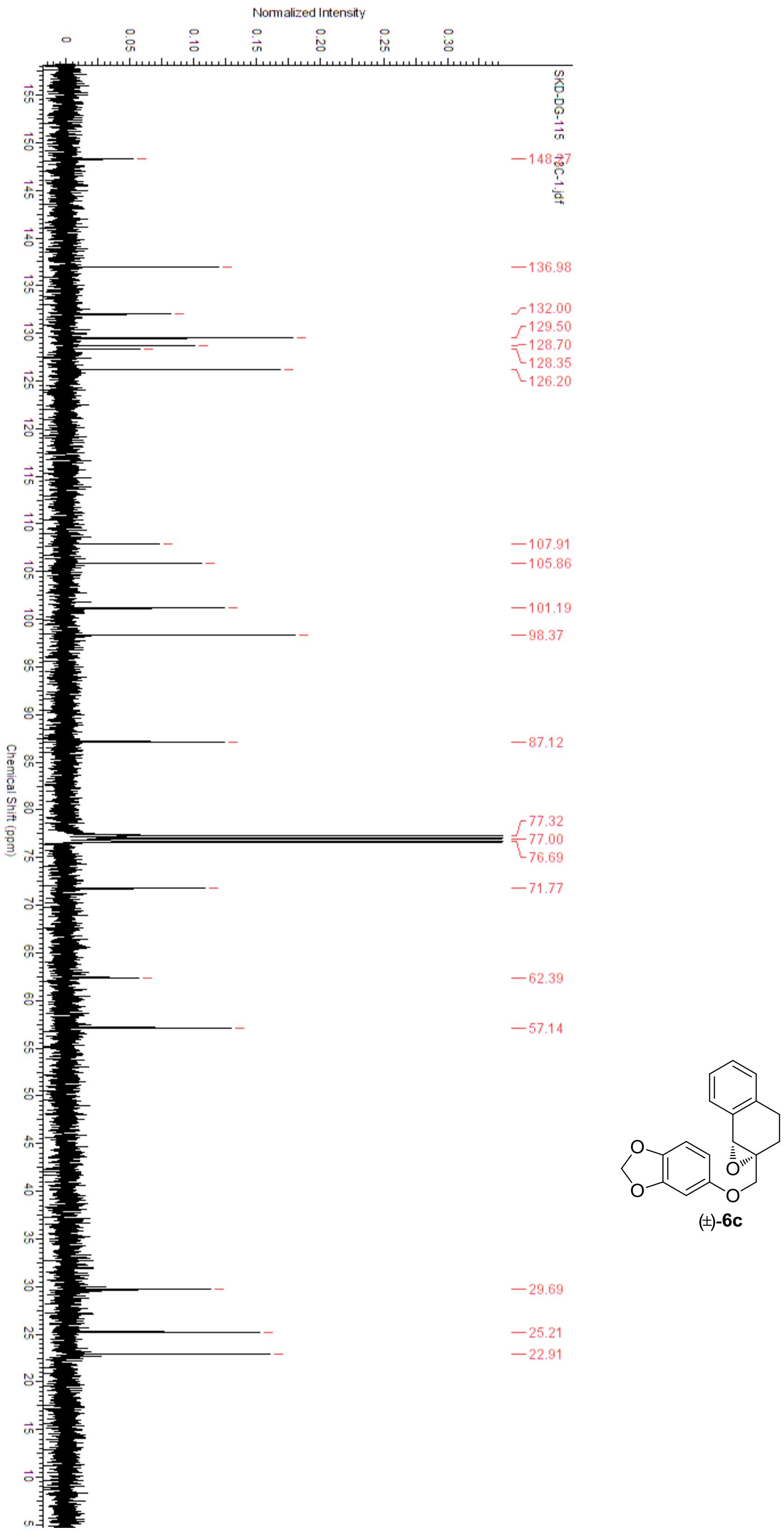

(±)-**6c**

^{13}C NMR (100 MHz, $CDCl_3$) spectrum of compound (±)-**6c**.

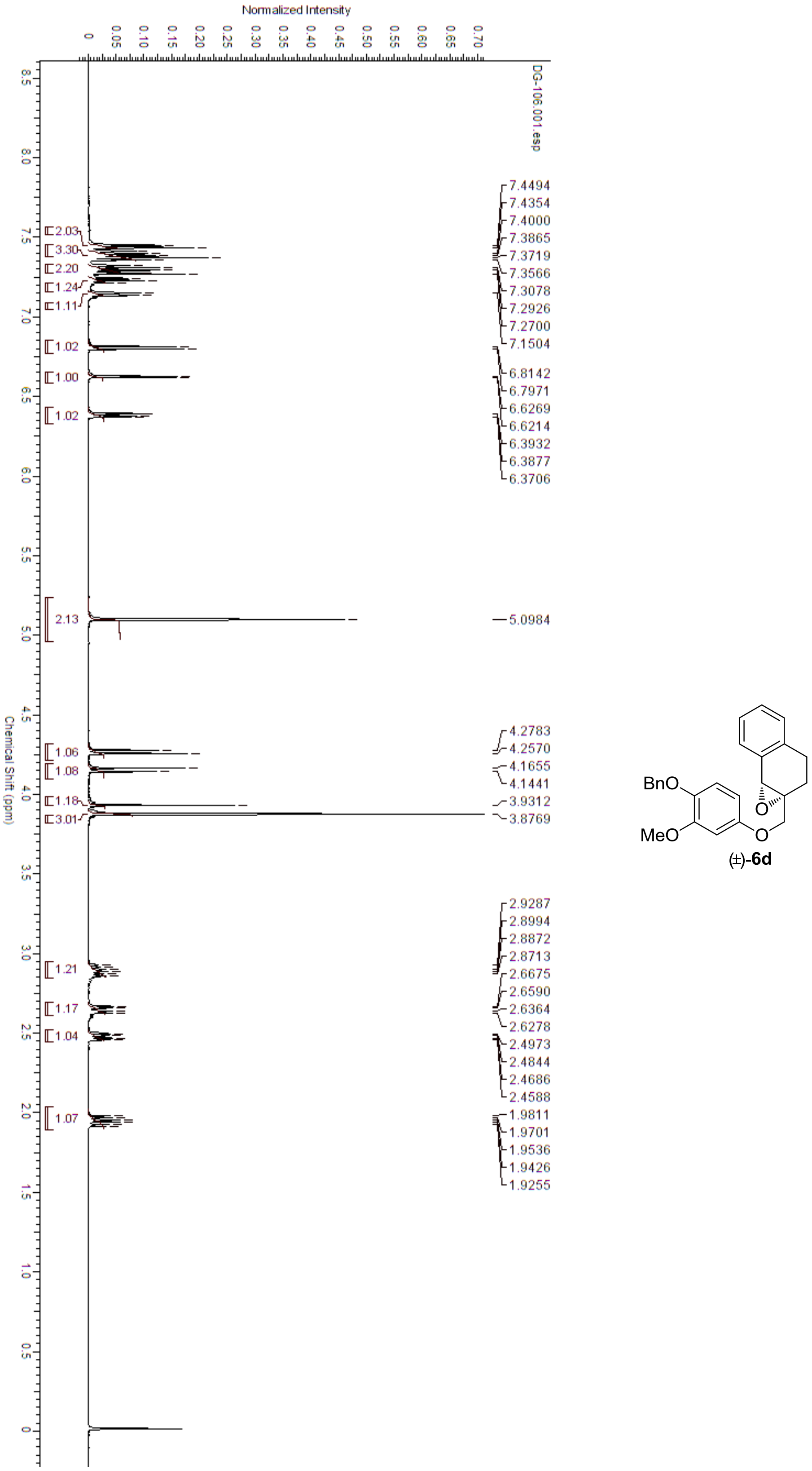

^{1}H NMR (400 MHz, $CDCl_3$) spectrum of compound (±)-**6d**

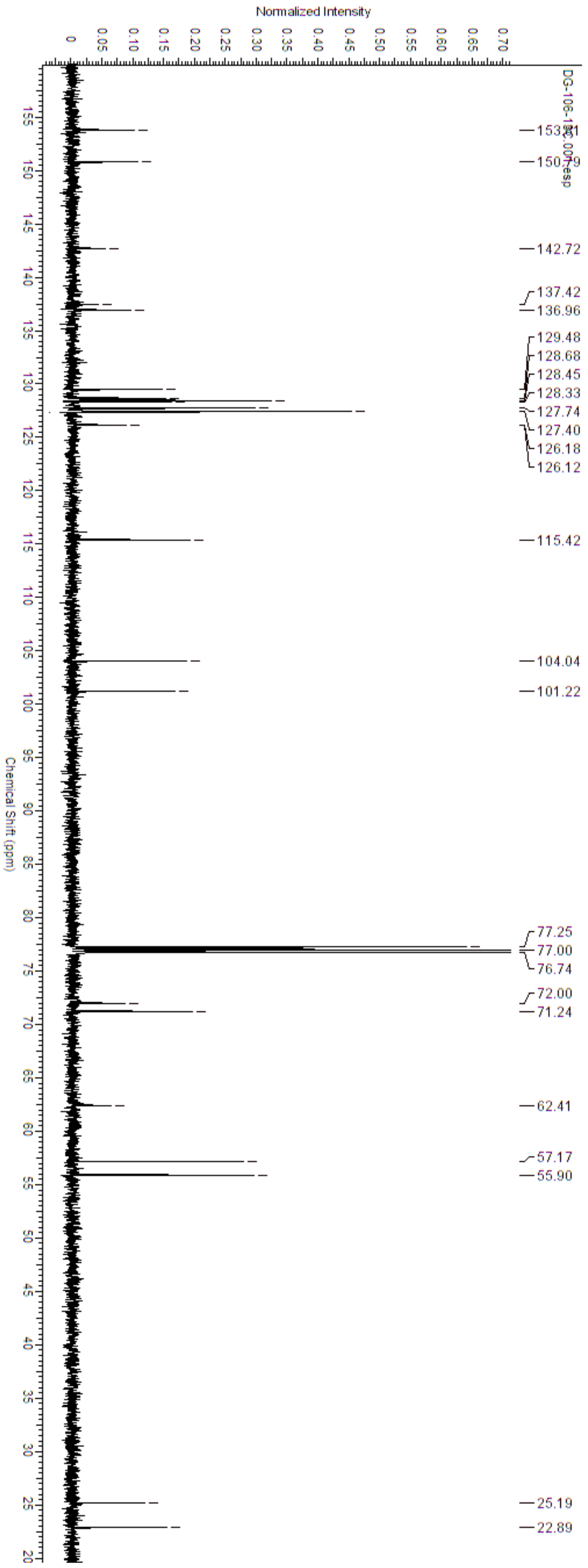

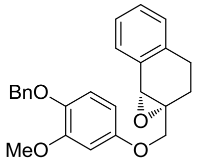

(±)-**6d**

^{13}C NMR (100 MHz, $CDCl_3$) spectrum of compound (±)-**6d**.

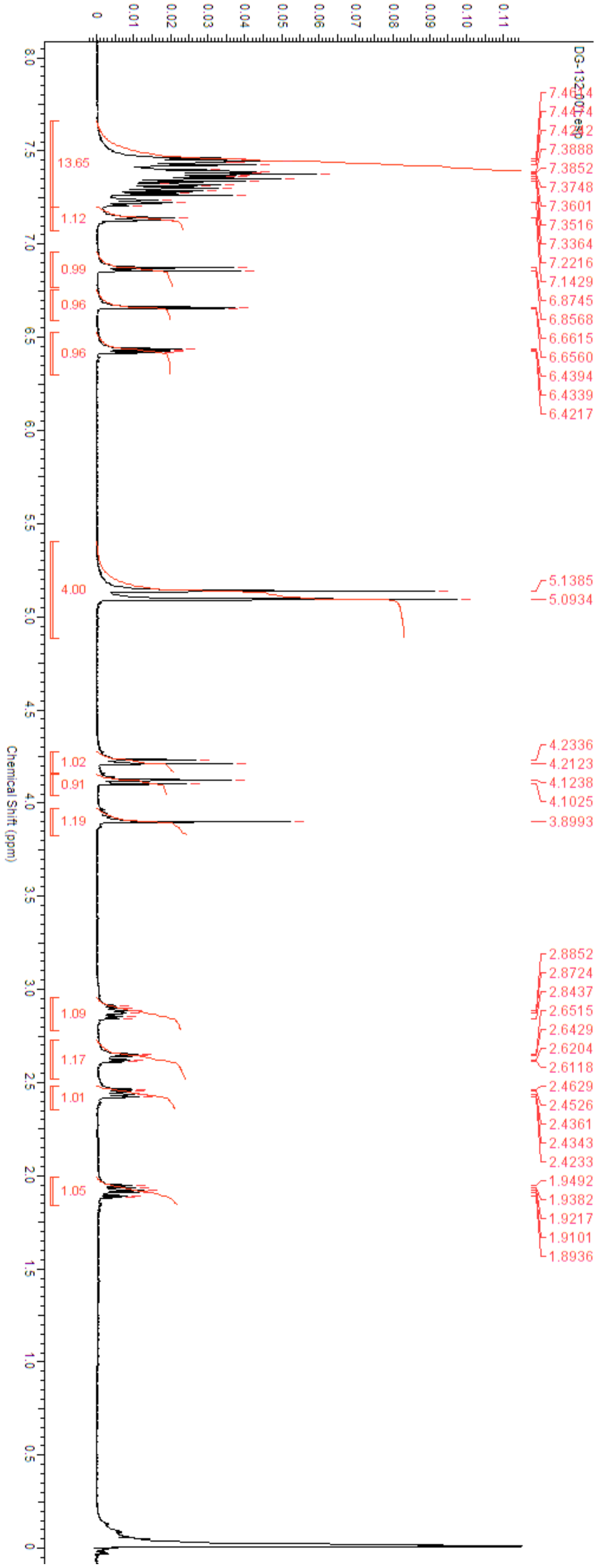

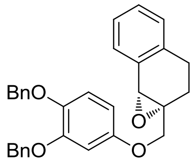

(±)-**6e**

^{1}H NMR (400 MHz, $CDCl_3$) spectrum of compound (±)-**6e**

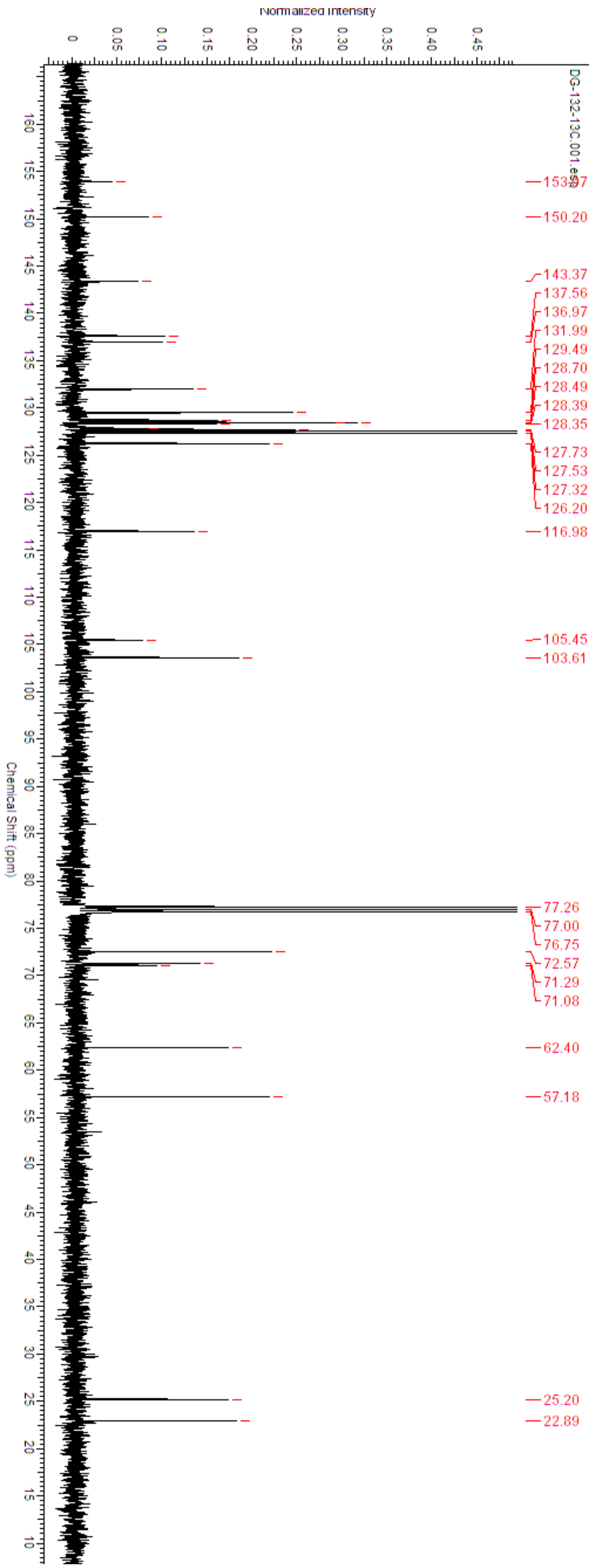

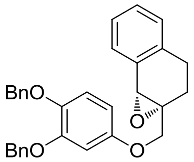

(±)-**6e**

^{13}C NMR (100 MHz, $CDCl_3$) spectrum of compound (±)-**6e**.

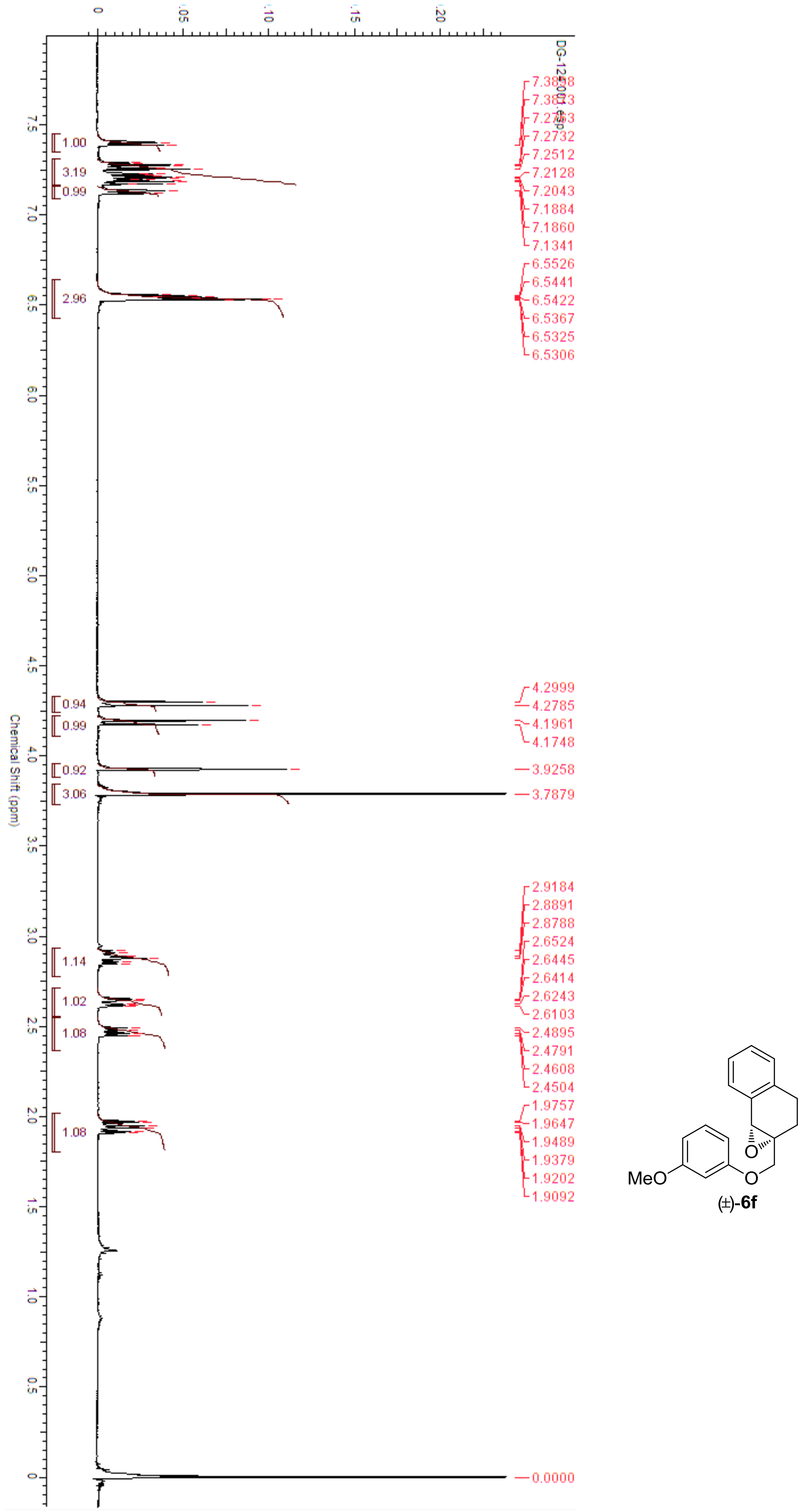

^{1}H NMR (400 MHz, $CDCl_3$) spectrum of compound (±)-**6f**

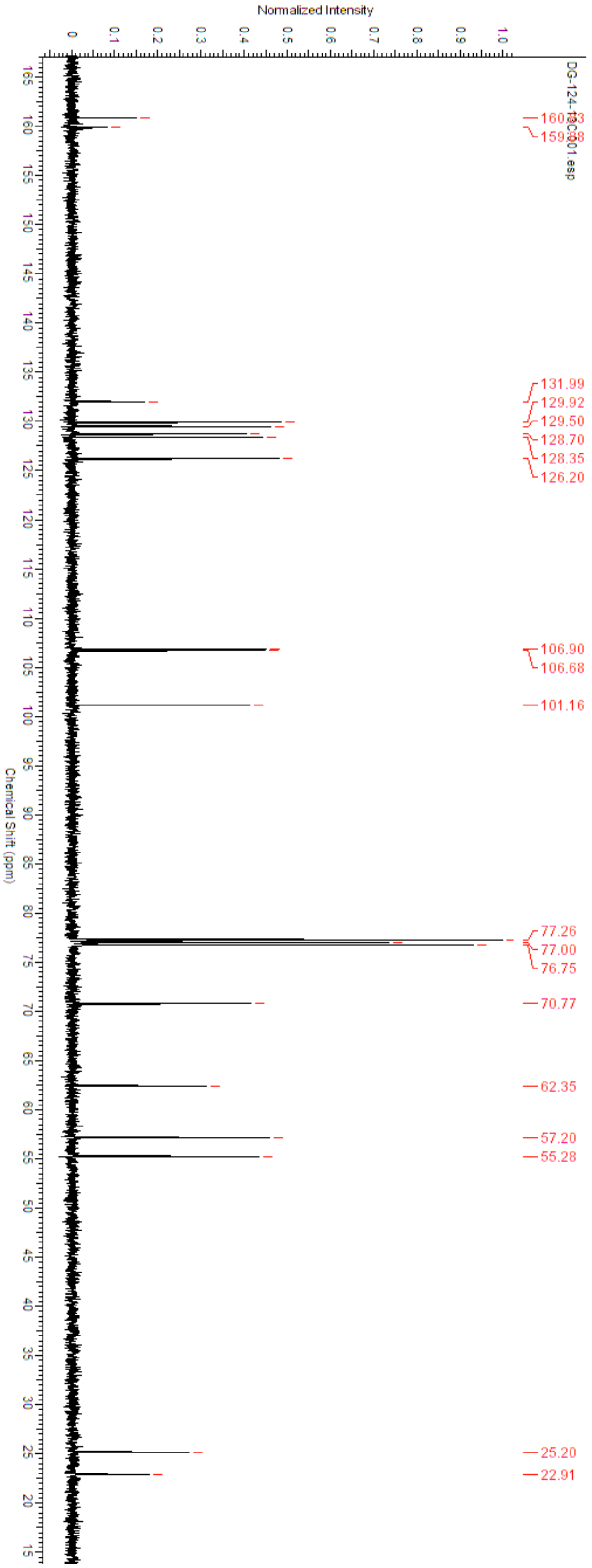

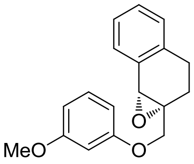

(±)-**6f**

^{13}C NMR (100 MHz, $CDCl_3$) spectrum of compound (±)-**6f**.

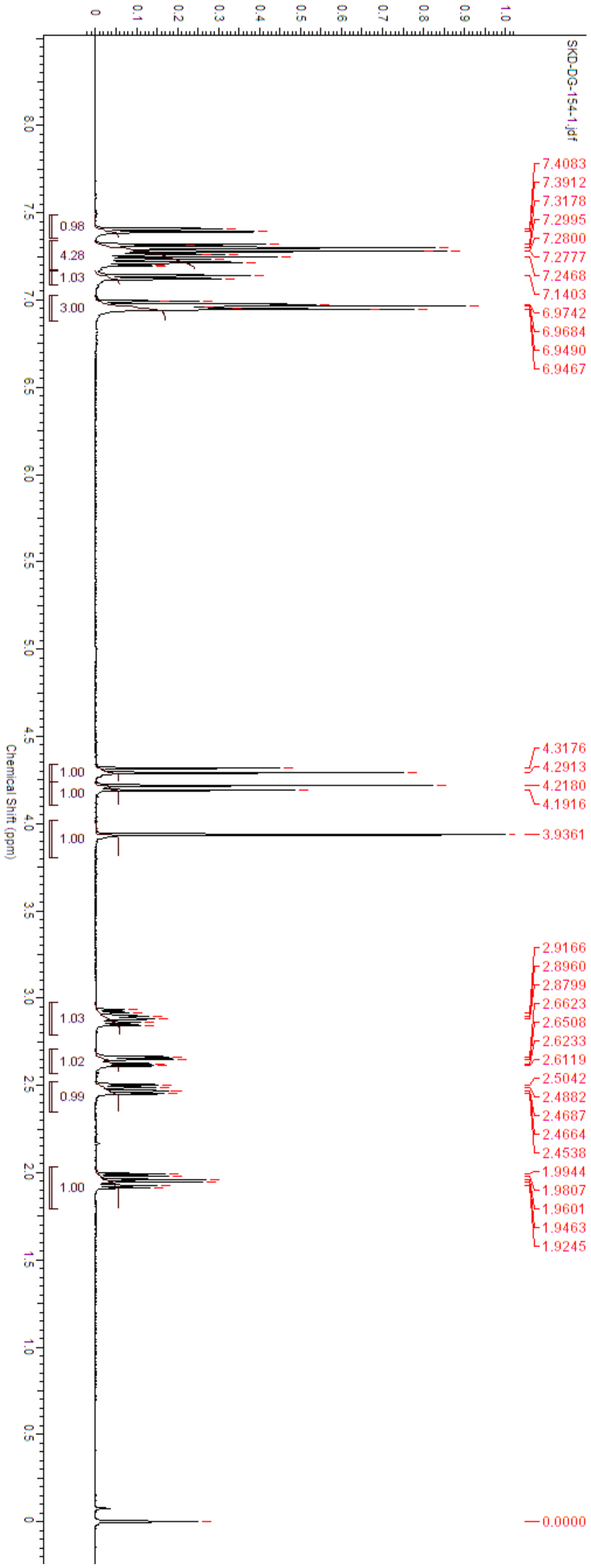

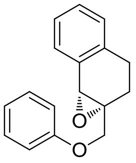

(±)-**6g**

^{1}H NMR (400 MHz, $CDCl_3$) spectrum of compound (±)-**6g**.

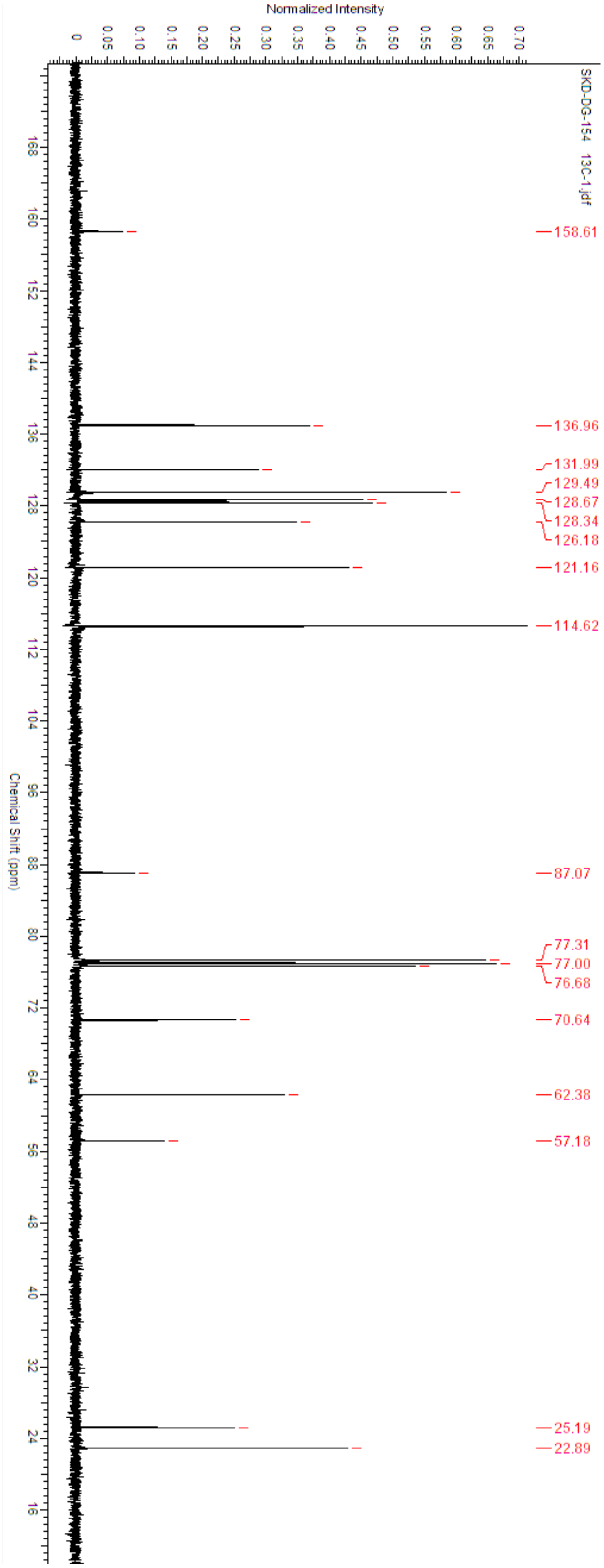

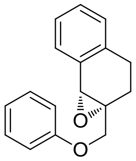

(±)-**6g**

^{13}C NMR (100 MHz, $CDCl_3$) spectrum of compound (±)-**6g**.

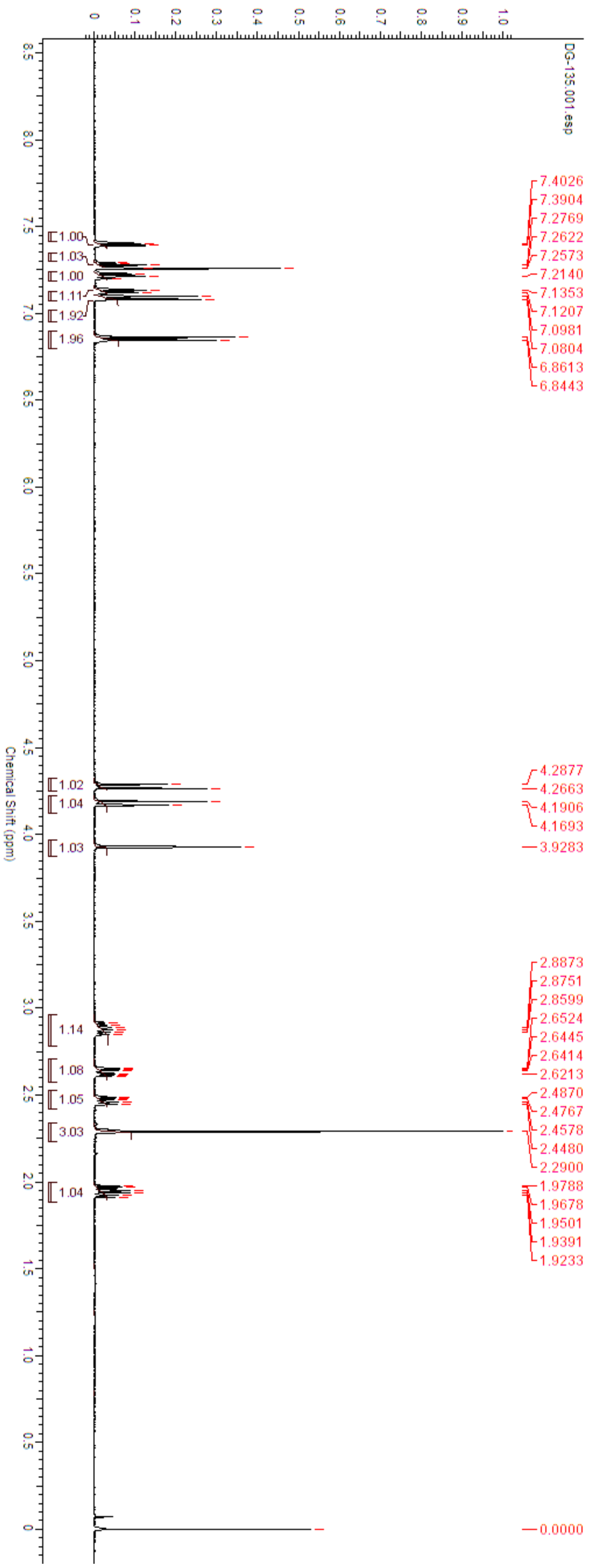

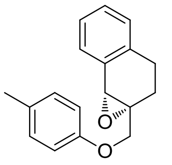

(±)-**6h**

^{1}H NMR (400 MHz, $CDCl_3$) spectrum of compound (±)-**6h**.

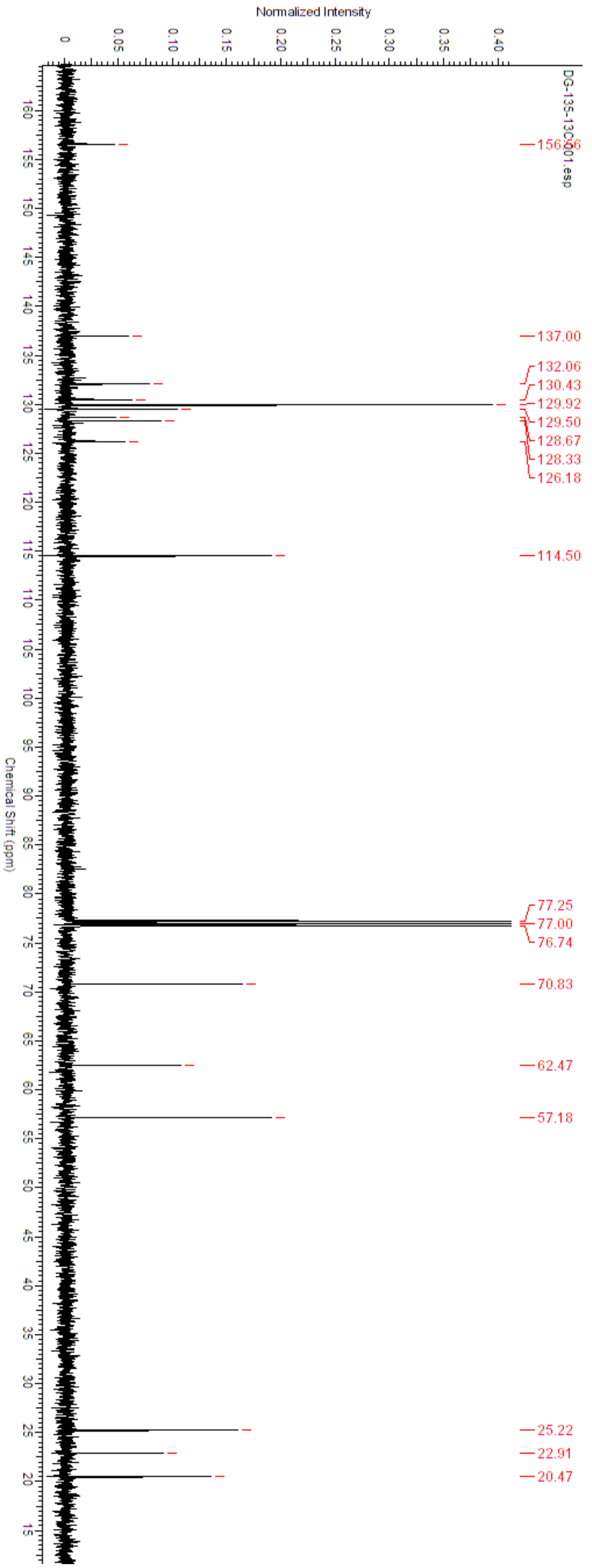

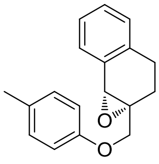

(±)-**6h**

^{13}C NMR (100 MHz, $CDCl_3$) spectrum of compound (±)-**6h**.

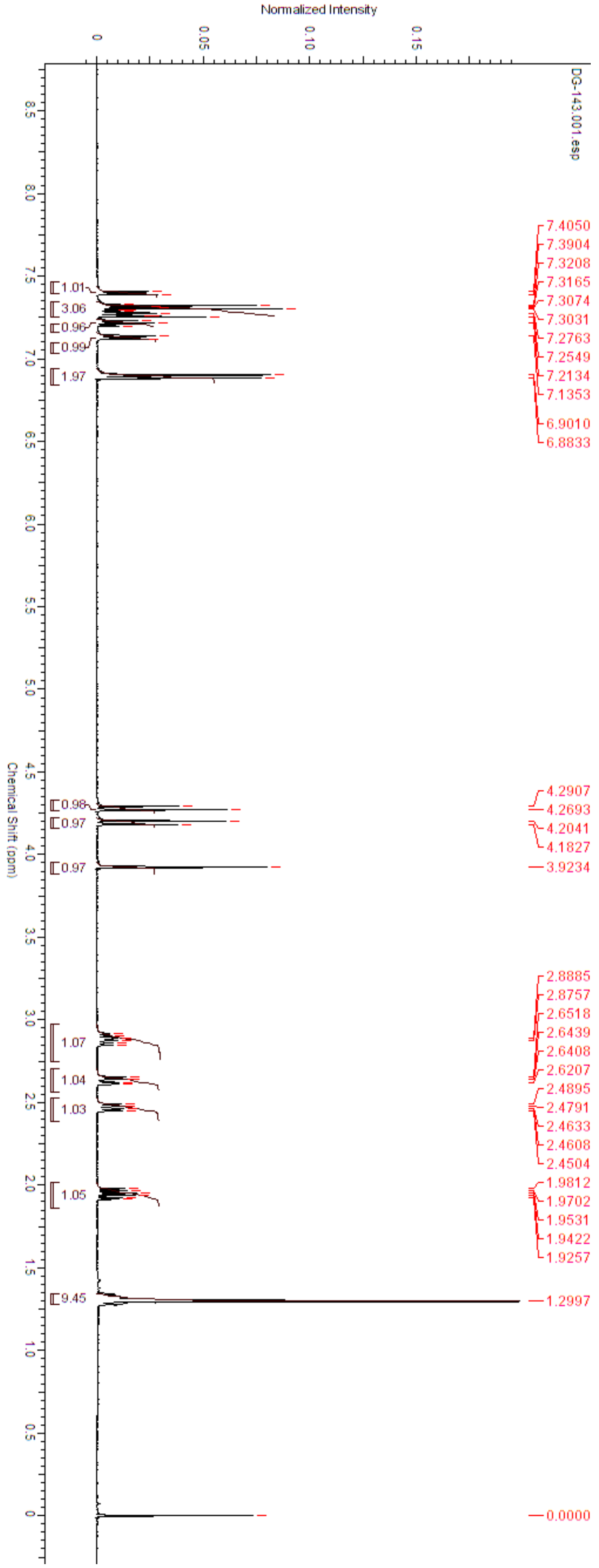

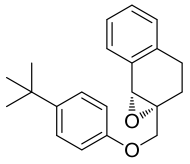

(±)-**6i**

^{1}H NMR (400 MHz, $CDCl_3$) spectrum of compound (±)-**6i**.

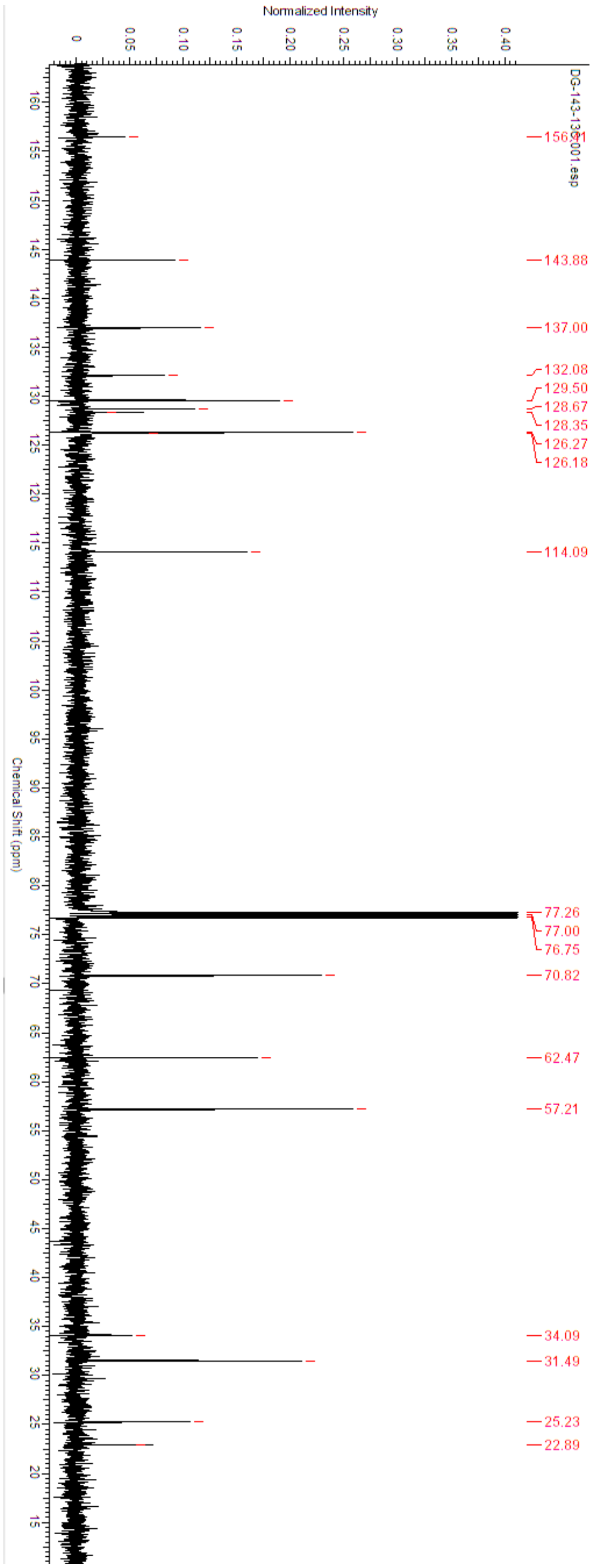

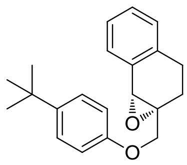

(±)-**6i**

^{13}C NMR (100 MHz, $CDCl_3$) spectrum of compound (±)-**6i**.

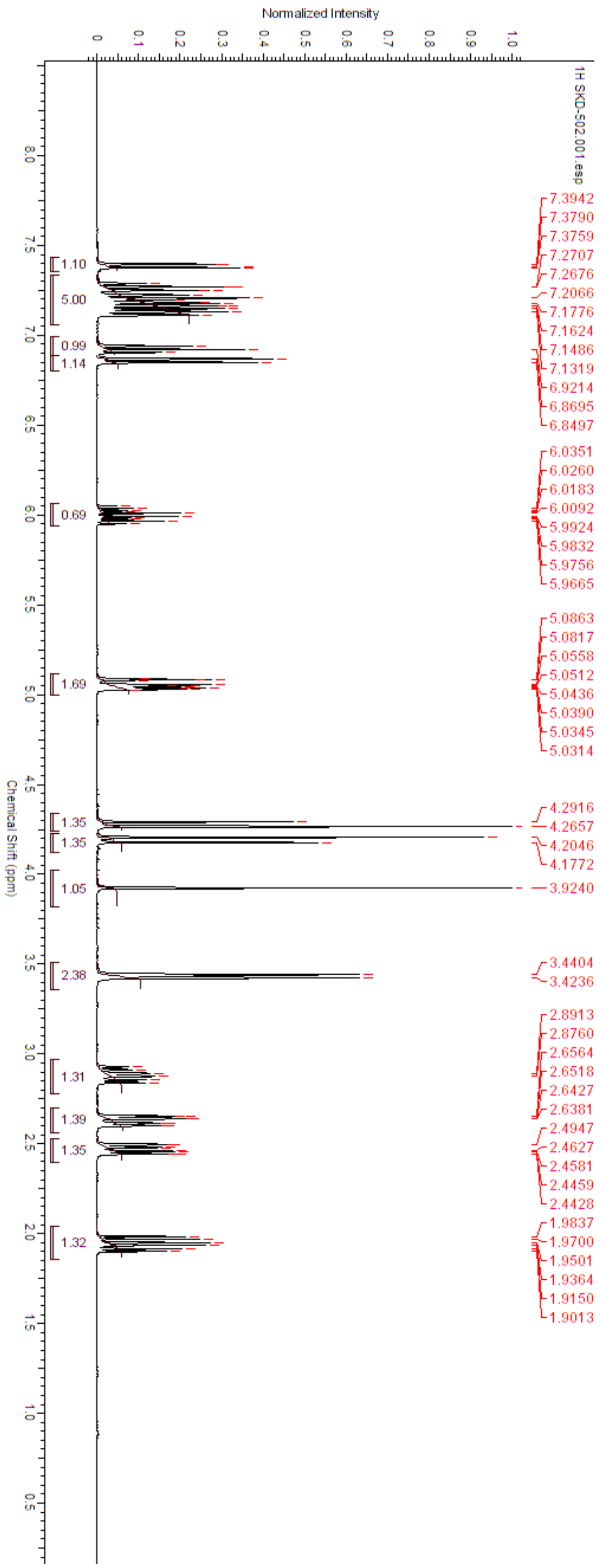

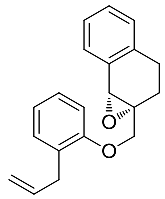

(±)-**6j**

^{1}H NMR (400 MHz, $CDCl_3$) spectrum of compound (±)-**6j**.

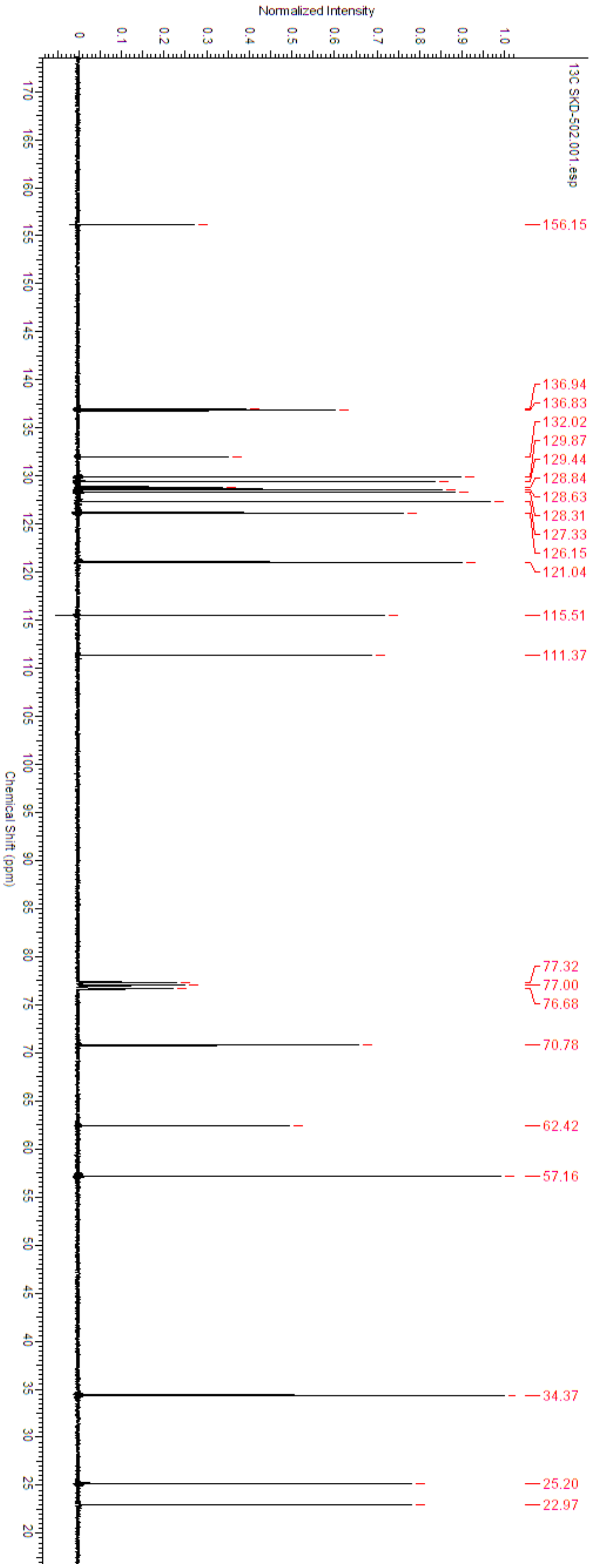

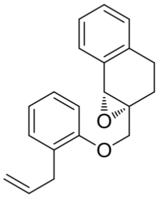

(±)-**6j**

^{13}C NMR (100 MHz, $CDCl_3$) spectrum of compound (±)-**6j**.

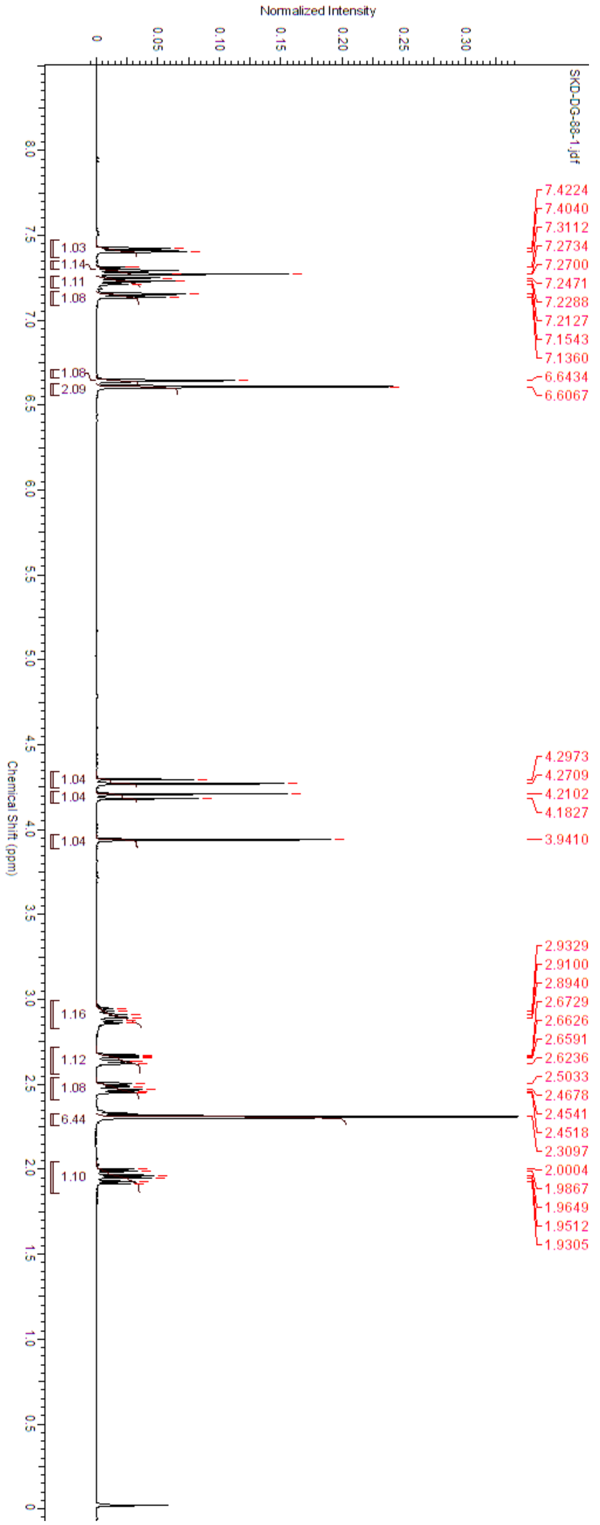

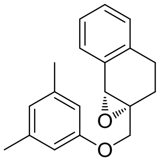

(±)-**6k**

^{1}H NMR (400 MHz, $CDCl_3$) spectrum of compound (±)-**6k**.

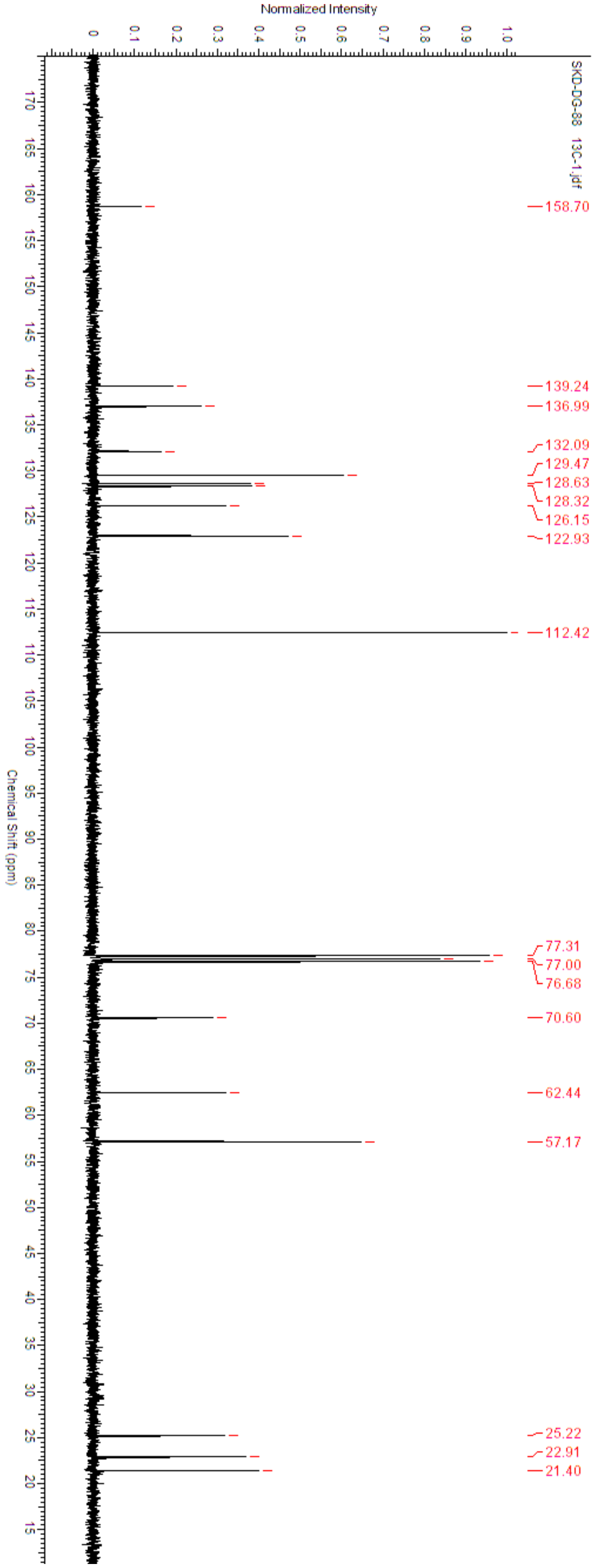

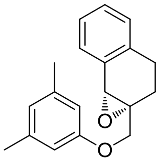

(±)-**6k**

^{13}C NMR (100 MHz, $CDCl_3$) spectrum of compound (±)-**6k**.

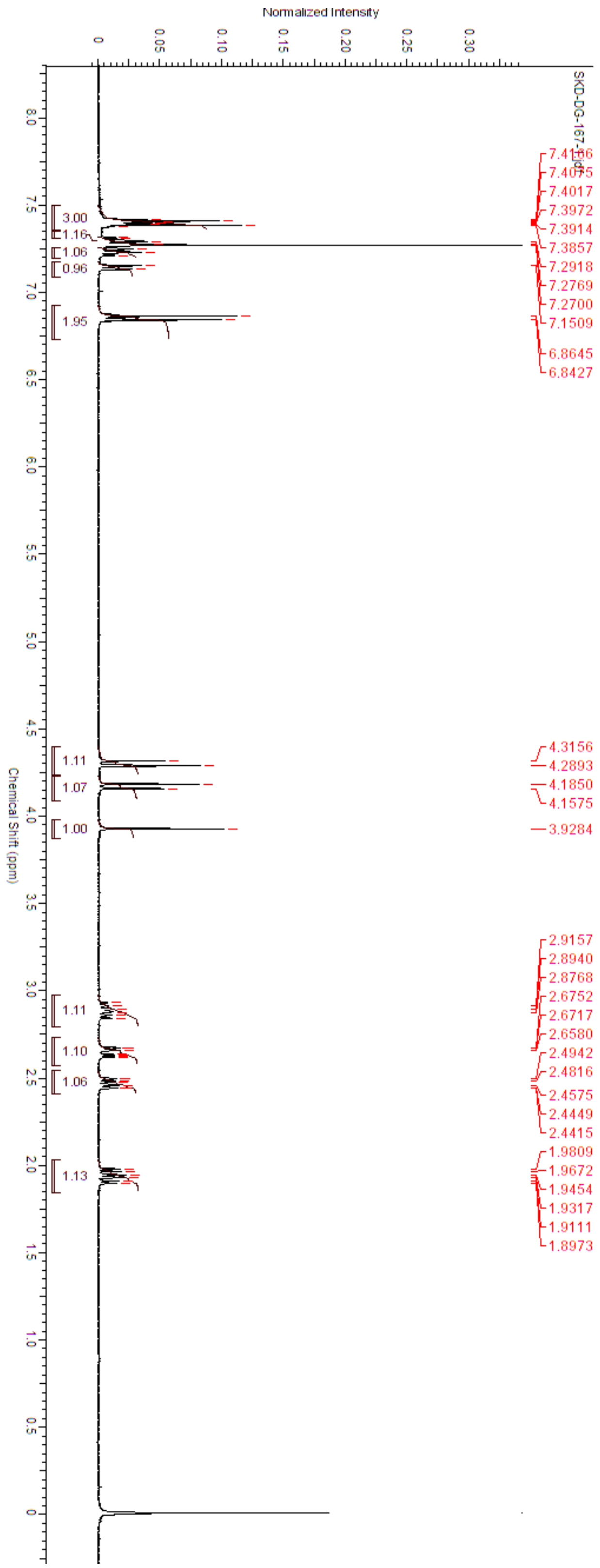

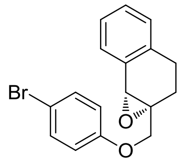

(±)-**6l**

^{1}H NMR (400 MHz, $CDCl_3$) spectrum of compound (±)-**6l**.

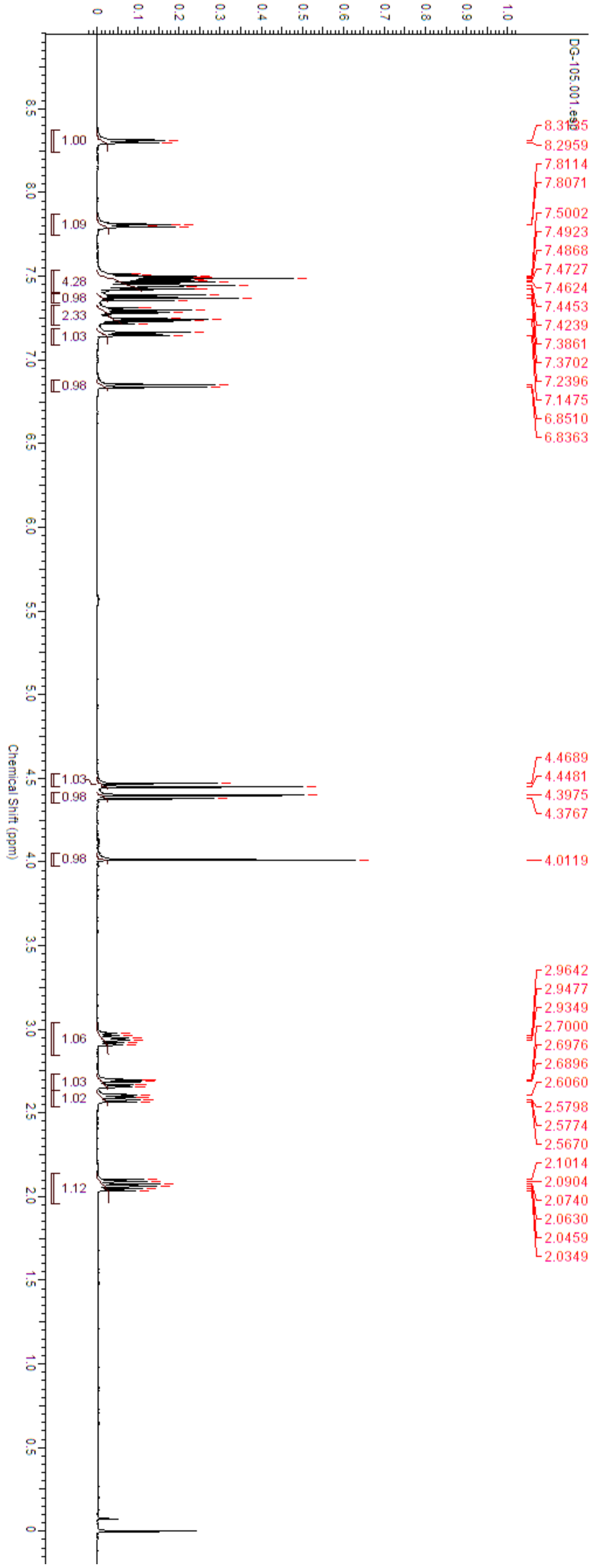

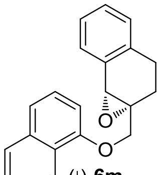
(±)-**6m**

^{1}H NMR (400 MHz, $CDCl_3$) spectrum of compound (±)-**6m**.

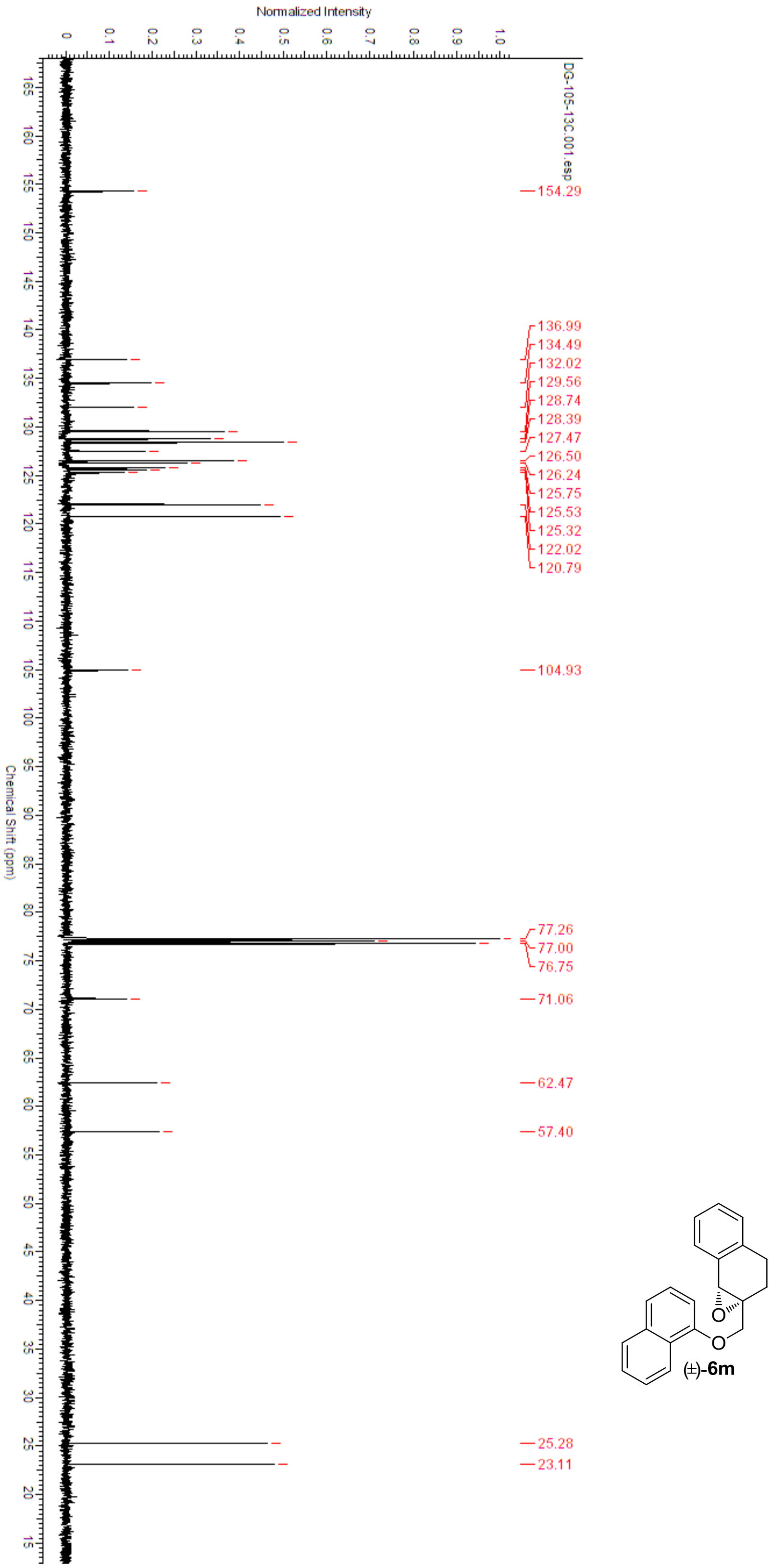

^{13}C NMR (100 MHz, $CDCl_3$) spectrum of compound (±)-**6m**.

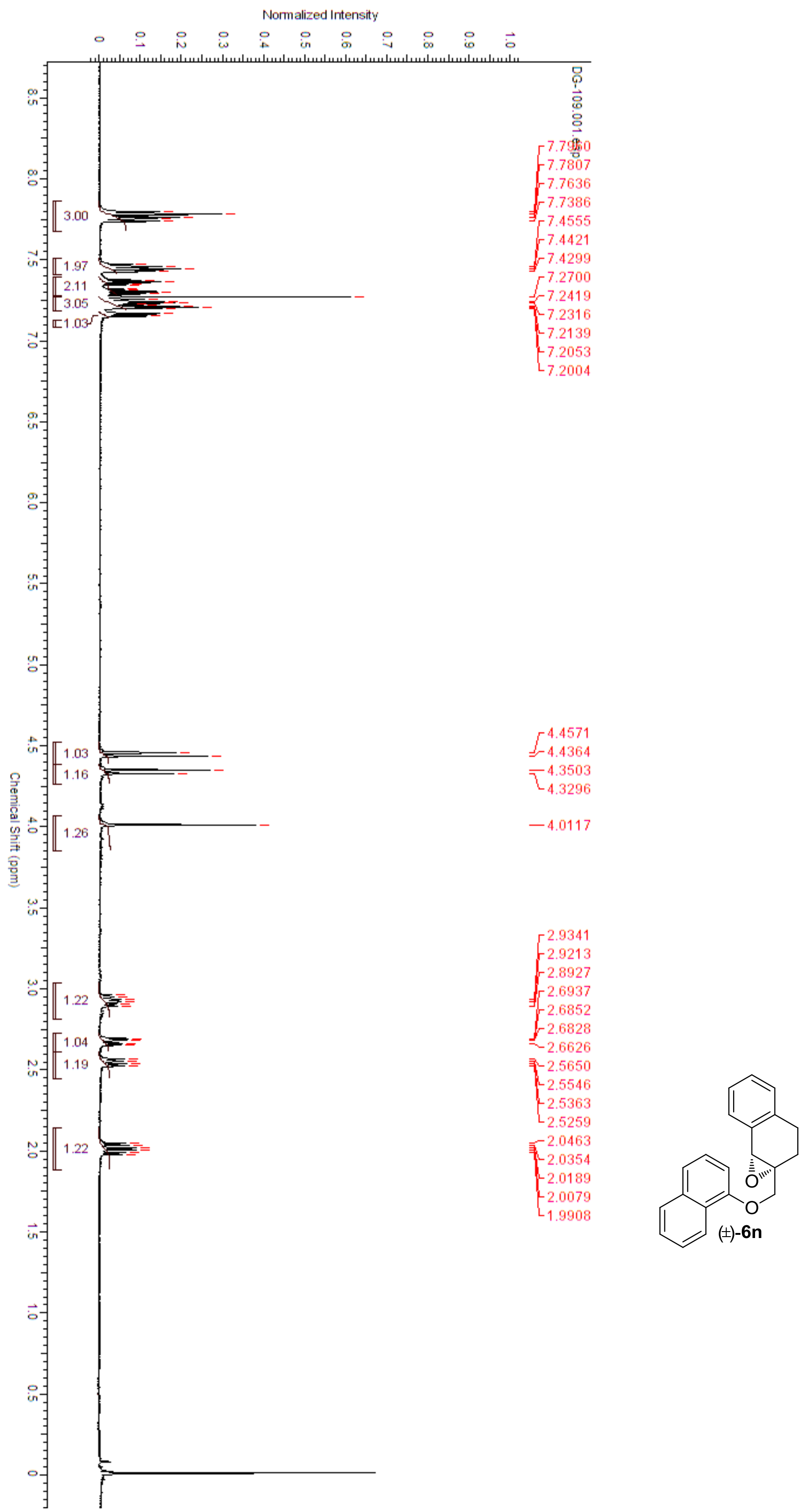

^{1}H NMR (400 MHz, $CDCl_3$) spectrum of compound (±)-**6n**.

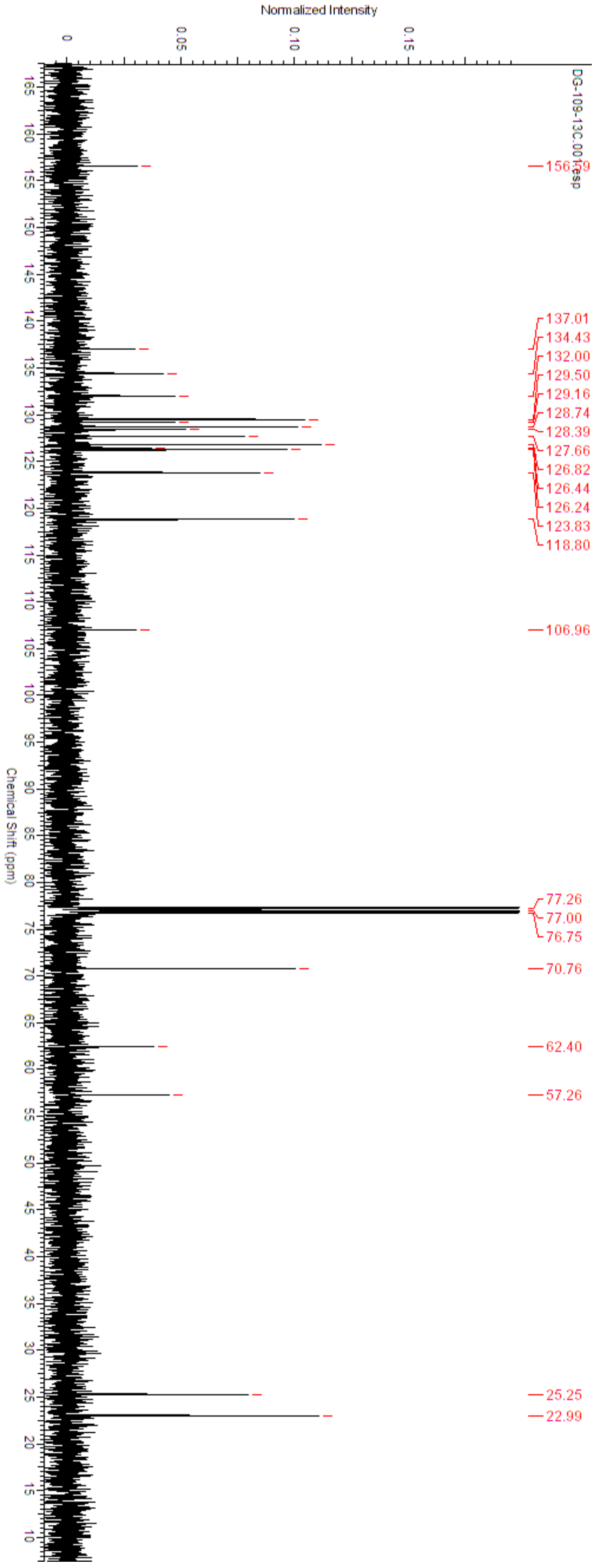

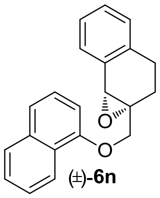

(±)-**6n**

^{13}H NMR (100 MHz, $CDCl_3$) spectrum of compound (±)-**6n**.

7. Copies of NMR Spectra of final compounds

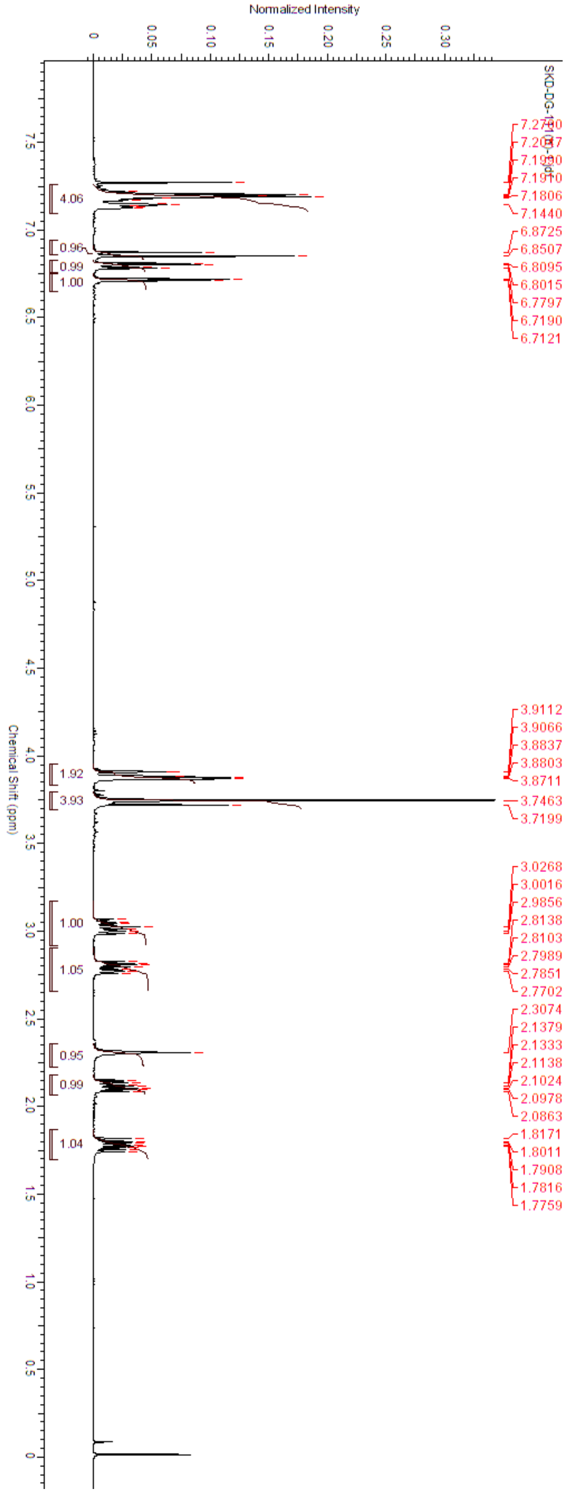

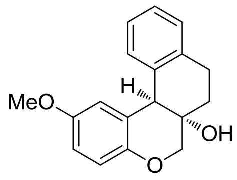

(±)-**5a**

^{1}H NMR (400 MHz, $CDCl_3$) spectrum of compound (±)-**5a**.

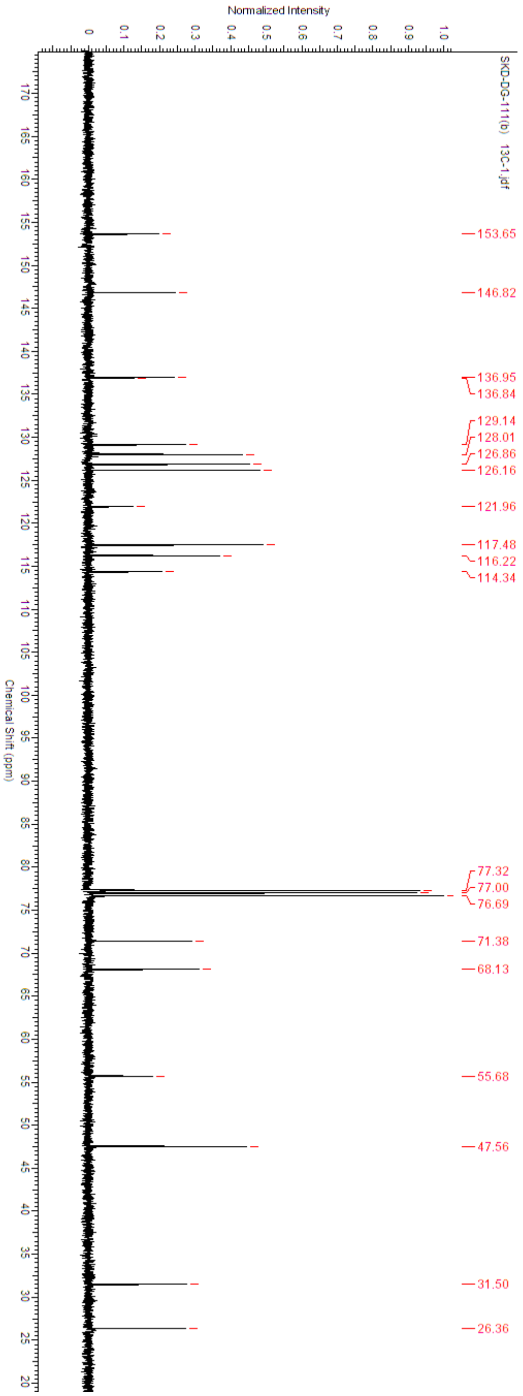

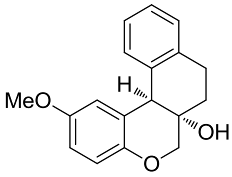

(±)-**5a**

^{13}C NMR (100 MHz, $CDCl_3$) spectrum of compound (±)-**5a**.

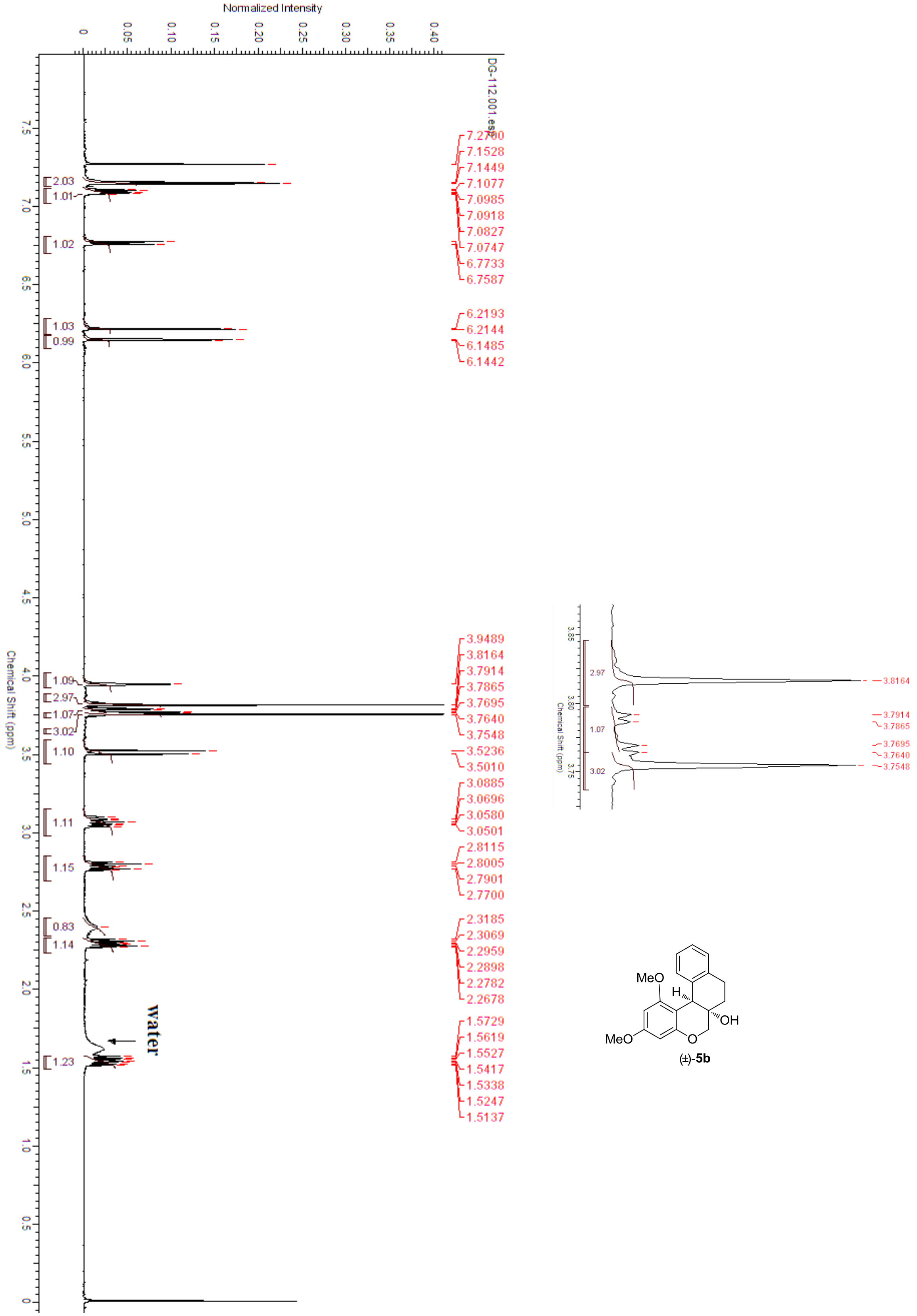

^{1}H NMR (400 MHz, $CDCl_3$) spectrum of compound (±)-**5b**.

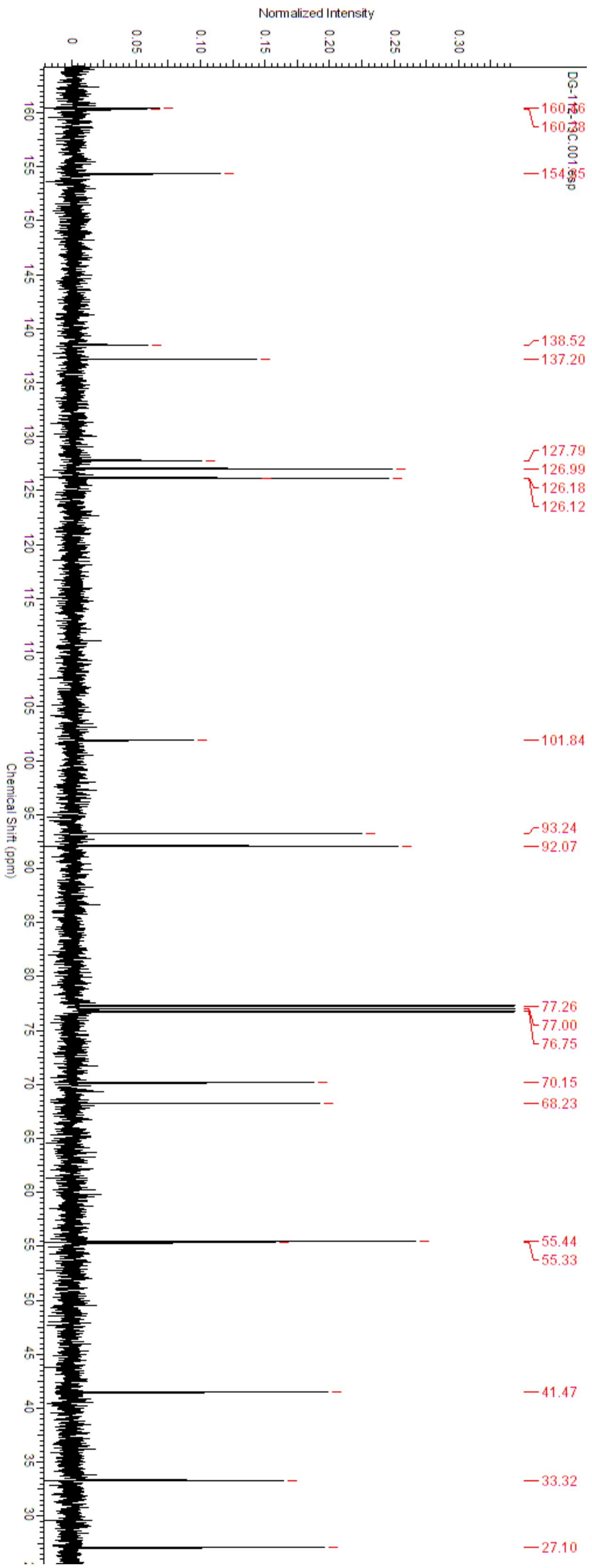

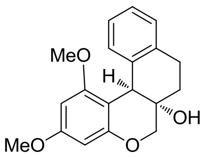

(±)-**5b**

^{13}C NMR (100 MHz, $CDCl_3$) spectrum of compound (±)-**5b**.

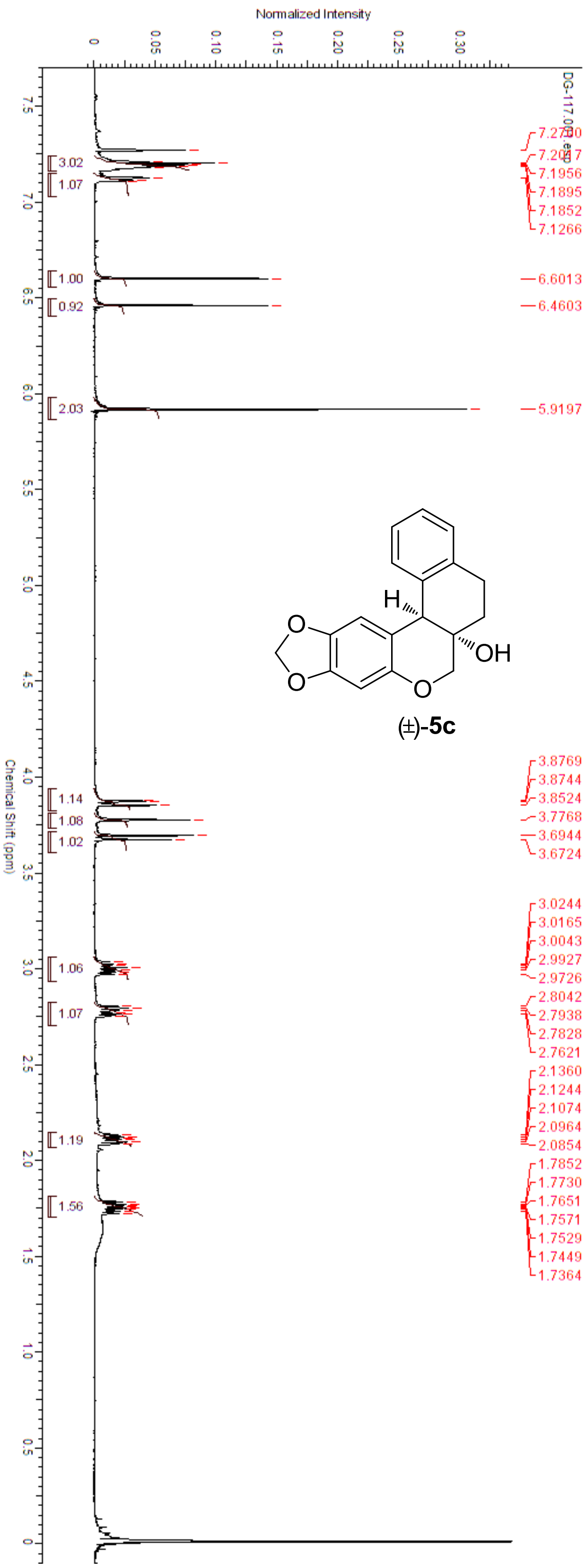

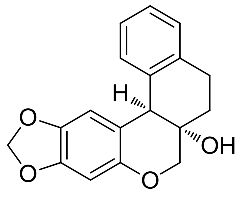

(±)-**5c**

^{1}H NMR (400 MHz, $CDCl_3$) spectrum of compound (±)-**5c**.

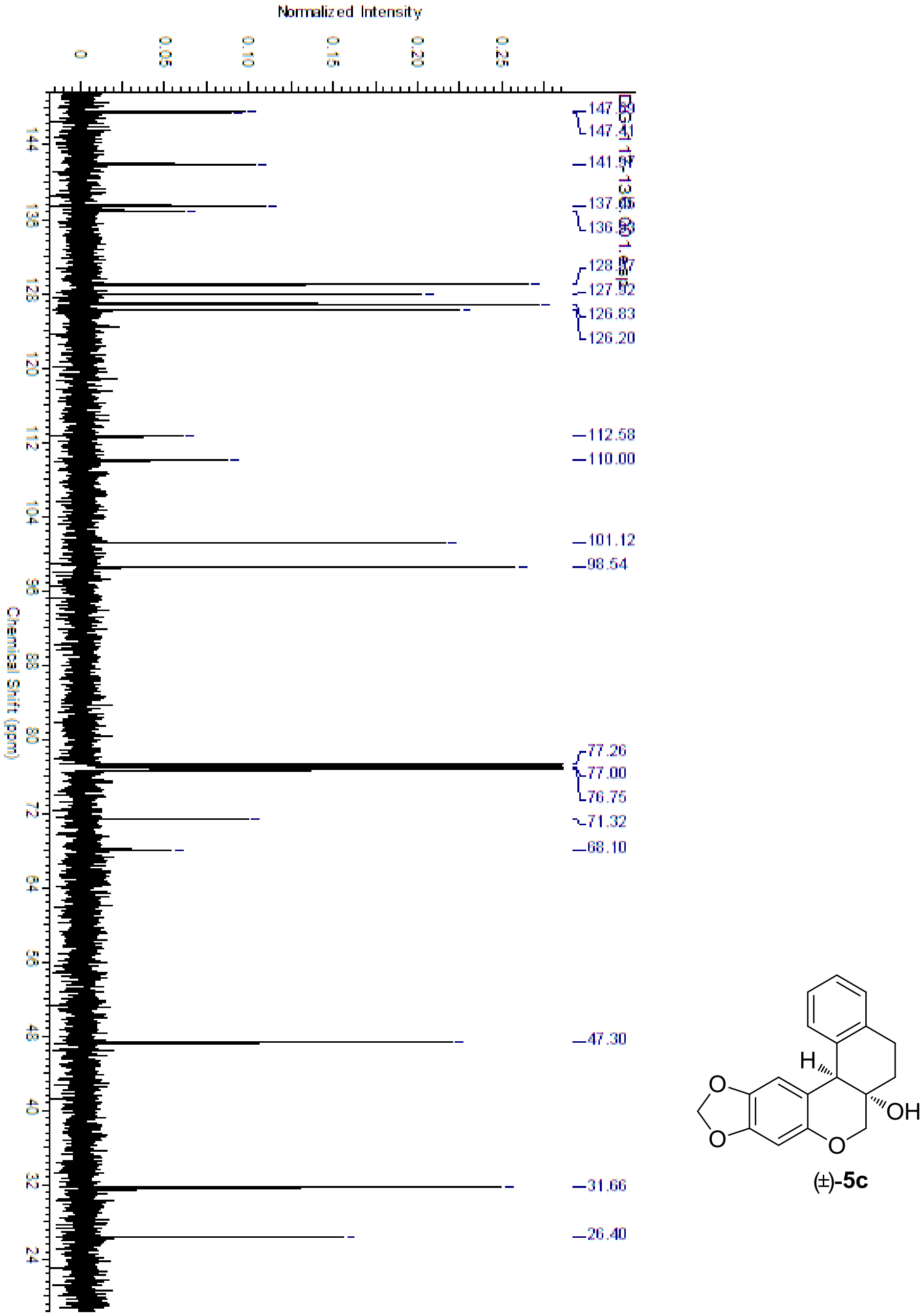

^{13}H NMR (100 MHz, $CDCl_3$) spectrum of compound (±)-**5c**.

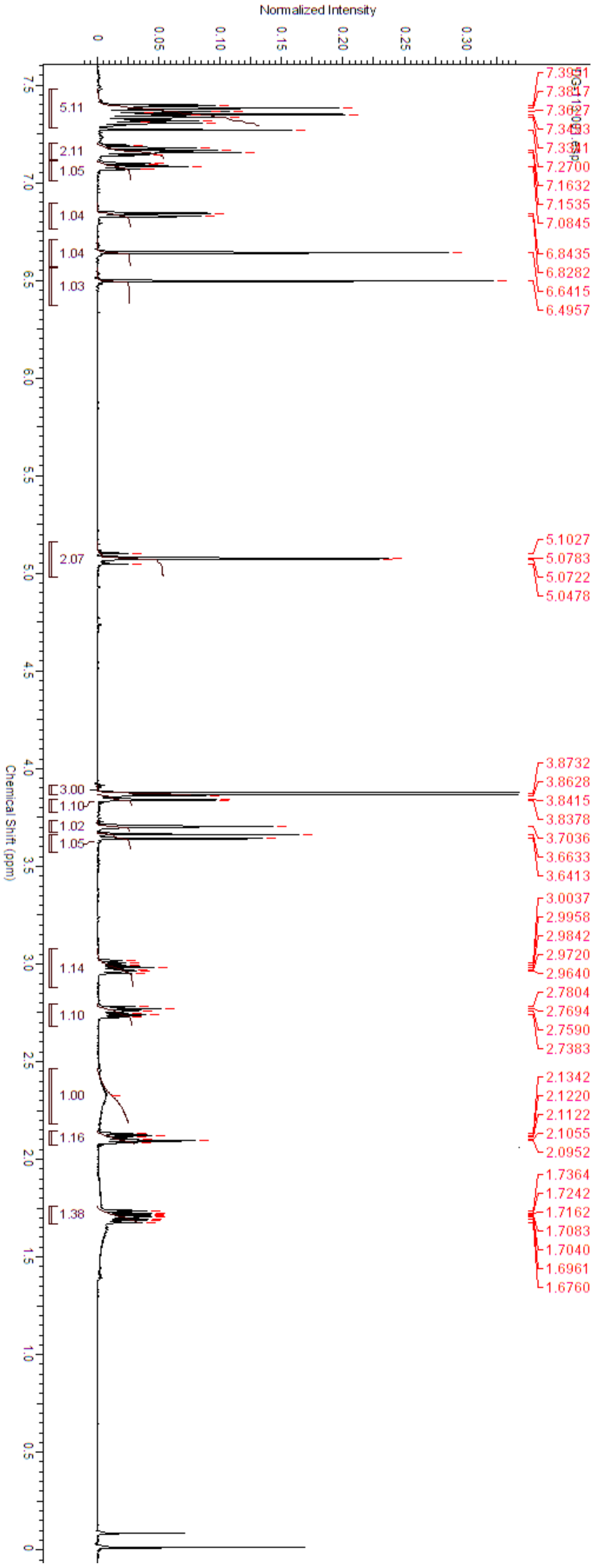

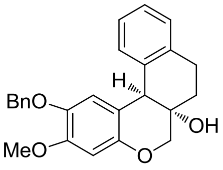

(±)-**5d**

^{1}H NMR (400 MHz, $CDCl_3$) spectrum of compound (±)-**5d**.

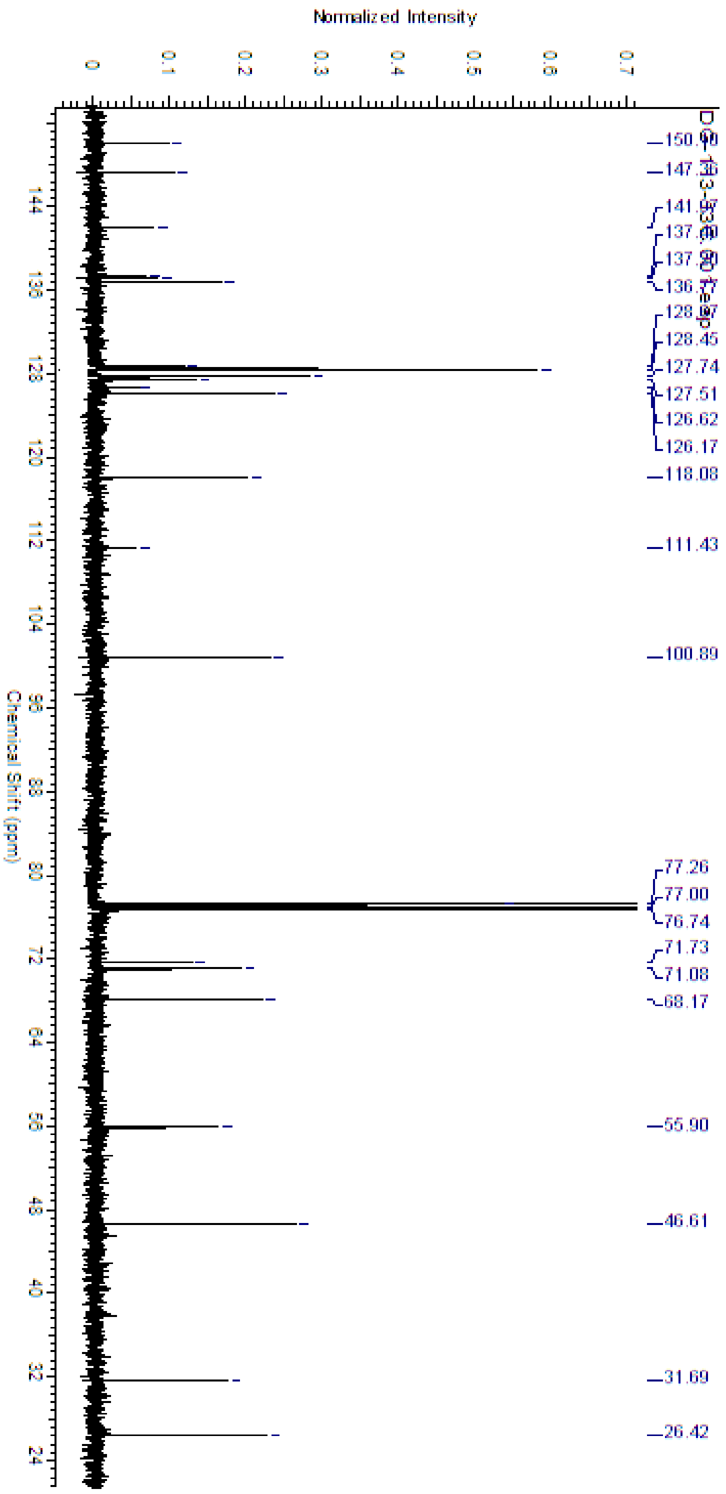

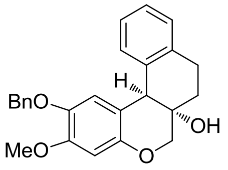

(±)-**5d**

^{13}C NMR (100 MHz, $CDCl_3$) spectrum of compound (±)-**5d**.

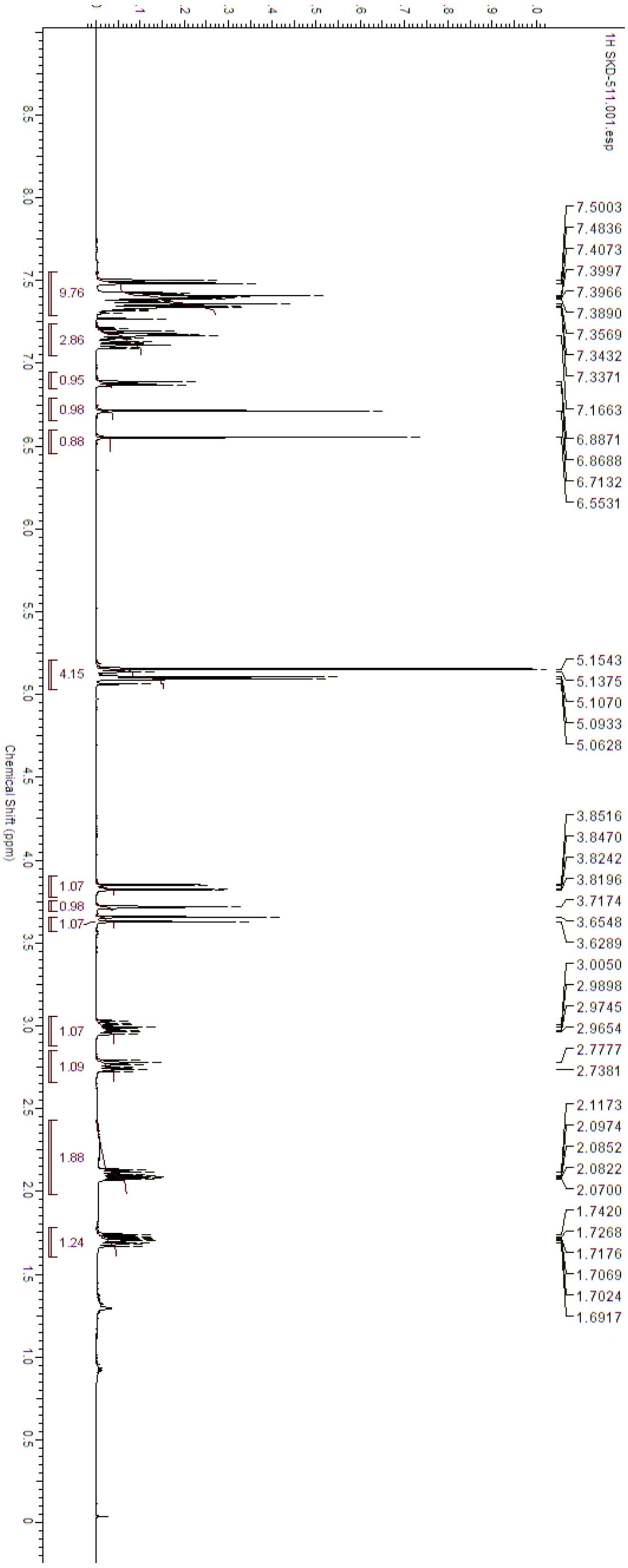

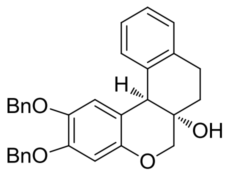

(±)-**5e**

^{1}H NMR (400 MHz, $CDCl_3$) spectrum of compound (±)-**5e**.

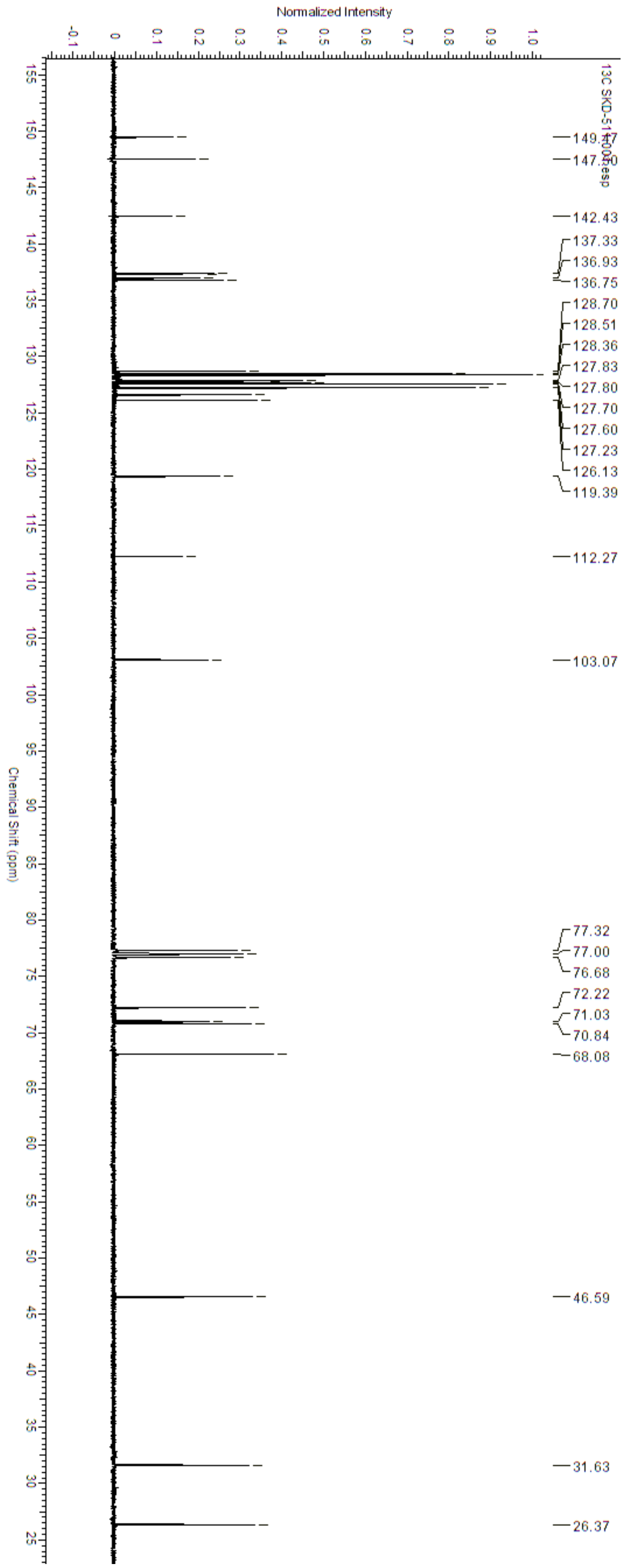

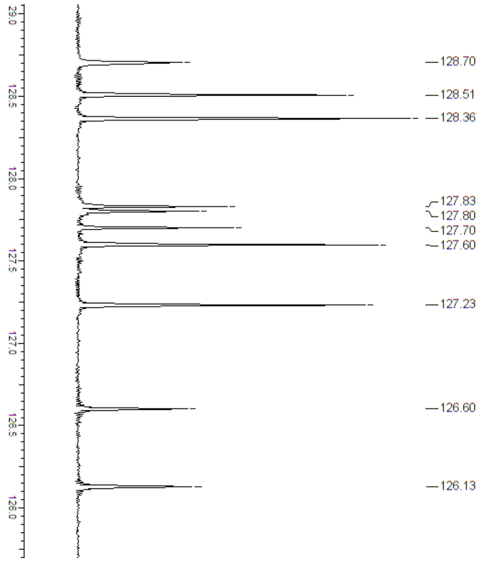

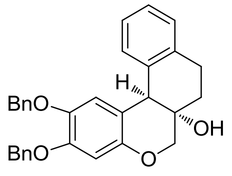

(±)-**5e**

^{13}C NMR (100 MHz, $CDCl_3$) spectrum of compound (±)-**5e**.

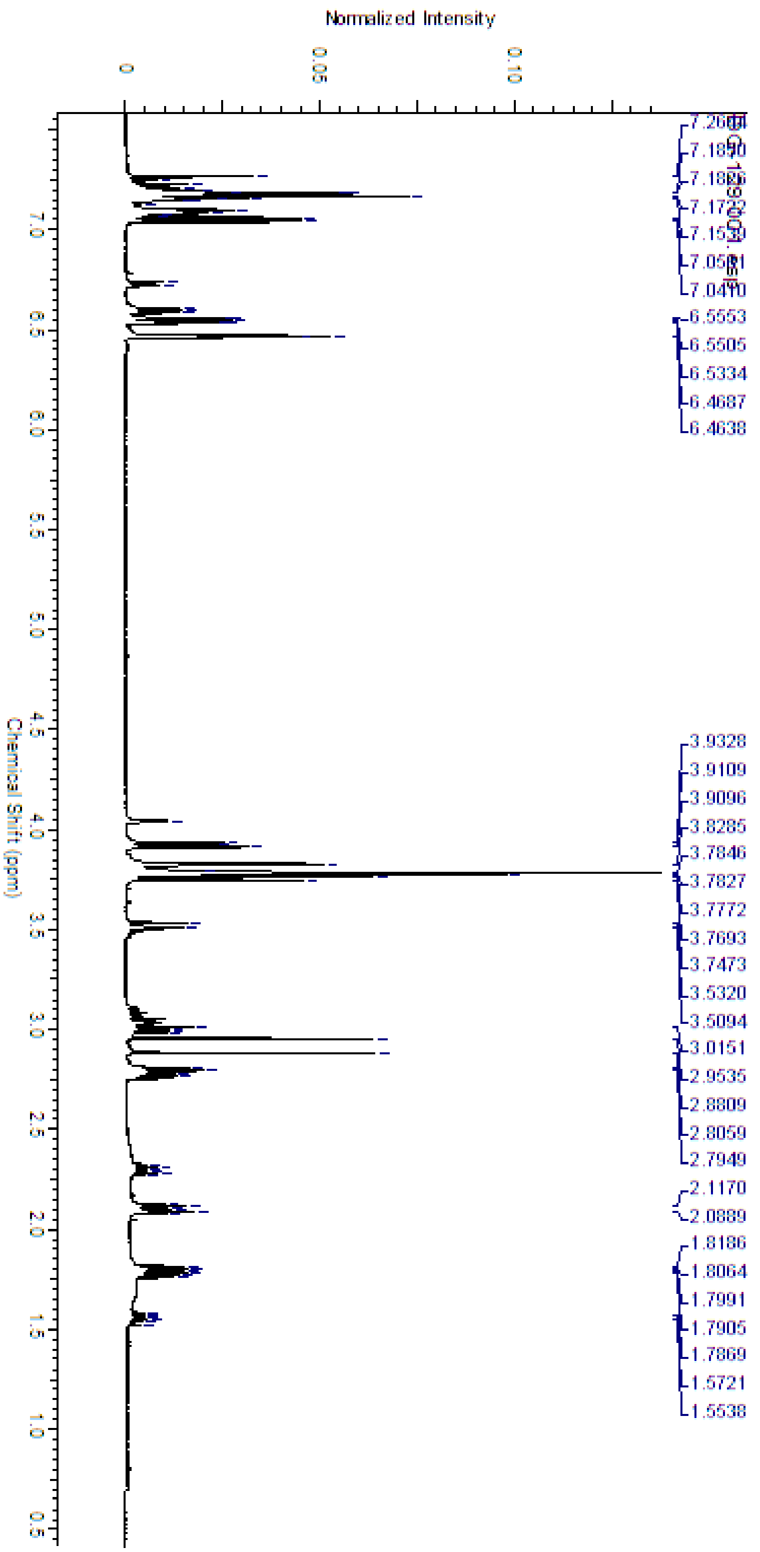

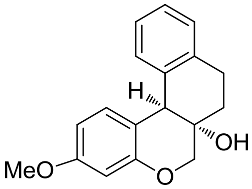

(±)-**5f**

^{1}H NMR (400 MHz, $CDCl_3$) spectrum of compound (±)-**5f** (as an inseparable mixture with the regioisomer).

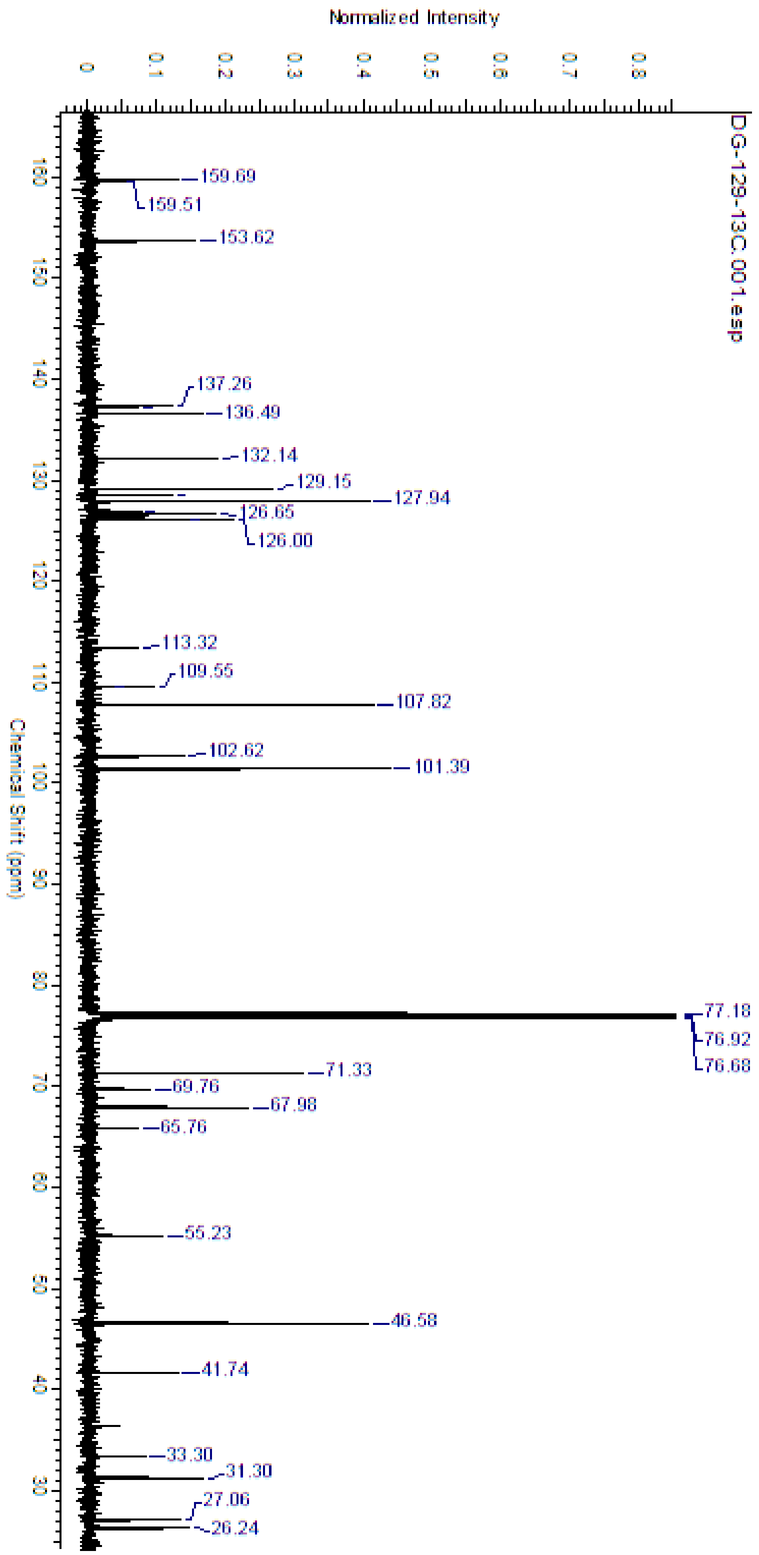

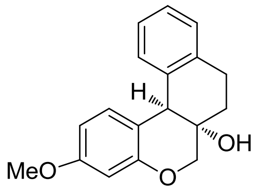

(±)-**5f**

^{13}C NMR (100 MHz, $CDCl_3$) spectrum of compound (±)-**5f** (as an inseparable mixture with the regioisomer).

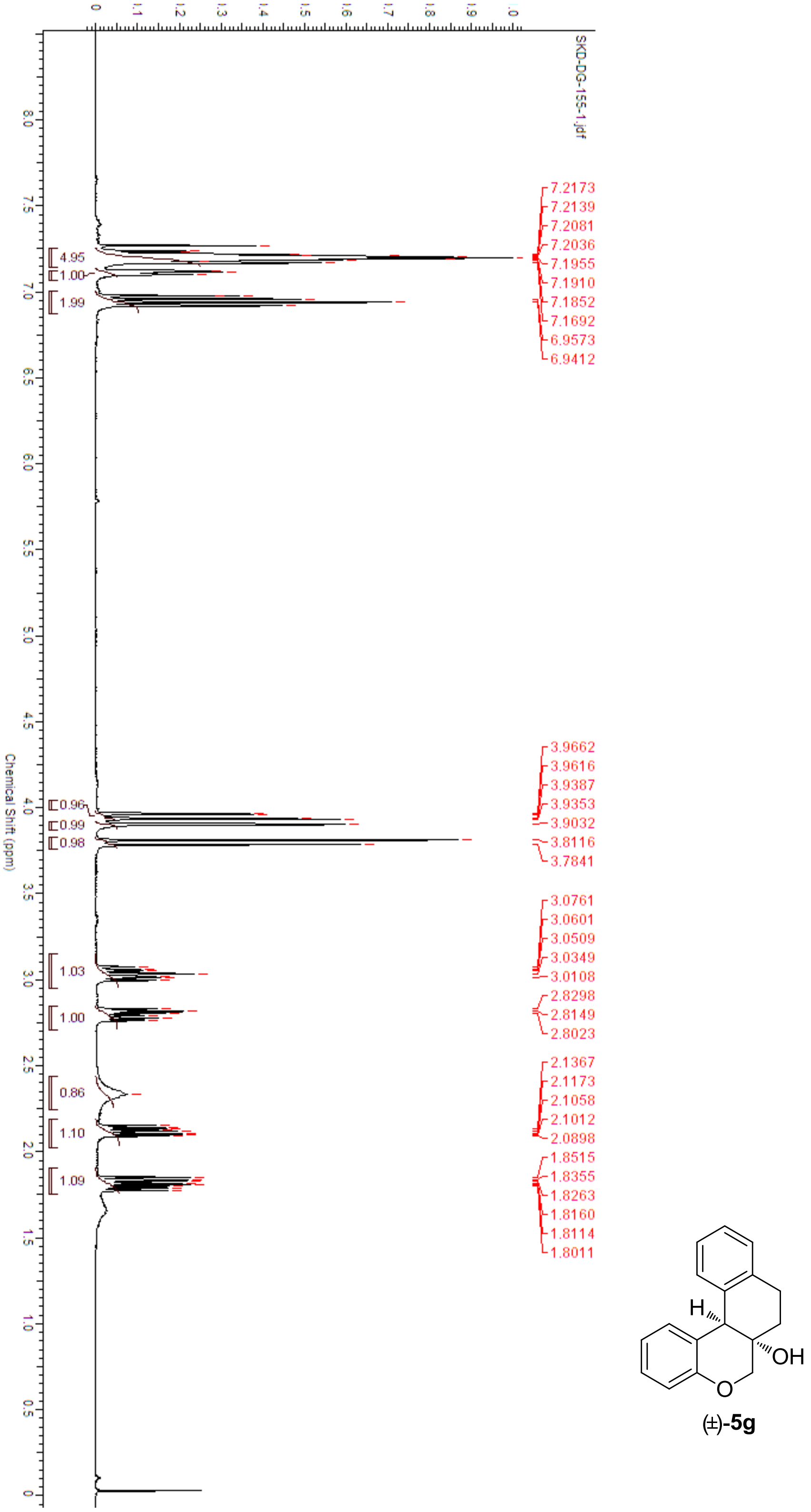

^{1}H NMR (400 MHz, $CDCl_3$) spectrum of compound (±)-**5g**.

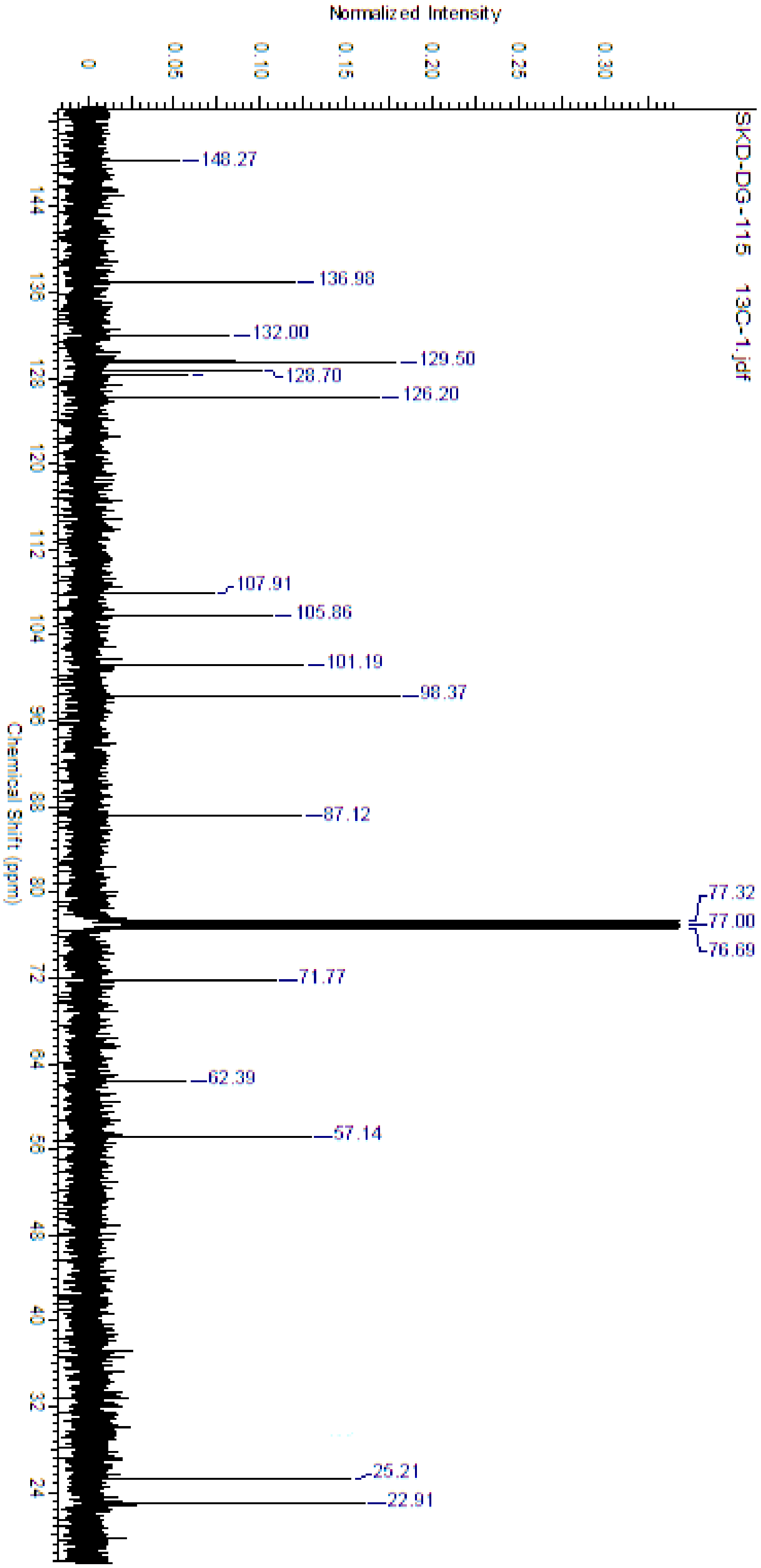

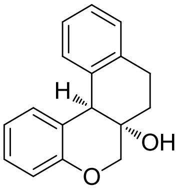

(±)-**5g**

^{13}C NMR (100 MHz, $CDCl_3$) spectrum of compound (±)-**5g**.

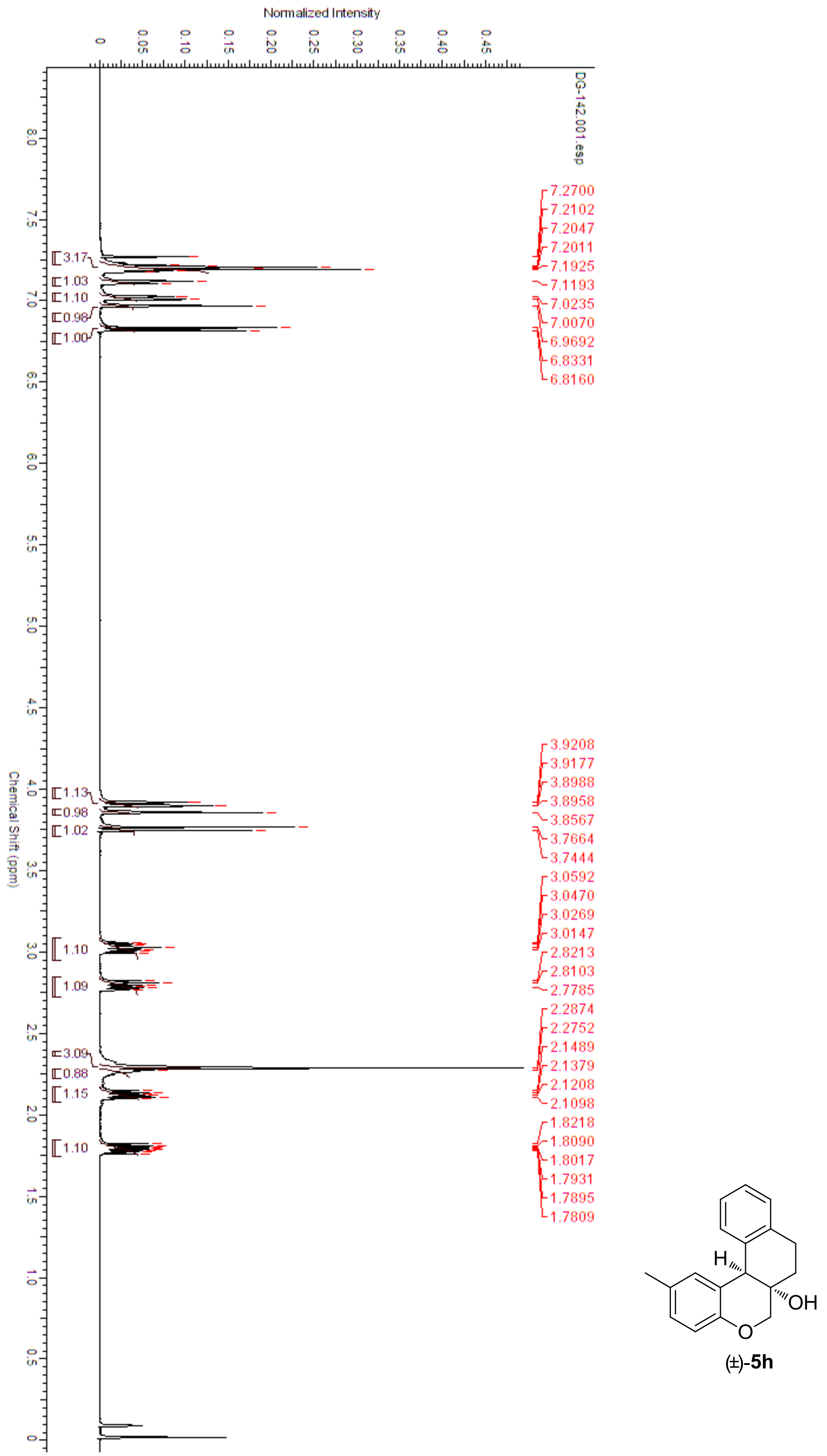

^{1}H NMR (400 MHz, $CDCl_3$) spectrum of compound (±)-**5h**.

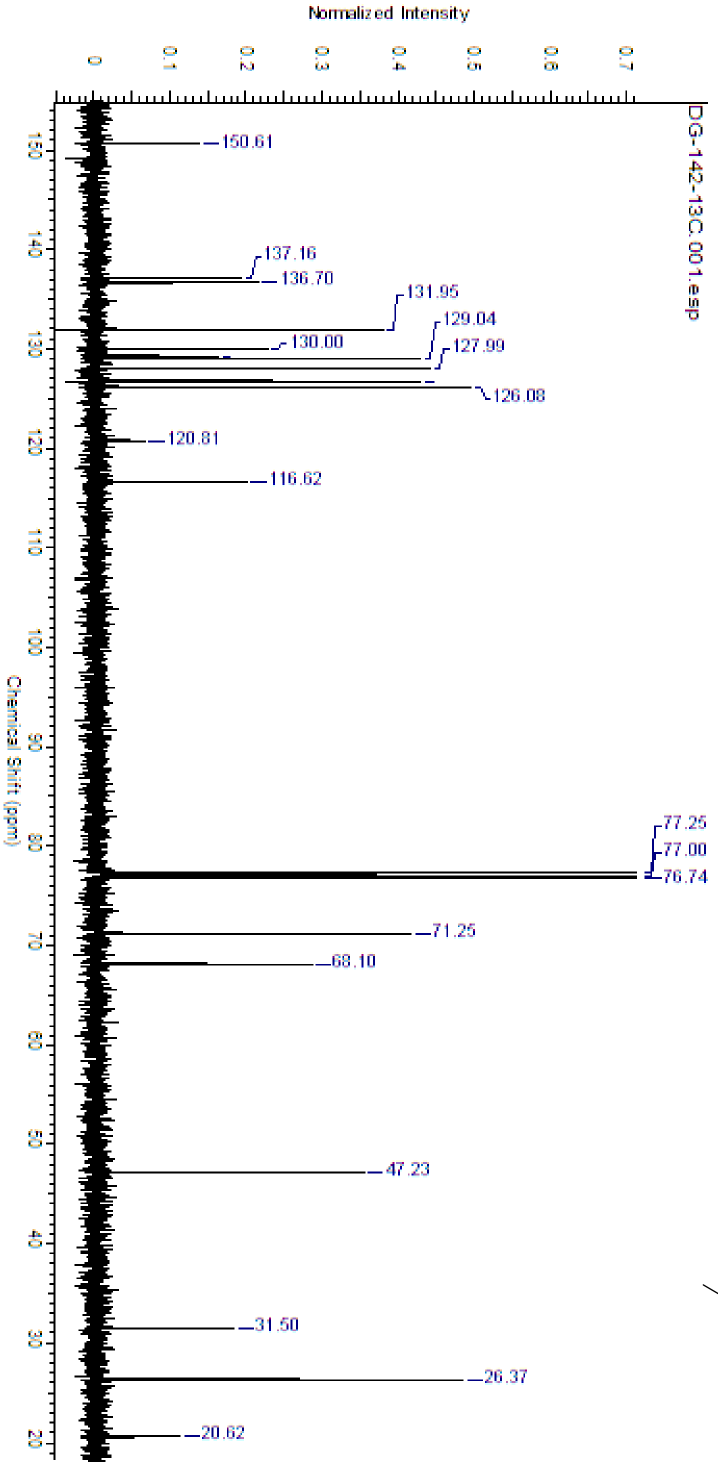

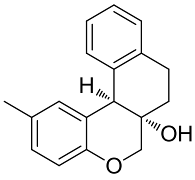

(±)-**5h**

^{13}C NMR (100 MHz, $CDCl_3$) spectrum of compound (±)-**5h**.

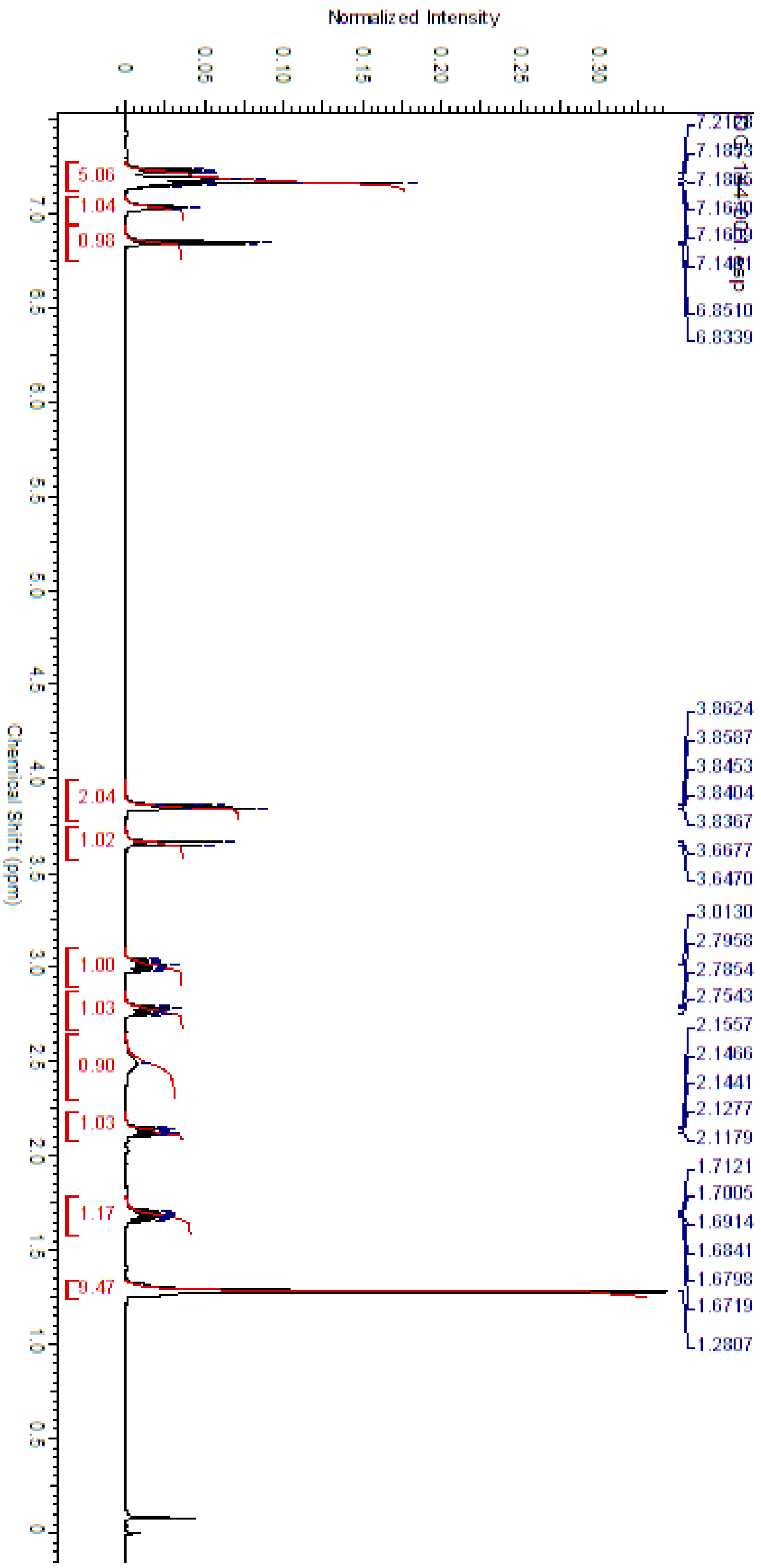

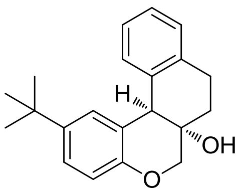

(±)-**5i**

^{1}H NMR (400 MHz, $CDCl_3$) spectrum of compound (±)-**5i**.

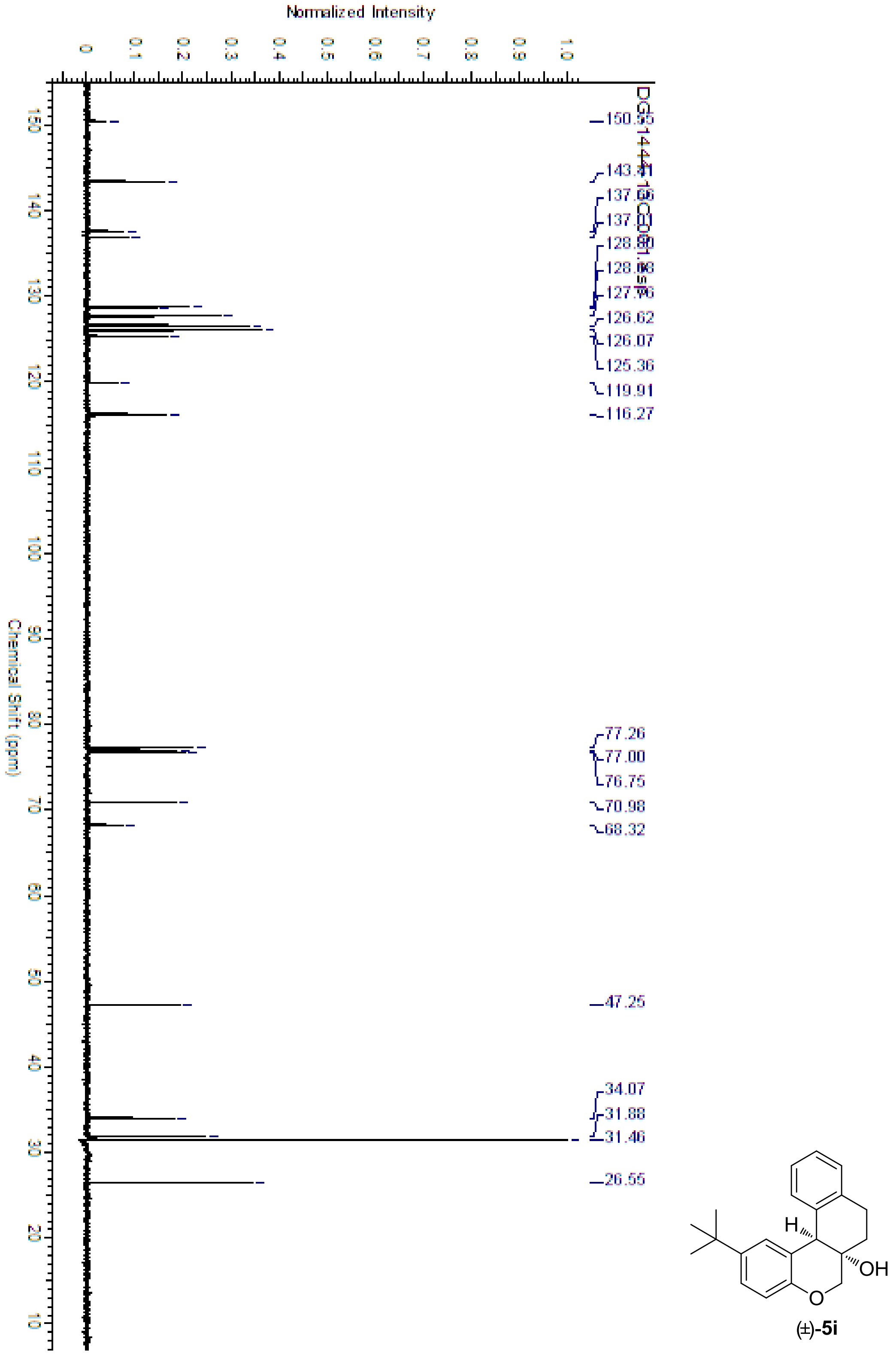

(±)-**5i**

^{13}C NMR (100 MHz, $CDCl_3$) spectrum of compound (±)-**5i**.

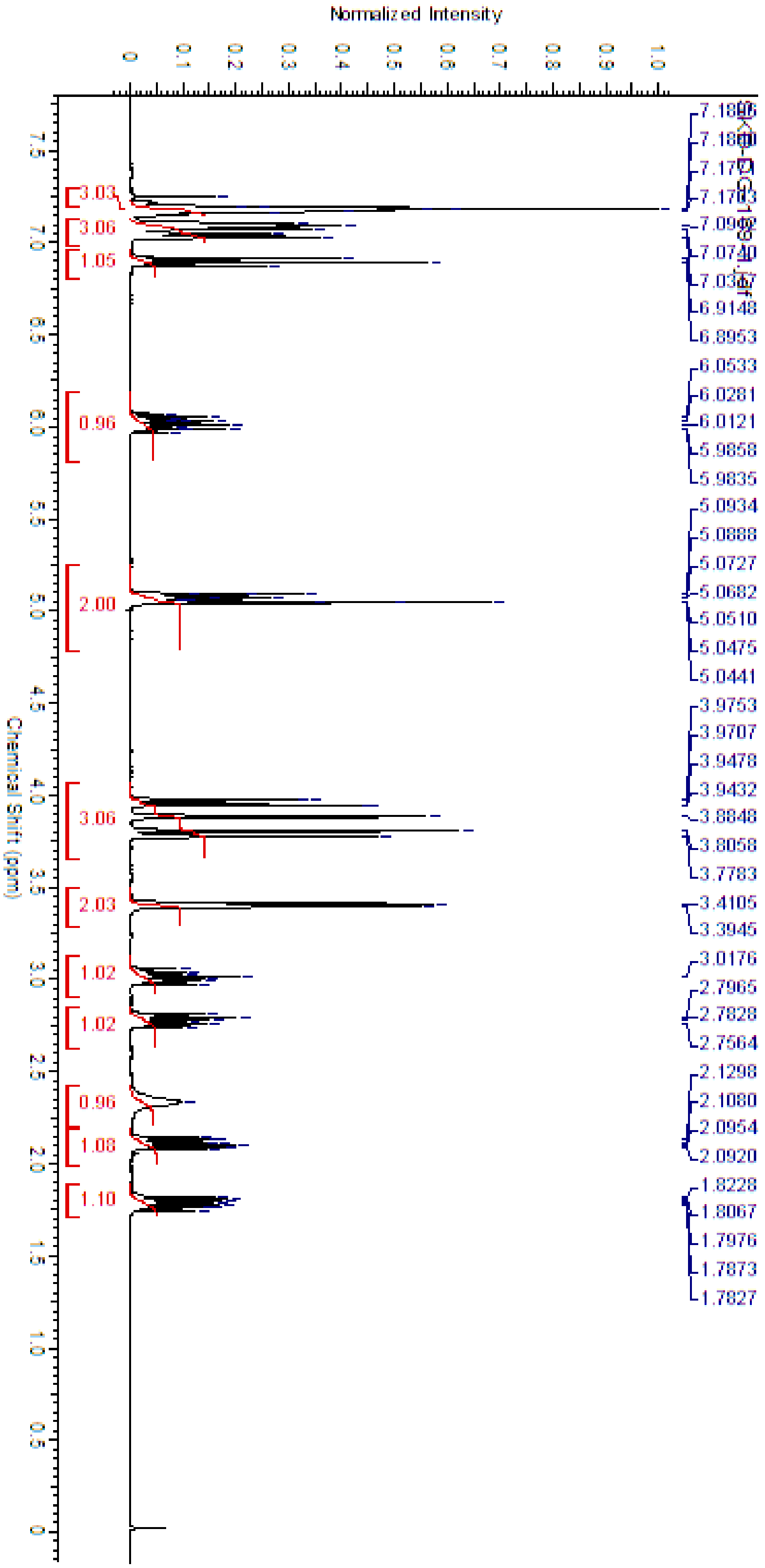

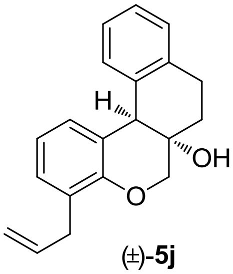

(±)-**5j**

^{1}H NMR (400 MHz, $CDCl_3$) spectrum of compound (±)-**5j**.

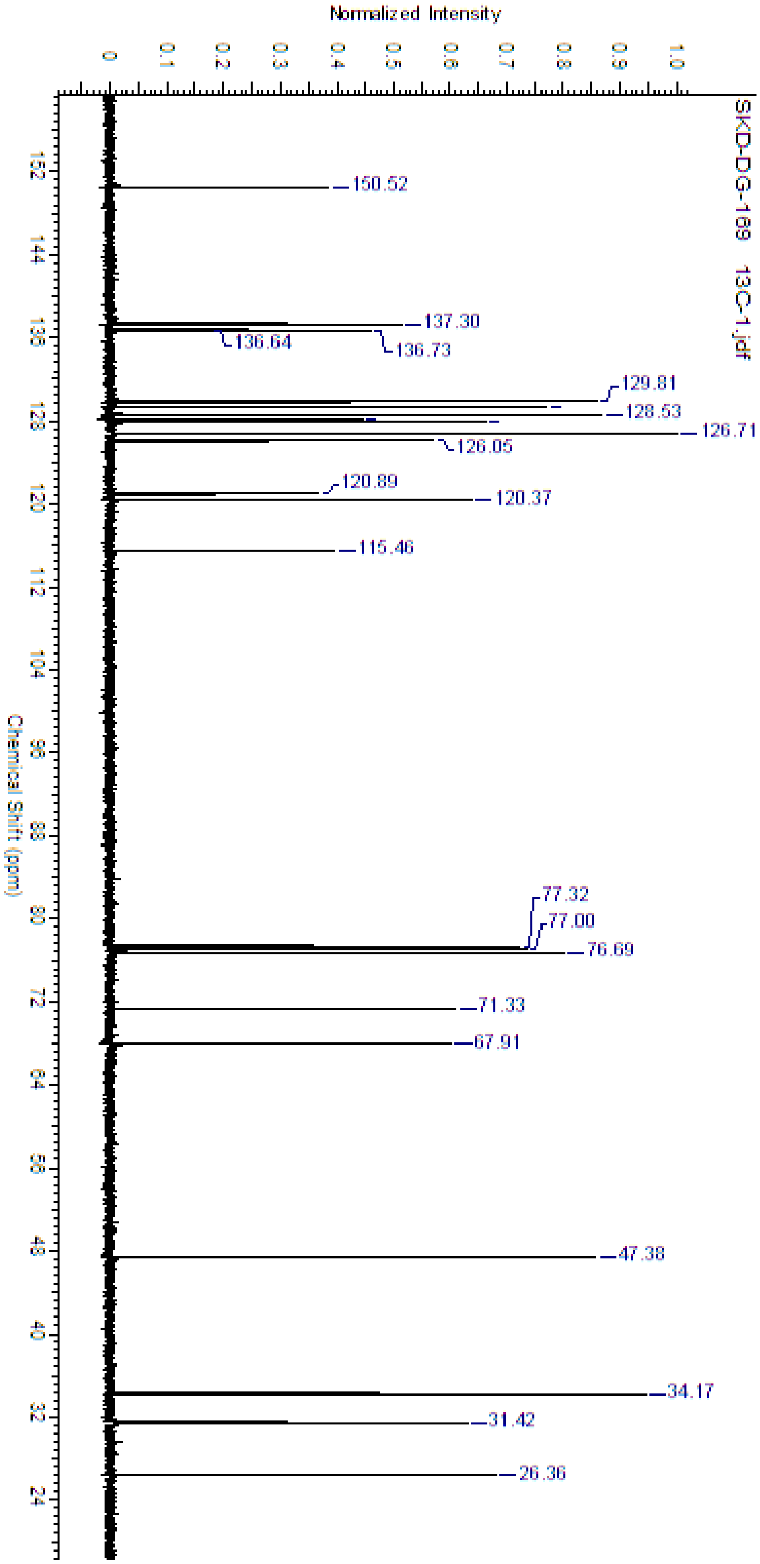

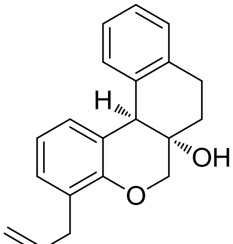

(±)-**5j**

^{13}C NMR (100 MHz, $CDCl_3$) spectrum of compound (±)-**5j**.

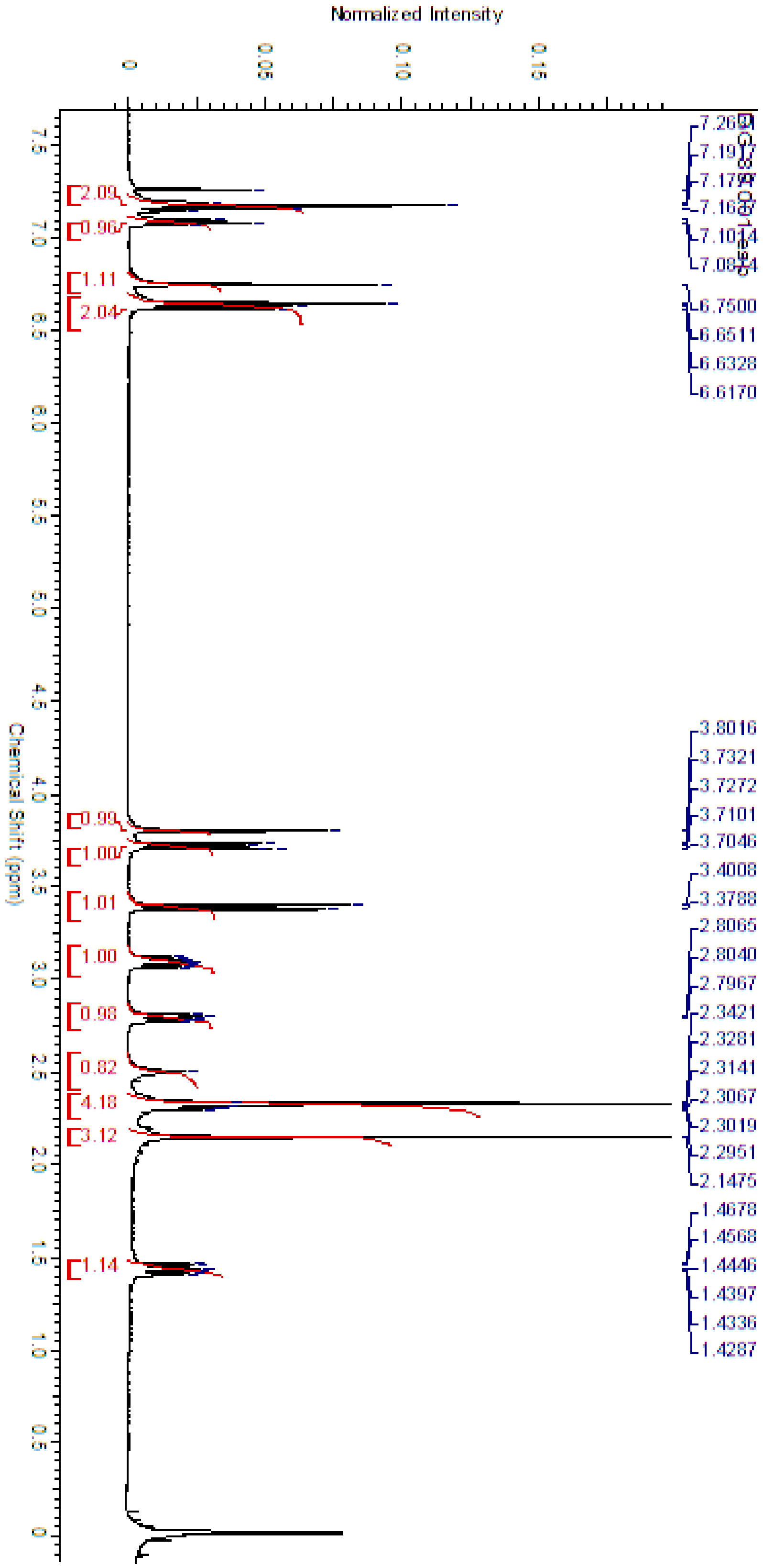

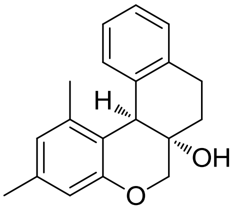

(±)-**5k**

^{1}H NMR (400 MHz, $CDCl_3$) spectrum of compound (±)-**5k**.

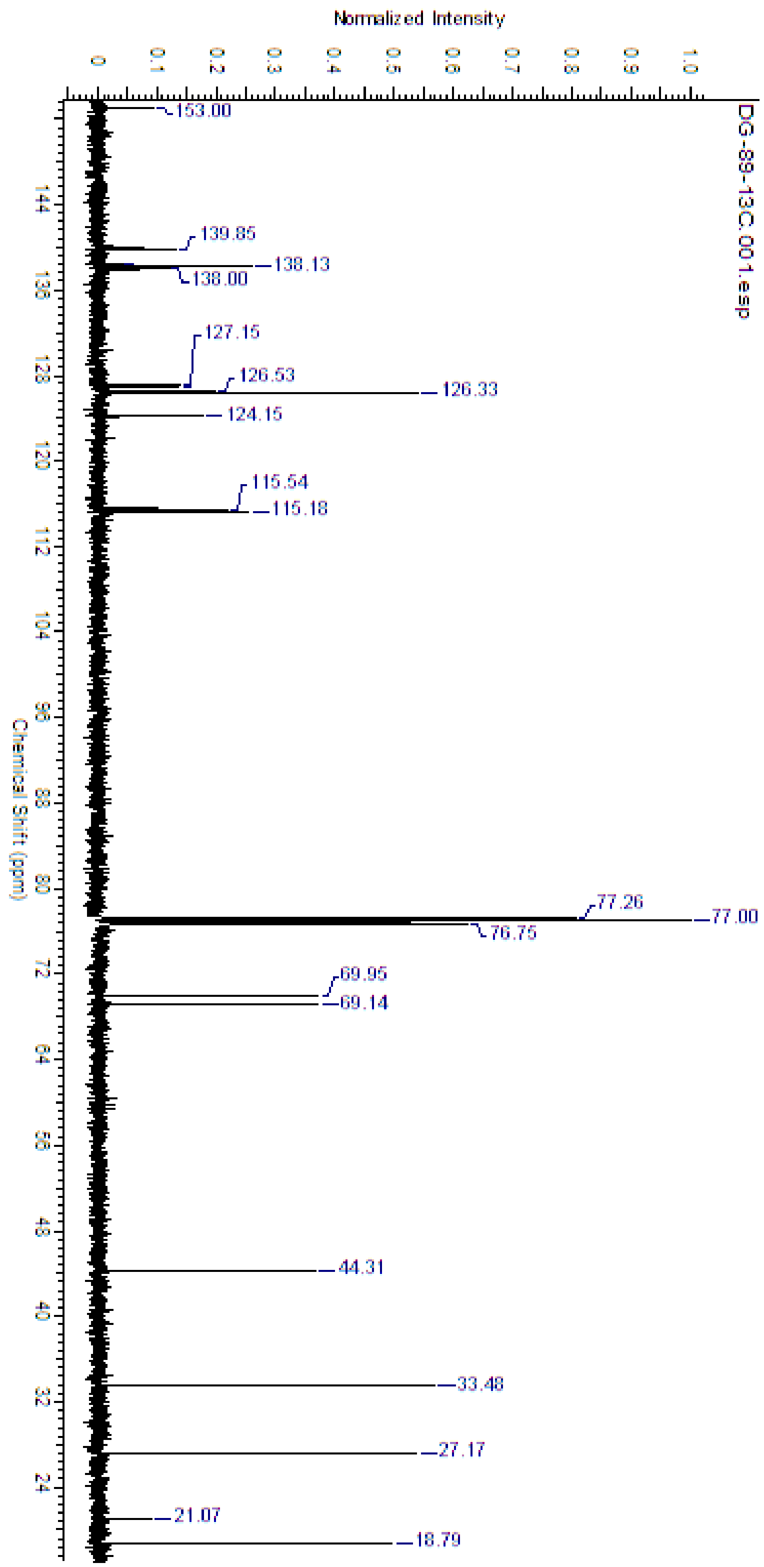

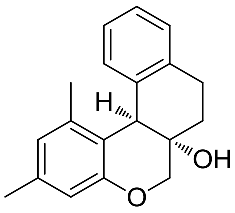

(±)-**5k**

^{13}C NMR (100 MHz, $CDCl_3$) spectrum of compound (±)-**5k**.

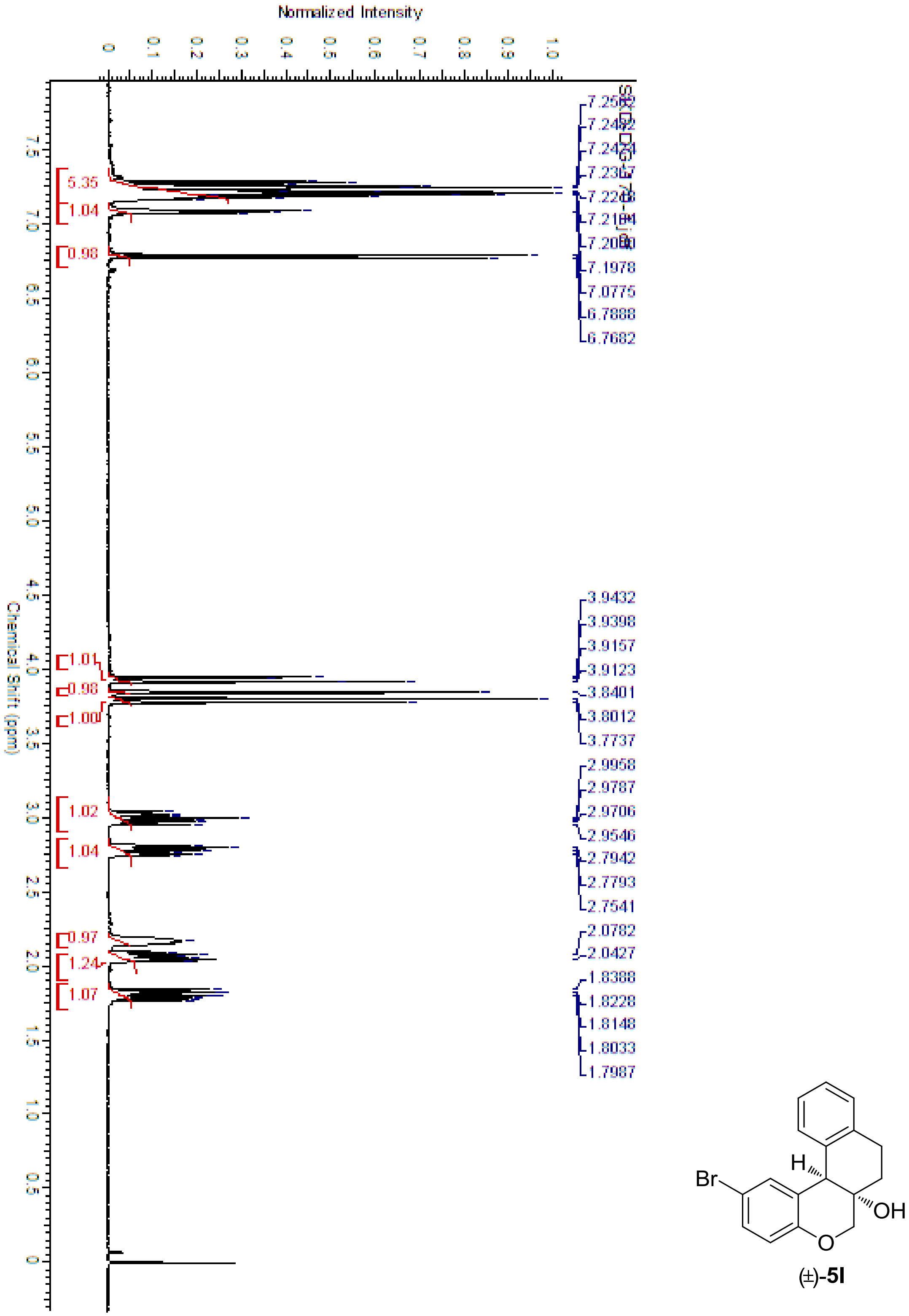

(±)-**5l**

^{1}H NMR (400 MHz, $CDCl_3$) spectrum of compound (±)-**5l**.

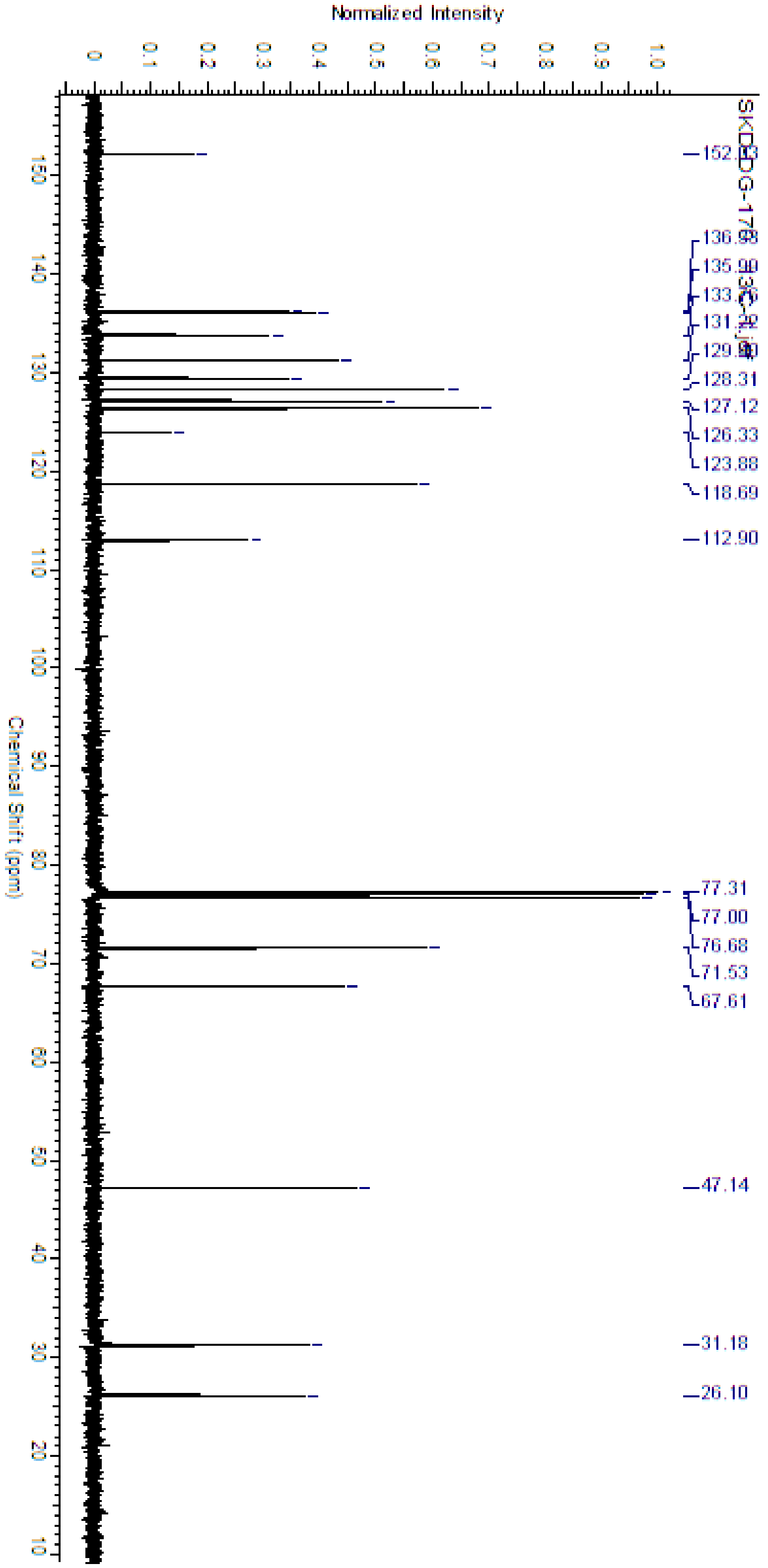

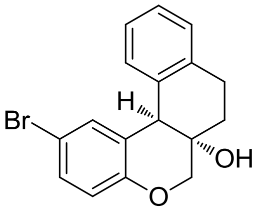

(±)-**5l**

^{13}C NMR (100 MHz, $CDCl_3$) spectrum of compound (±)-**5l**.

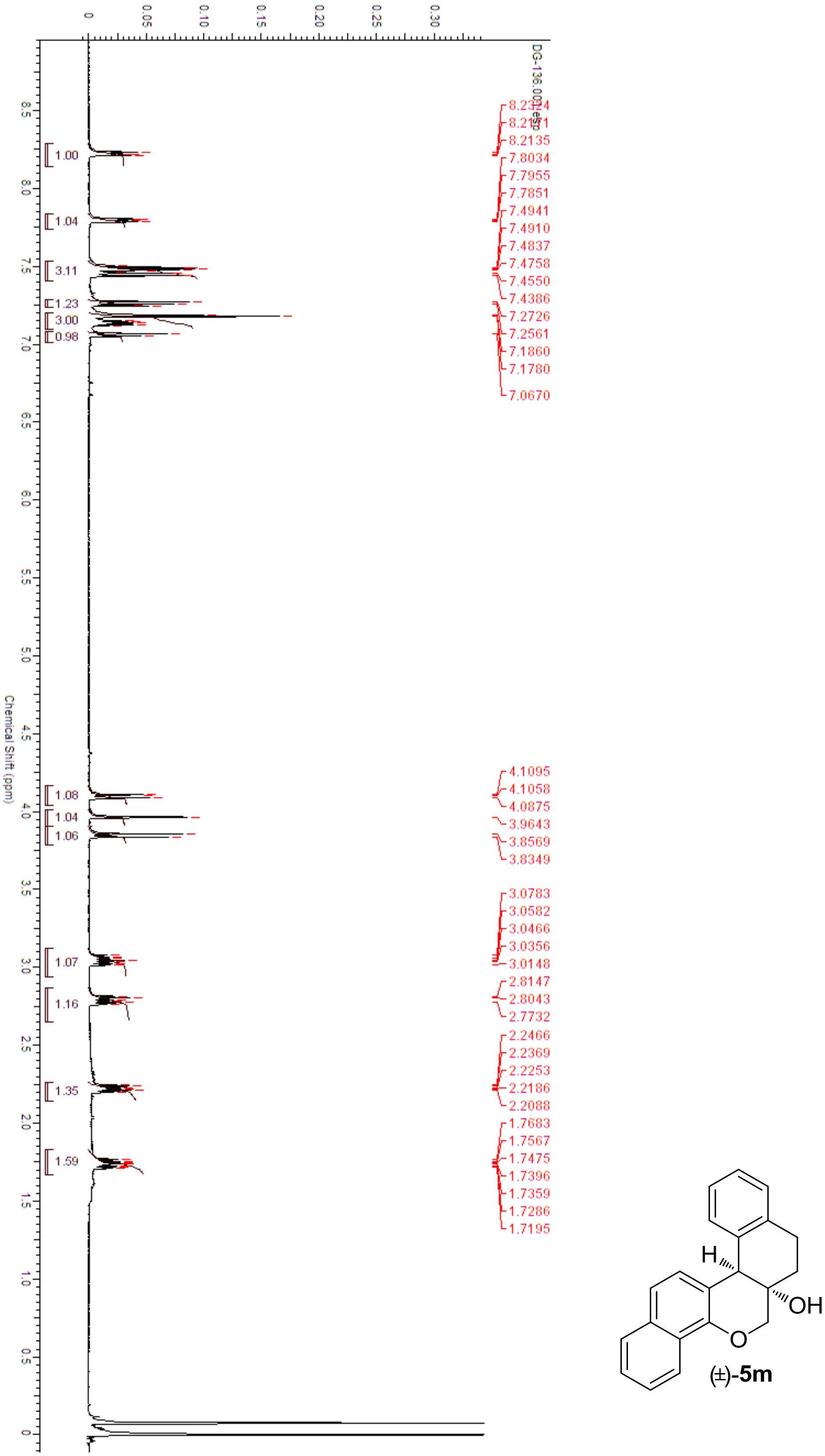

^{1}H NMR (400 MHz, $CDCl_3$) spectrum of compound (±)-**5m**.

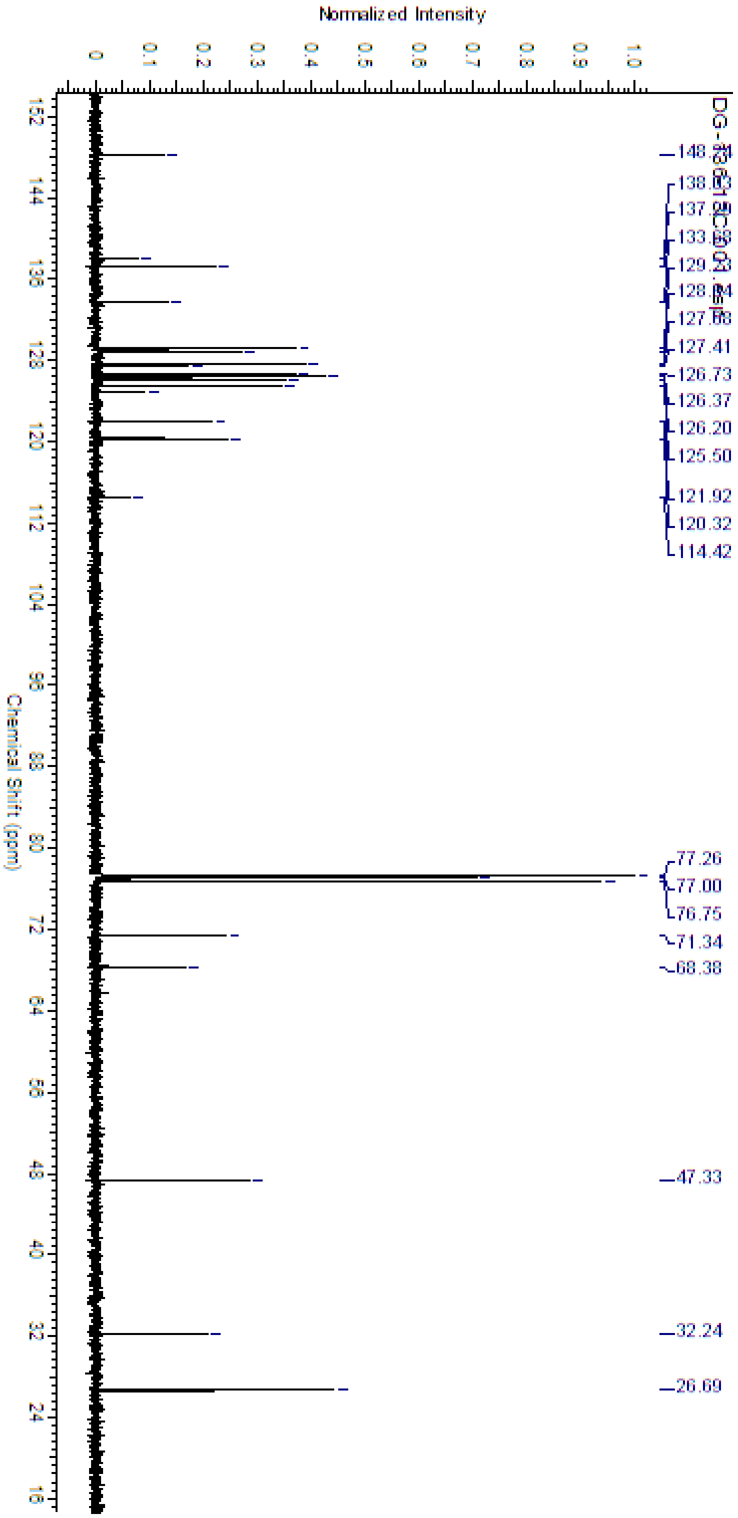

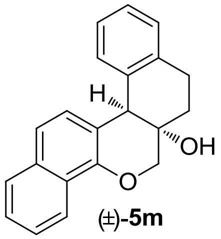

(±)-**5m**

^{13}C NMR (100 MHz, $CDCl_3$) spectrum of compound (±)-**5m**.

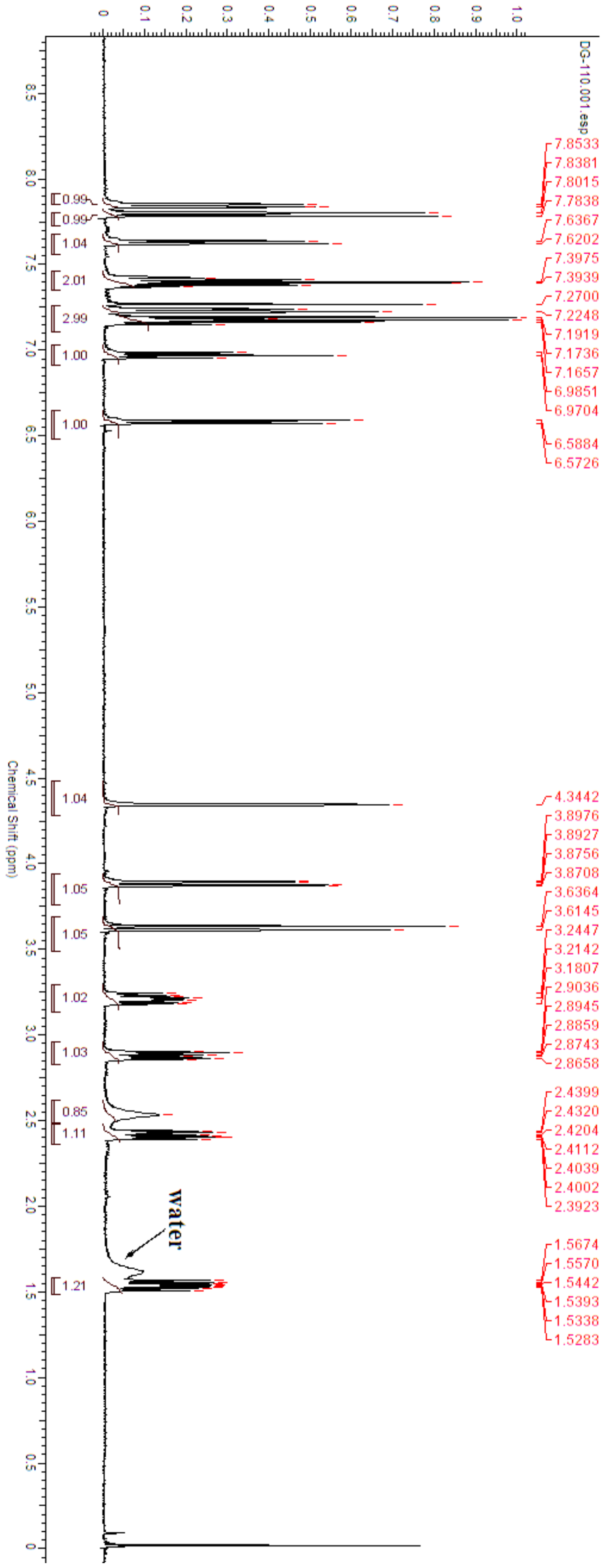

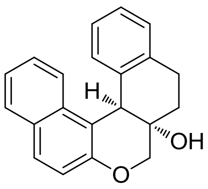

(±)-**5n**

^{1}H NMR (400 MHz, $CDCl_3$) spectrum of compound (±)-**5n**.

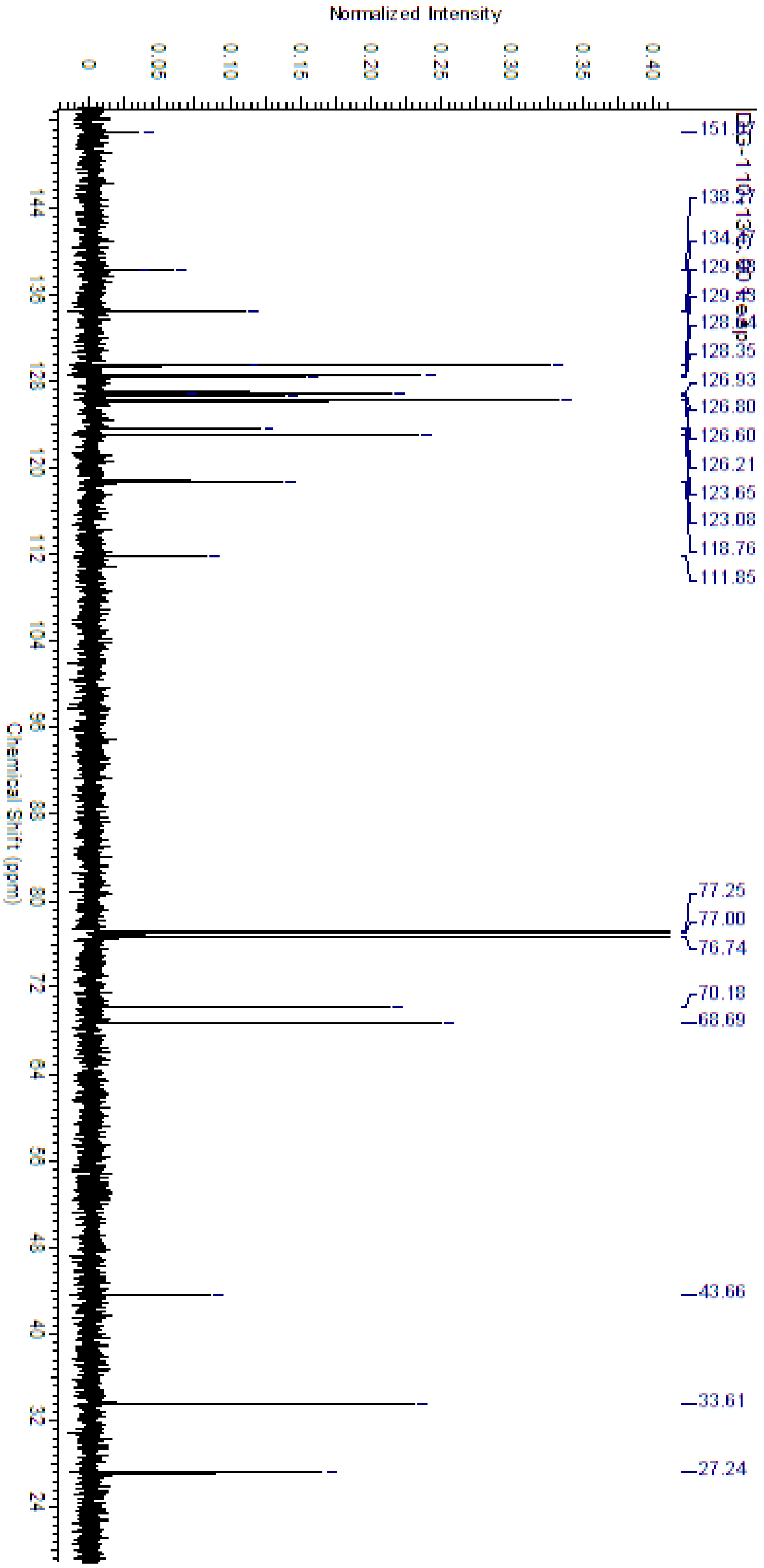

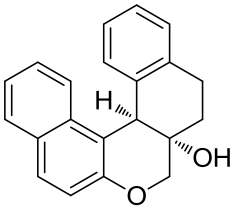

(±)-**5n**

^{13}C NMR (100 MHz, $CDCl_3$) spectrum of compound (±)-**5n**.

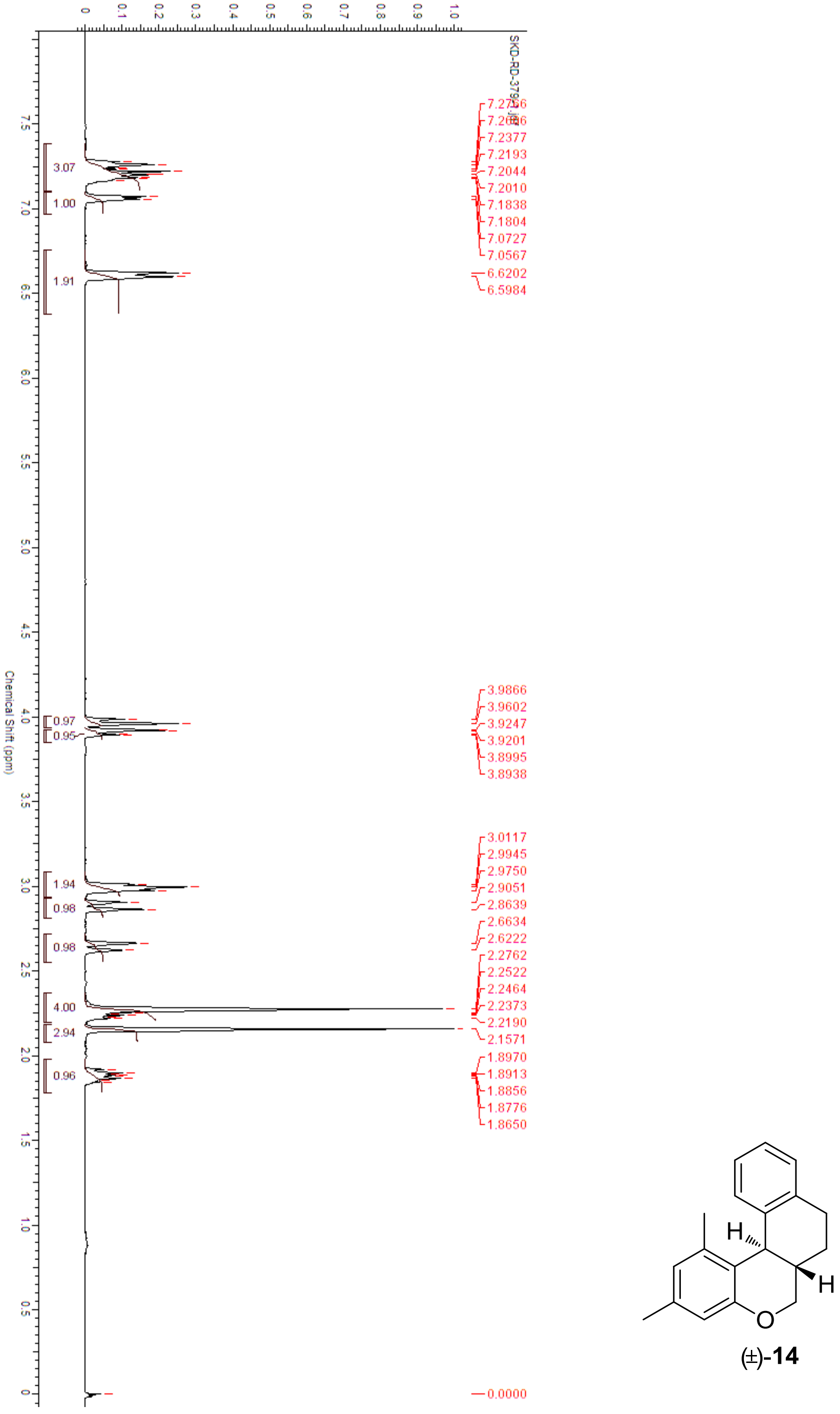

(±)-**14**

^{1}H NMR (400 MHz, $CDCl_3$) spectrum of compound (±)-**14**.

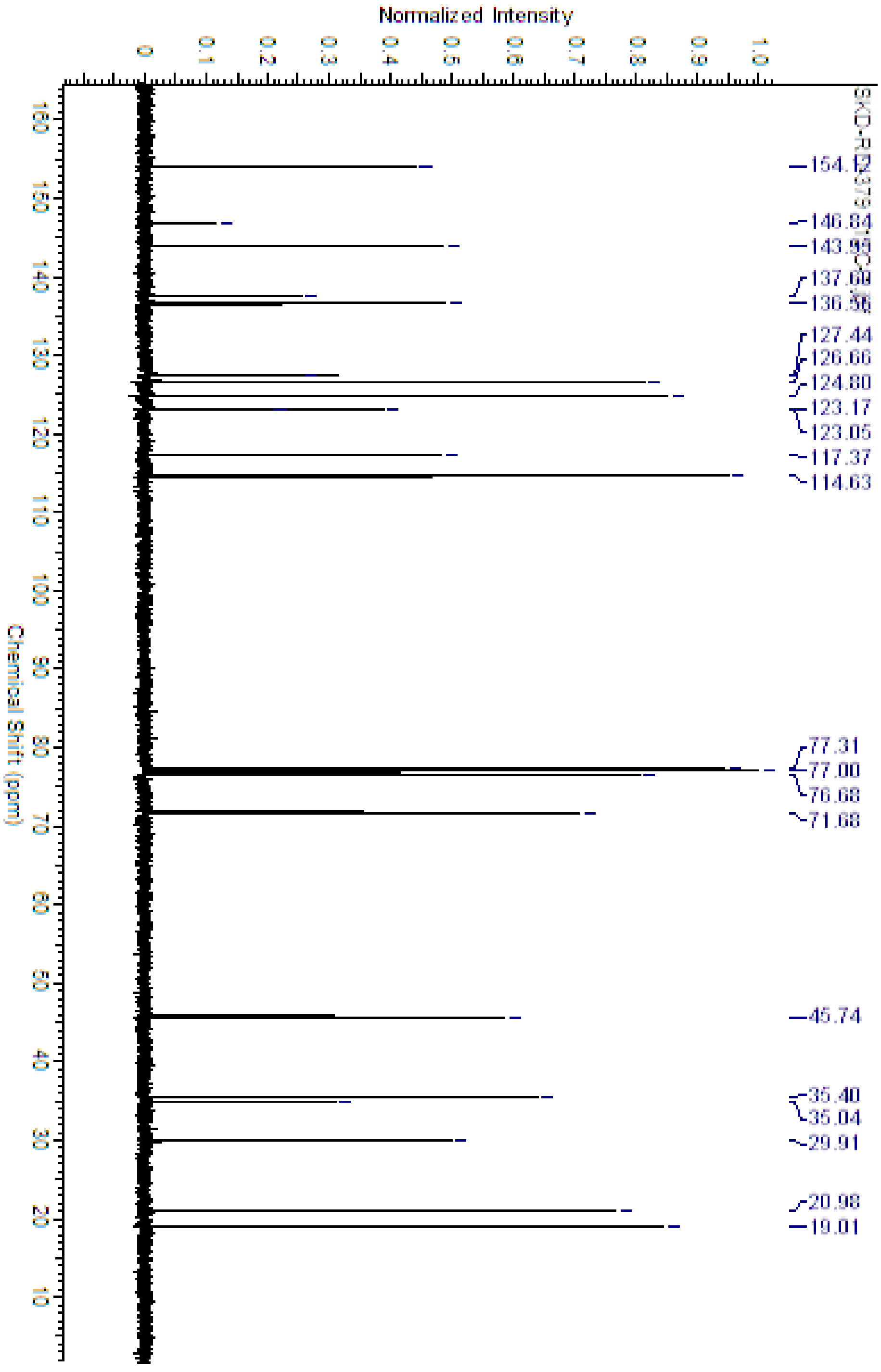

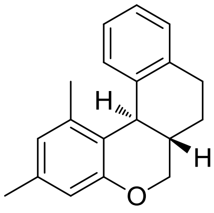

(±)-**14**

^{13}C NMR (100 MHz, $CDCl_3$) spectrum of compound (±)-**14**.
